# CoVSense: Ultrasensitive Nucleocapsid Antigen Immunosensor for Rapid Clinical Detection of Wildtype and Variant SARS‐CoV‐2

**DOI:** 10.1002/advs.202206615

**Published:** 2023-03-30

**Authors:** Razieh Salahandish, Jae Eun Hyun, Fatemeh Haghayegh, Hamed Osouli Tabrizi, Shirin Moossavi, Sultan Khetani, Giancarlo Ayala‐Charca, Byron M. Berenger, Yan Dong Niu, Ebrahim Ghafar‐Zadeh, Amir Sanati Nezhad

**Affiliations:** ^1^ BioMEMS and Bioinspired Microfluidic Laboratory Department of Biomedical Engineering University of Calgary Calgary AB T2N 1N4 Canada; ^2^ Department of Mechanical and Manufacturing Engineering University of Calgary Calgary AB T2N 1N4 Canada; ^3^ Laboratory of Advanced Biotechnologies for Health Assessments (LAB‐HA) Department of Electrical Engineering and Computer Science Lassonde School of Engineering York University Toronto M3J 1P3 Canada; ^4^ Department of Ecosystem and Public Health Faculty of Veterinary Medicine University of Calgary Calgary AB T2N 1N4 Canada; ^5^ Biologically Inspired Sensors and Actuators (BioSA) Department of Electrical Engineering and Computer Science Lassonde School of Engineering York University Toronto M3J 1P3 Canada; ^6^ Department of Physiology and Pharmacology University of Calgary Calgary AB T2N 1N4 Canada; ^7^ International Microbiome Centre Cumming School of Medicine Health Sciences Centre University of Calgary Calgary AB T2N 1N4 Canada; ^8^ Alberta Public Health Laboratory Alberta Precision Laboratories 3330 Hospital Drive Calgary AB T2N 4W4 Canada; ^9^ Department of Pathology and Laboratory Medicine Faculty of Medicine University of Calgary Calgary AB T2N 1N4 Canada; ^10^ Biomedical Engineering Graduate Program University of Calgary Calgary AB T2N 1N4 Canada

**Keywords:** clinical investigation, electrochemical nanobiosensors, graphene nanosheets, point‐of‐care diagnostics

## Abstract

The widespread accessibility of commercial/clinically‐viable electrochemical diagnostic systems for rapid quantification of viral proteins demands translational/preclinical investigations. Here, Covid‐Sense (CoVSense) antigen testing platform; an all‐in‐one electrochemical nano‐immunosensor for sample‐to‐result, self‐validated, and accurate quantification of the severe acute respiratory syndrome coronavirus 2 (SARS‐CoV‐2) nucleocapsid (N)‐proteins in clinical examinations is developed. The platform's sensing strips benefit from a highly‐sensitive, nanostructured surface, created through the incorporation of carboxyl‐functionalized graphene nanosheets, and poly(3,4‐ethylenedioxythiophene) polystyrene sulfonate (PEDOT:PSS) conductive polymers, enhancing the overall conductivity of the system. The nanoengineered surface chemistry allows for compatible direct assembly of bioreceptor molecules. CoVSense offers an inexpensive (<$2 kit) and fast/digital response (<10 min), measured using a customized hand‐held reader (<$25), enabling data‐driven outbreak management. The sensor shows 95% clinical sensitivity and 100% specificity (Ct<25), and overall sensitivity of 91% for combined symptomatic/asymptomatic cohort with wildtype SARS‐CoV‐2 or B.1.1.7 variant (*N* = 105, nasal/throat samples). The sensor correlates the N‐protein levels to viral load, detecting high Ct values of ≈35, with no sample preparation steps, while outperforming the commercial rapid antigen tests. The current translational technology fills the gap in the workflow of rapid, point‐of‐care, and accurate diagnosis of COVID‐19.

## Graphical abstract

Covid‐Sense (CoVSense) utilizes electrochemical immunosensing technology for rapid and quantitative detection of severe acute respiratory syndrome coronavirus 2 (SARS‐CoV‐2) viruses in clinical nasal specimens obtained from all‐inclusive cohort of patients, offering favorable features in terms of accuracy, signal correlation with viral load, and digital data management, compared to gold standard techniques, such as polymerase chain reaction and lateral flow strips.

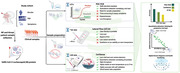

## 1 Introduction

The expeditious spread of severe acute respiratory syndrome coronavirus 2 (SARS‐CoV‐2) with its high infectivity and mortality rates resulted in a global predominance of the coronavirus disease 2019 (COVID‐19) pandemic.^[^ ^1^ ^]^ The SARS‐CoV‐2 virus is detectable in different specimen types mostly within 3–4 week postinfection.^[^ ^2^ ^]^ Individuals are usually symptomatic within 10 days of exposure but can have detectable virus 1–2 days before symptom onset. Individuals can even be contagious if they do not develop symptoms.^[^ ^3^ ^]^ More importantly, novel treatments for COVID‐19 reduce morbidity/mortality and hospitalizations but require taking medications within a specific period from symptom onset.^[^ ^4^, ^5^ ^]^ It is crucial to address the need for an easy‐to‐administer rapid diagnostic solution to control virus spread (isolation), administer effective treatments, and swiftly management of outbreaks.^[^ ^6^, ^7^, ^8^, ^9^ ^]^

The gold standard method for accurate diagnosis of COVID‐19 is relied upon the detection of viral RNA, mainly in respiratory biospecimens, using the real‐time reverse transcriptase‐polymerase chain reaction (RT‐PCR) technique.^[^ ^10^, ^11^ ^]^ Commercial and laboratory‐developed RT‐PCR assays have been used worldwide.^[^ ^12^ ^]^ Automated robotic platforms have substantially contributed to scaling up their performance in laboratory settings.^[^ ^13^ ^]^ However, this testing requires collection containers, transport to the lab (often with specific biosafety requirements), complicated sample preparation, and laboratory analysis, resulting in long turnaround times, and is expensive. Point‐of‐care RT‐PCR kits to some extent overcome these limitations.^[^ ^14^, ^15^ ^]^ However, the need for expensive instrumentation, instrument footprint, high cost per test, and space for the equipment make RT‐PCR impractical for widespread implementation of accurate point‐of‐care testing (POCT).^[^ ^16^ ^]^

While modified approaches such as recombinase polymerase amplification (RPA)^[^ ^17^ ^]^ and loop‐mediated isothermal amplification (LAMP)^[^ ^18^, ^19^, ^20^ ^]^ overcome some technological drawbacks like thermal cycling, their amplification strategies are still combined with optical detection methods,^[^ ^21^, ^22^ ^]^ necessitating sensitive and relatively expensive instruments and susceptibility to photobleaching^[^ ^23^ ^]^ or false‐positive results,^[^ ^24^ ^]^ and for those trackable by the naked eye, the readout accuracy is subject to errors. Many isothermal amplification methods have shown clinical sensitivity less than RT‐PCR even for symptomatic patients which makes them less than ideal candidates for large‐scale use.^[^ ^25^ ^]^

Viral structural proteins such as nucleocapsid (N)‐protein provide an alternative to viral RNA for POCT solutions without the need for amplification of the analytical target.^[^ ^26^, ^27^, ^28^, ^29^, ^30^, ^31^ ^]^ N‐proteins are the most abundant SARS‐CoV‐2 structural protein with no homology to human proteins, and resistance to significant mutational changes compared to spike (S)‐protein,^[^ ^32^ ^]^ making them an ideal biomarker for diagnosis of SARS‐CoV‐2. N‐proteins are enclosed within the virus's lipid membrane of the virus and are abundantly present in infected biospecimens whether in unbound form or within the structure of the virus, which could be the basis of rapid SARS‐CoV‐2 diagnosis.^[^ ^33^ ^]^ If a correlation is observed between the protein concentration levels and the viral loads, quantifying the rapid tests can also contribute to predicting the severity of the disease, for enhanced preparedness toward the selection of the most suitable treatments by the physician.^[^ ^34^, ^35^ ^]^

There has been extensive progress in commercializing rapid N‐protein antigen testing kits that are relatively inexpensive and give results in about 15 min.^[^ ^36^ ^]^ N‐protein qualitative lateral flow testing kits are being extensively used worldwide for rapid testing or screening for SARS‐CoV‐2.^[^ ^37^, ^38^ ^]^ Some examples of the hundreds of regulatory‐approved commercial kits include Abbott Panbio, Sofia Quidel, and BD veritor. Most do not require an instrument to read the test and have been approved mostly for symptomatic patients. Although these tests can be performed by the patients at home with no or minimal need for sample preparation, and are instrument‐free to interpret, they have lower sensitivity compared to RT‐PCR, especially for samples with low viral loads for those in the early stage of the infection or those who are asymptomatic.^[^ ^39^, ^40^ ^]^ Negative antigen POCT results are usually treated as presumptive, therefore they do not rule out either of the viruses and should not be employed as the only means of diagnosis or treatment/management of patients.^[^ ^41^ ^]^ Also, owing to the qualitative nature of these assays, quantitative levels of the virus cannot be determined to assist in estimating infectiousness, pathogenicity, and clinical management.^[^ ^42^ ^]^

There are several technologies that depict higher sensitivity than the conventionally used lateral flow assays for the detection of SARS‐CoV‐2 N‐protein,^[^ ^43^ ^]^ such as quantitative lateral flow assays,^[^ ^44^ ^]^ antibody‐ and aptamer‐based ELISAs,^[^ ^45^ ^]^ Surface Plasmon Resonance (SPR) immunosensing,^[^ ^46^ ^]^ nanoenzyme linked immunochromatographic sensors,^[^ ^47^ ^]^ chemiluminescence enzyme immunoassay method (CL‐ELISA) assays,^[^ ^48^ ^]^ single‐molecule array ELISA (Simoa) assays,^[^ ^49^ ^]^ quantum dots‐conjugated aptamer‐based N‐protein immunosensors,^[^ ^50^ ^]^ carbon nanofiber‐modified electrochemical N‐protein immunosensors,^[^ ^51^ ^]^ and plasmonic fiberoptic absorbance N‐protein immunosensors^[^ ^52^ ^]^ or oligonucleotide capped nanoparticles.^[^ ^53^ ^]^ These assays do not fulfill the void for a test that is rapid, portable, low‐cost, and capable of early diagnosis of SARS‐CoV‐2 and its variants. The most sensitive N‐protein assay, Simoa, has proven the potential value of N‐protein biomarker for rapid detection of SARS‐CoV‐2 within 2–3 weeks postinfection and shown its diagnostic equivalency to viral RNAs for detecting symptomatic and asymptomatic patients.^[^ ^54^ ^]^ However, Simoa involves expensive equipment and multiple liquid handling steps, limiting its utility for large‐scale and POCT. Only those immunosensor‐based assays may have potential for true rapid antigen‐based diagnostics.^[^ ^50^, ^55^ ^]^ Many SARS‐CoV‐2 antigen immunosensors, such as reliable electrochemical‐based systems,^[^ ^56^, ^57^ ^]^ could not yet reach the sensitivity and limit of detection comparable to Simoa. Those that reached such high performance have not been assessed in a clinical evaluation with a large number of symptomatic and asymptomatic participants.^[^ ^58^ ^]^ Also, their performance for detecting positive samples with low viral load has rarely been examined.

**Figure 1:**
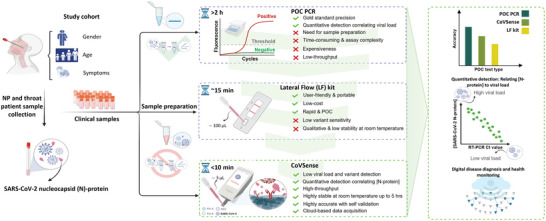
Schematic representation of the CoVSense specifications compared to point‐of‐care (POC) PCR and rapid lateral flow (LF) kits. The CoVSense platform was tested using nasopharyngeal (NP) and throat swab samples of patients of different genders, ages, and symptomatic/asymptomatic conditions. The clinical samples underwent pretreatment (RNA extraction) for PCR testing and were used in an untreated format for testing using LF kits and CoVSense. CoVSense performance in terms of sensitivity, specificity, quantitative detection for correlation of N‐protein concentration with the viral load, and digital functions such as cloud‐based data storage was highlighted.

In the present study, we report the development, detailed characterization, rigorous assay optimization, and clinical evaluation of Covid‐Sense (CoVSense) as an electrochemical SARS‐CoV‐2 N‐protein antigen nano‐immunosensor combined with a new low‐cost and noise‐less potentiostat reader. It rapidly quantifies N‐proteins in swab samples of infected patients, based on a novel electrode design, immunosensing protocol, and a reader. To further highlight the characteristics of the CoVSense platform, an illustration of sample collection and specifications of the clinical cohort for CoVSense were compared to PoC PCR and rapid lateral flow (LF) testing (**Figure** **1**). The immunosensing assay involves label‐free capture of N‐protein antigens with selective anti‐N‐protein antibodies immobilized on ready‐to‐use immunoaffinity potent strips, wherein the electrical signals were subsequently detected by a low‐cost impedance‐based potentiostat reader. This expedited accurate detection is made possible through the integration of highly functionalized graphene nanosheets, as a noble nanomaterial for sensing applications,^[^ ^59^ ^]^ embedded in a base ink containing conductive polymers intermixed in carbon paste. The immunosensor is rapid (<10 min), disposable, ultralow‐cost (<$2), and quantitative. The system is operated digitally, hand‐held, and low power. The immunosensing process does not need any sample preprocessing step such as cell lysis or centrifuge. The assay could detect as low as 421 fg mL^−1^ N‐protein antigen in spiked samples in buffer saline. CoVSense could detect SARS‐CoV‐2 wildtype and B.1.1.7 variant with 91% clinical sensitivity and 100% specificity in swab samples of SARS‐CoV‐2 positive patients in 5 days of the positive RT‐PCR test for combined symptomatic and asymptomatic patients. CoVSense performed comparably to RT‐PCR in detecting clinical samples with low viral loads. The kits outperformed other commercially available rapid antigen testing kits (here Abbott Panbio) in terms of detecting low viral loads, which are commonly seen in asymptomatic patients. Our stability assessments confirmed that the CoVSense's kit could reliably quantify viral loads in swab samples within at least 5 h of maintaining the strips at room temperature, fulfilling the shelf‐life requirement for most test settings. This technology can fill the large gap in the workflow of rapid and accurate COVID‐19 nanodiagnosis and contribute to contain viral infections from spreading in future pandemics, thanks to its notable commercial potential.^[^ ^60^, ^61^ ^]^

## 2 Results

### 2.1 CoVSense Functionality Assessment and Characterization

In an effort to realize the potential of quantifiable, near‐patient, and rapid antigen testing in a throughput manner, CoVSense, an integrated electrochemical sensing platform, was developed. It consists of a sensing strip equipped with bio‐ready technology, along with a compatible handheld impedance‐based readout system. The platform enables rapid, ultrasensitive, and reliable detection of SARS‐CoV‐2 N‐protein in swab samples, and is suited for large‐scale applications, e.g., clinical studies, due to its considerable scalability and reproducibility. The specific characteristics of the immunosensing interface, i.e., the electrodes, are being achieved through embedding nanostructured components within the ink matrix, to expedite bioanalyte conjugation, in a reproducible manner. Through intermixing the graphene nanosheets and poly(3,4‐ethylenedioxythiophene) polystyrene sulfonate (PEDOT:PSS) conductive polymers into the base carbon ink material, with the tuned ratio of added materials, mixing protocol optimization, and addition of diacetone acrylamide (DAAM) binders for increased ink cohesion and printability, a novel carboxyl‐rich surface is yielded for direct immobilization of antibodies for fabrication of the immunosensor, eliminating all conventional sensor preparation steps. The combination of the graphene nanostructures and the PEDOT:PSS conductive polymers created a compatible sensing surface, for the deposition of bioanalytes in various matrixes, including the universal transport media (UTM), without depicting any biofouling effects.

Graphene nanosheets, dispersed in the PEDOT:PSS ink, are the major elements rendering functional moieties needed for antibody immobilization, therefore they directly influence the sensitivity of the sensor. Also, biofouling on the electrode surface could be suppressed when the interaction of the incoming sample with other components of the ink is minimized, knowing that graphene does not introduce such interactivities. Carefully engineering the electrode surface to contain a higher number of graphene nanosheets is expected to result in higher conductivity of the electrode, increase the sensitivity of the sensor, and importantly impede biofouling. Hence, in our newly developed fabrication method for making the “bio‐ready” strips, it was essential to ascertain that all the layers of the graphene are highly delaminated from one another before proceeding to the intermixing step. We used ultrasonic coupled with heating (100 °C, less than the evaporation temperature of the solvent) for the exfoliation of these graphene sheets, while gradually adding DAAM binder to the primary ink starting at minute 15th to ensure that the graphene nanosheet layers are delaminated from one‐another, making all their valuable functional groups accessible for the next sensor preparation steps (Figure S1A, Supporting Information). This primary ink was then mixed with the secondary carbon paste ink and printed on its substrate. The printed strips were then heated up immediately to 150 °C (the evaporation temperature of the Dimethylformamide (DMF)) using an Infrared (IR) oven and maintained at this temperature for 5 min to create a surface rich with exfoliated graphene nanosheets. The printed ink was then immediately cooled down to the external fan temperature (≈15 °C) which resulted in a graphene‐rich bioready surface potent for direct immobilization of biocapture molecules (Figure S1B, Supporting Information). The IR oven, compared to the air‐based heaters uses electromagnetic radiation to penetrate the bulk of the matter, evaporating the DMF from inside the bulk of the printed ink and transporting it up to the surface. Therefore, this process contributes to the movement of the graphene sheets to the surface of the electrodes as their desired location. The enriched carboxyl functional groups on the surface of the electrode create a stable bond and eliminate the conventional need for multistep immunosensor fabrication, leading to higher reproducibility and reliability of electrochemical sensing and an enhanced sensing process not been practiced before. The electrochemical characterizations depicted higher conductivities for these sets of printed electrodes, confirming that the surface contains a higher number of transparent fully exfoliated graphene nanosheets, and these nanosheets on the surface are positioned near each other (the field emission scanning electron microscopy (FESEM) image in Figure S1C, Supporting Information).

This specific custom‐made ink allows for reproducible screen printing of electrochemical sensors and enables noiseless multiplex sensing with any intended sensor design that consists of two or more working electrodes. This ink optimization contributed to achieving the best possible results in terms of concentration of the functional groups, compactness of the electrode obtained by printing a small feature size, and sensitivity of the nano‐immunosensor.^[^ ^62^ ^]^ The working electrodes (WE) of the nano‐immunosensor inherently contain carboxyl functional groups (COOH), as a result of the abundant existence of the nanosheets of reduced graphene oxide in the ink, which allow for direct immobilization of monoclonal N‐protein antibody on the electrode. Owing to the composition of the printing ink containing functional groups, through designing an engineered nanochemistry for the ink material, no additional crosslinker, or intermediate functionalization step was needed to convert the bare electrode to an immunosensor, making it a fast‐reaching method for developing POC immunosensors to meet the clinical needs.^[^ ^63^ ^]^ The highly porous surface of the electrodes, which is favorably developed via integration of the conductive polymers of PEDOT:PSS, as well as the additional functional surface area of the graphene nanosheets effectively represents an increased area of sensing interface which exceptionally assists in yielding an ultrasensitive nanotechnology‐enabled system. The N‐protein immobilized WEs was blocked using bovine serum albumin (BSA) to restrict the unspecific binding of analytes except for the antigen of interest. BSA is an exceptionally inert protein with a high density of superficial lysine residues that prevents further binding of amino groups onto the immunosensor's surface. Details of the protocol for creating the CoVSense immunosensor including screen‐printed electrodes and the reader setup are presented in the Experimental Section.

**Figure 2:**
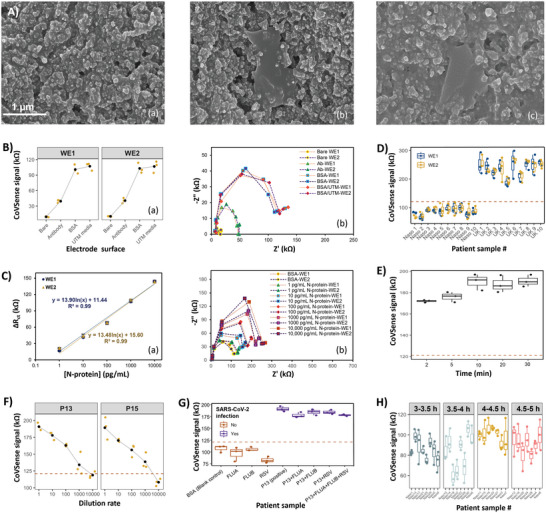
The characterization and performance evaluation of Covid‐Sense (CoVSense) nucleocapsid (N)‐protein immunosensor for clinical diagnosis of SARS‐CoV‐2. A) Field emission scanning electron microscopy (EFSEM) for a) pure carbon electrode, b) Graphene@PEDOT:PSS‐Carbon (GPePC) screen‐printed electrode with observable graphene nanosheet, and c) antibody immobilized nano‐immunosensor. B) Electrochemical impedance spectroscopy (EIS) signals recorded by the working electrode‐1 (WE‐1) and working electrode‐2 (WE‐2) of CoVSense for different surface modification steps, including antibody immobilization, bovine serum albumin (BSA) passivation, and incubation with the universal transport media (UTM) matrix, with a) representing the charge transfer resistance (*R*_ct_) measurements, and b) associated Nyquist diagrams. C) The EIS evaluation of the immunosensor's response to various concentrations of N‐proteins spiked in phosphate buffer saline (PBS) (1, 10, 100, 1000, and 10 000 pg mL^−1^) prepared via a dilatation test, resulting in (a) calibration curve for quantification of N‐proteins, based on the obtained b) Nyquist plots. D) Sensor's feasibility study with 20 clinical NP swab samples of 10 controls and 10 B.1.1.7 variant positive (+ve) patients measured using CoVSense on both working electrodes. E) Optimal incubation period of the clinical samples on the sensor. F) Samples #13 and #15 diluted up to 1000X were reliably detected by CoVSense where these samples were diluted between 1X‐10 000X and measured for N‐proteins. G) Validating the selective response of CoVSense recorded when the sensor was incubated with samples containing Influenza A (Flu‐A), Influenza B (Flu‐B), respiratory syncytial virus (RSV), and SARS‐CoV‐2 positive (patient sample #13), and their mixture with sample #13. H) CoVSense remains stable when 30 clinical controls were measured after their exposure to the ambient condition within 5 h after assay opening in the room temperature. All measurements were performed in triplicates (*n* = 3). The data points shown are mean ± standard deviation error.

The sensing strips present unique chemical features enabling a ready‐to‐immobilize approach for electrochemical nano‐immunosensor development. Physical characterization using atomic force microscopy (AFM) and FESEM confirmed the conversion of GPepC to an immunosensor following the immobilization of the antibodies (**Figure** **2**A; and Figure S2, Supporting Information). The AFM results depict that the addition of nanomaterials to the carbon ink increased the root‐mean‐square (RMS) surface roughness from 119 to 201 nm. It confirms that the GPePC electrode presents a surface with an appropriate surface roughness suitable for antibody deposition (Figure 2A,B). This shows that the carefully engineered chemistry of the ink, and the integration of graphene nanosheets compatibly intermixed with the PEDOT:PSS, results in increased nanofeatures, enhancing the surface area. The direct assembly of the antibodies on the surface is confirmed by the decrease in RMS roughness from 201 to 164 nm. It implies that the surface is smoother in the presence of antibodies as these proteins cover the surface projections including hills and valleys (Figure S2C, Supporting Information). These projections are specifically observable in the FESEM images of the pure carbon electrodes (Figure 2A(a)). For the GPePC sensor, the addition of graphene nanosheets is distinctly seen as transparent clothlike structures distributed throughout the surface (Figure 2A(b)). The antibodies were uniformly immobilized on the electrode providing a high‐affinity conjugation between the antibodies and graphene‐mixed Carbon@PEDOT:PSS (Figure 2A(c)), where this additional monolayer of antibody covers all the surface projections, which is relatively more observable in valleys than hills. The nanostructured interface enabled stable covalent assembly of the antibody on the electrode as the most critical step in immunosensor development.

For enhanced signal acquisition, a new high‐precision, programmable, and autocalibrated Bi‐Potentiostat circuit design was customized, representing a distinguishable noise cancellation system in three levels. Implementing a low pass filter with a programable cut‐off frequency in the hardware allows for eliminating noises in frequencies that are more than the impedance measurement frequency. Also, a similar low‐pass filter was utilized in the microcontroller's firmware at the data processing level. The output averaging for multiple consecutive measurements also introduced another level for noise reduction, and hence more accurate signal reading. The specifications of the CoVSense readout for signal recording from our dual immunosensor enabled rapid, noise‐free, repeatable, and at the same time, accurate obtainment of electrochemical impedance spectroscopy (EIS) signals from the CoVSense. The Bi‐Potentiostat is able to transmit the acquired digital data to a computer or a smartphone via communication protocols based on wired or wireless means, such as universal serial bus (USB) or Bluetooth technique, respectively. The recorded impedance changes with respect to the target bioanalyte concentration. Without compromising the accuracy and through an optimization process, the entire desired impedance range is covered using 7 logarithmically spaced sampling points over the frequency spectrum of 1400 to 1 Hz (customized for reliable N‐protein evaluation here) for each of the WEs. The detail of the potentiostat specifications and the comparative performance to standard potentiostats are reported in the Experimental Section as well as Supporting Information and Supporting File 1. The read‐out system was analyzed with known impedance values and redox solutions prior to clinical tests. The detail of the design is found in the Supporting Information. The reader was made from a commercially available microcontroller and out‐of‐shelf components and offers the cheapest yet accurate and reliable time‐domain impedance analyzer. The total cost of the custom‐made potentiostat read‐out system is less than $25 which can be further decreased in the mass production phase.

The reader provides features for digital test management and telecommunication with electronic health records (EHRs) while preserving its user‐friendliness for patient use. First, the user logs into the device using a predefined user identification (ID) (Figure S3A, Supporting Information). The query sent to the server is authenticated using the device's user ID and registered MAC address. Next, the user initiates a new test and can also select other pages, such as history or configurations, through the main page (Figure S3B, Supporting Information). There exists the option of answering questionnaires about demographics and symptoms (Figure S3C, Supporting Information). Upon inserting the sensor, the device starts collecting signals by pushing the “Run” button, and the results show up in less than 2 min. The recorded data are fed into the model to obtain the charge transfer resistance (*R*_ct_) using the fitted curve of the Nyquist plot, with the Real (Z′) versus Imaginary (Z″) axis in kilo‐ohm (kΩ). A high‐frequency intercept is fitted to the semicircle on the real axis of the Nyquist plot, determining the *R*_ct_ at the diameter intersection. Using the predefined calibration curve of *R*_ct_ value versus N‐protein concentration, the algorithm quantifies N‐proteins in the sample, and based on the cut‐off value calculated through the clinical feasibility study, pops up the positive or negative results (Figure S3D, Supporting Information). The raw data and questionnaires are saved locally on the device's internal memory or in the Cloud for future analysis, if needed. The optional internet connection also allows the administrator for updating the device's internal algorithms.

The surface of WEs was examined electrochemically to characterize the impedance signals in response to surface modifications. We selected the EIS method in this work, owing to its higher sensitivity compared to other electrochemical techniques, and its benefit in providing data about the electrochemical behavior of the surface.^[^ ^64^ ^]^ The unmodified GPePC WE showed the lowest impedance recorded among all surfaces tested in this work, while its impedance increased upon further surface modification. Unmodified GPePC WE offered the impedance of 9.13 ± 0.40 kΩ (relative standard deviation (RSD) 7.3%), while the impedance increased to 39.41 ± 0.4 kΩ (RSD 7.6%) for the N‐protein antibody‐coated WE, 100.90 ± 5.1 kΩ (RSD 8.7%) after adding BSA, and finally 107.1 ± 4.3 kΩ (RSD 7%) in the presence of UTM on the immunosensor (Figure 2B(a,b). The UTM buffer was dispensed on the prepared immunosensor for investigating possible matrix effects in case the samples are stored in buffers other than phosphate‐buffered saline (PBS), which is normally used for spike‐sample preparation or clinical sample storage matrix. Although the pH of UTM media and PBS are the same (7.3–7.4), it is essential to test the performance of the immunosensor incubated with UTM to ensure that its biological components do not adversely affect the electrode surface. This is perceived by comparing the *R*_ct_ signals of the BSA‐coated electrodes incubated with UTM media for 30 min and unincubated ones, showing no significant difference (100.9 ± 8.81 and 107.10 ± 7.46 kΩ, respectively, *p* = 0.712). Therefore, the nasopharyngeal (NP) or throat samples collected from patients in UTM would not affect the CoVSense's electrode performance. The electrochemical characterization, calibration response in PBS, and immunosensor results when incubated with UTM, revealed that CoVSense's architecture did not foul, and as a result does not possibly obstruct N‐proteins from being detected on WEs, confirming the universality and matrix agnostic response of GPePC WEs.

### 2.2 Sensor Efficiency Preassessment in Nonclinical Samples

The electrochemical measurements, performed using 2.5 mm of [Fe(CN)_6_]^3−/4−^ as the redox probe, indicated that the impedance of the WE increased significantly in N‐protein spiked samples. In other words, the immunoreaction of N‐proteins with the immobilized antibodies increased *R*_ct_ measured using EIS. The performance of the CoVSense immunosensor for quantification of N‐protein concentrations was evaluated by drop‐casting various PBS‐spiked N‐protein concentrations samples on the immunosensor, followed by the steps of washing, redox probe adding, and detection. Upon EIS signal acquisition from immunosensors, a sensitive sigmoidal response was observed, through which a linear operational range was derived. The response was *y* = 13.90ln(x) + 11.44, *R* ^2^ = 0.99 and *y* = 13.48ln(x) + 15.60, *R* ^2^ = 0.99 for N‐protein spiked in PBS for WE1 and WE2, respectively. CoVSense offers a linear detection range of 1 pg mL^−1^ to 10 ng mL^−1^ (Figure 2C(a,b)), with a limit of detection (LoD) of 421 and 410 fg mL^−1^, and sensitivity of 5037 and 4883 Ω mL pg mm^−2^, for detecting N‐proteins by WE1 and WE2, respectively. From the response curve, it is evident that the CoVSense's response is similar for both working electrodes (Figure 2B), showing that the detection is performed reliably and each of the two working electrodes can be used interchangeably.

Further characterization tests were conducted to examine the reliability of the recognition element and ensure that the signals obtained are exclusively associated with the interaction of N‐protein immobilized antibodies and the incoming proteins in the samples. The bio‐ready strips were immobilized with the anti‐SARS‐CoV‐2 S‐protein antibodies and tested against N‐protein spiked samples (spiked in PBS) with two different N‐protein concentrations of 10 and 1000 pg mL^−1^. The results corroborated no significant interaction between the nonspecific antibodies and the N‐protein antigens (Figure S4A, Supporting Information). In another set of experiments, the antibody‐unmodified electrodes (here are the bare electrodes) were incubated with N‐protein‐containing samples (100 pg mL^−1^ N‐protein spiked in PBS). In the absence of target antibodies on the electrode, no significant change in signal was detected as a result of the interaction between the bare electrode and N‐proteins. The specific response of the selected biorecognition element (here anti‐SARS‐CoV‐2 nucleocapsid antibody) to the SARS‐CoV‐2 N‐protein antigen has also been confirmed in other clinical studies.^[^ ^65^, ^66^, ^67^ ^]^

### 2.3 Clinical Feasibility Investigation for Detecting SARS‐CoV‐2 in NP or Throat Swab Samples Using CoVSense

To examine the feasibility of measuring N‐protein levels in clinical samples using the refined CoVSense prior to proceeding to large cohort testing, 10 clinical controls (negative samples) and 10 positive swab samples of the SARS‐CoV‐2 variant of focus (B.1.1.7 lineage) were tested on the immunosensor. All 10 control samples offered a mean *R*_ct_ of 86.40 ± 3.14 kΩ (RSD 11.48%; *R*_ct_ range: 64.8–100 kΩ), close to that recorded after the BSA blocking step (100.90 ± 8.81 kΩ (RSD 8.7%)). In contrast, 10 clinical (B.1.1.7) variant positive (+ve) samples offered a mean *R*_ct_ of 237.20 ± 6.76 kΩ (RSD 9.0%; *R*_ct_ range: 186.49–257.95 kΩ) (Figure 2D). Based on the validated RT‐PCR results, the electrochemical immunosensor could reliably differentiate between positive and negative samples, confirming the clinical feasibility of the CoVSense system.

### 2.4 CoVSense's Self‐Validation Feature for Detecting N‐Proteins

Electrochemical immunosensors can benefit from a self‐validation unit to suppress the matrix effect on sensing signals without compromising their low‐cost value, while also eliminating the need for sample preparation.^[^ ^68^ ^]^ Here we use unprocessed clinical samples for clinical testing of the CoVSense immunosensor. With the observation of cellular clumps and intra‐ and inter‐variation in viscosity of clinical swab samples, the matrix effect may not be trivial on our immunosensor. Therefore, the self‐validation system in the present work is on the basis of the incorporation of a second electrode which acts as a control for reducing the risk of false‐positive detection. A two‐working electrode configuration of the CoVSense containing two adjacent N‐protein immunosensors along with the low‐cost and noise‐free bi‐potentiostat was used to evaluate the self‐validation performance of CoVSense. Testing of 20 clinical samples (10 each B.1.1.7 variant positive and 10 negative controls) on dual N‐protein immunosensors showed insignificant deviation in the response of WEs (Figure 2D). The ability of both WEs to successfully detect positive patients presented comparable *R*_ct_ values determined by the paired WEs, showing the negligible role of sample preparation on our sensor response and confirming the self‐validation of the testing kit. Regardless, the effect of potential variations due to matrix or other environmental factors, if becoming effective, can be compensated with minimal cost using this dual sensing unit.

### 2.5 Assay Clinical Turn‐Around Time Optimization

The optimal incubation period for the interaction of N‐proteins and the nano‐immunosensor was investigated. Clinical positive sample #13 (SARS‐CoV‐2 wildtype) with the Ct value of 26.49 was tested for the optimization test. This sample was selected given its intermediate Ct value, not very high close to 36, and not very low, around 15, but still containing an intermediate load of viral particles.^[^ ^69^ ^]^ This sample was directly cast‐coated on the immunosensor without any prior sample preparation step. Incubation was performed for 2, 5, 10, 20, and 30 min. No significant difference was observed for the signals of 2 and 5 min incubation, with RSDs of 0.65% and 2.71%, respectively (Figure 2E). Moreover, for 10, 15, and 30 min incubation, *R*_ct_ was measured to be 190.32 ± 4.35 kΩ (RSD 3.96%), 187.78 ± 4.64 kΩ (RSD 4.28%), and 190.93 ± 3.20 kΩ (RSD 2.90%), respectively (Figure 2E), representing only 10% increase compared to 2 and 5 min incubation period. These data show that antibody/antigen immunoreaction is complete within 10 min of sample dropping on the nano‐immunosensor, verifying that the proposed nano‐immunosensor rapidly (<10 min) detects N‐proteins in an unprocessed NP or throat swab. The optimal incubation period for the interaction of N‐proteins and the immunosensor was investigated. Clinical positive sample #13 (SARS‐CoV‐2 wildtype) with the Ct value of 26.49 was tested for the optimization test.

### 2.6 Detection of Virus in Clinical Samples with Low Viral Loads

Swab samples collected by diagnostic labs and clinics are often preserved and transported in standard UTM (or similar collection media). It means that the sample tested is always diluted to the volume of the collection media in the swab‐preserving tube. To further assess the sensitivity of the nano‐immunosensor in detecting diluted samples, we performed sample dilution tests to identify the CoVSense's response in undiluted and 10X, 100X, 1000X, and 10 000X diluted samples. Two SARS‐CoV‐2 wildtype clinical samples with approximately similar Ct values labeled #13 (Ct value = 26.49) and #15 (Ct value = 26.33) were examined for this purpose. As for sample #13, the *R*_ct_ value for the undiluted sample was measured 190.93 ± 5.54 kΩ (RSD 2.90%), which decreased sequentially upon 10X dilution each time and reached 132.94 ± 18.51 kΩ (RSD 13.92%) for 1000X dilution, and 118.82 ± 4.94 kΩ (RSD 4.16%) for 10 000X dilution. The same trend was observed for sample #15 which finally provided 108.21 ± 5.35 kΩ (RSD 4.95%) for 10 000X dilution (Figure 2F). Based on the assay clinical feasibility study, the expected mean *R*_ct_ for immunosensors incubated with UTM is 107.1 ± 4.3 kΩ (RSD 7%), and hence the results obtained for 10 000X dilution are not significantly different from the signal of blank and thus are not quantifiable by our nano‐immunosensor. The results show that the sensor detects N‐proteins in undiluted (the sample collected in UTM media), 10X, 100X, and 1,000X diluted samples within a maximum RSD of 8.7% (Figure 2F). Further diluting the samples to 10 000X resulted in a drop in *R*_ct_ by more than 20%. The CoVSense's response could successfully detect the 1000X diluted samples, and this dilution limit remains within the detectable range of the sensor. Based on the common RT‐PCR standard curves, and considering an RT‐PCR efficiency of 100%, we expect to have an increase of around 3.3 in the Ct values of samples diluted 10X of their higher concentration. This dilution is equivalent to Ct = 35, based on the calculations of Ct value increase upon each 10X dilution.^[^ ^70^ ^]^

### 2.7 Validation of Clinical Specificity of CoVSense

COVID‐19 and other respiratory viral infections like influenza are classified as contagious respiratory diseases. Although they are caused by different viruses, their symptoms highly overlap, making it difficult to differentiate them only by relying on clinical symptoms. Hence, a SARS‐CoV‐2 diagnostic tool needs to be specific for COVID‐19, essential to reduce the risks of misdiagnosis. We examined the specificity of CoVSense for clinical detection of the SARS‐CoV‐2 virus compared to samples containing influenza A (Flu‐A), influenza B (Flu‐B), and respiratory syncytial virus (RSV) (Figure 2G). The clinical positive sample #13 was tested with CoVSense and offered *R*_ct_ = 190.93 ± 5.54 kΩ (RSD 2.90%). The *R*_ct_ signals for testing the samples containing Flu‐A, Flu‐B, and RSV were 96.69 ± 15.15 kΩ (RSD 15.66%, *p* = 0.712), 105.88 ± 4.23 kΩ (RSD 3.99%, *p* = 0.999), and 82.78 ± 6.81 kΩ (RSD 8.22%, *p* = 0.016), respectively, which are comparable to the mean signal of the SARS‐CoV‐2 negative samples (86.4 ± 3.14 kΩ (RSD 11.48%) (*p* values are provided for the comparison of the non‐SARS‐CoV‐2 viral infections and the negative sample). Next, 15 µL of clinical sample #13 was mixed with 15 µL of Flu‐A, Flu‐B, and RSV to have a total volume of 30 µL from each mixed sample, where *R*_ct_ signals were measured to be 177.66 ± 5.78 kΩ (RSD 3.25%, *p* = 0.429) for the mixed sample #13 and Flu‐A, 184.12 ± 6.34 kΩ (RSD 3.44%, *p* = 0.958) for the mixed sample #13 and Flu‐B, and 184.07 ± 4.63 kΩ (RSD 2.51%, *p* = 0.957) for the mixed sample #13 and RSV (*p* values are provided for the comparison of the mixed samples and the sample #13). Also, 5 µL of each of the three interference samples were mixed with 15 µL of sample #13, where *R*_ct_ was measured to be 177.74 ± 1.92 kΩ (RSD 1.08%). The *R*_ct_ signals measured for the samples containing only interferences were up to 50% less than that for the clinical positive sample #13 but nearly identical to that recorded for the UTM (Figure 2G). Despite the presence of interferences from the carrying media, the CoVSense specifically detected N‐proteins in positive samples.

### 2.8 Stability Assessment of the CoVSense Immunosensor

For rapid and POC testing, antibody‐based immunosensors are mostly preserved at 4 °C prior to use.^[^ ^71^ ^]^ They also need to be used within a specified period after opening the sensor as the surface chemistry and/or biorecognition molecules may lose their stability. To assess the thermo‐stability of CoVSense for use within 5 h after opening the immunosensor package, the clinical samples were divided into four test groups (Table S1, Supporting Information), each tested at 3.5, 4, 4.5, and 5 h after exposing the sensors to the ambient condition. The results show that the *R*_ct_ signals measured for all the clinical control samples ranged from 59.28 to 106.83 kΩ, measured at different time‐points, representing the *R*_ct_ signals recorded in the presence of UTM (Figure 2H; and Table S1, Supporting Information). Therefore, the surface chemistry of CoVSense did not foul upon exposure to ambient conditions, stemming from the robust binding of Graphene@PEDOT:PSS within the carbon molecules in the sensor material itself and the strong affinity of the antibody to the functionalized electrode.

### 2.9 Cohort Clinical Evaluation of CoVSense

Following the reliable and convincing initial feasibility studies for lab validation of CoVSense in detecting N‐proteins in the spiked and small number of clinical samples, the immunosensor was used for detecting SARS‐CoV‐2 in a clinical cohort tested with RT‐PCR. In total, 105 clinical samples were examined in a three‐way blind study, out of which 40 were clinical controls (including samples collected from patients with other viral respiratory infections), 30 positive samples (wildtype SARS‐CoV‐2, all nasopharyngeal swab samples), and 35 positive samples (SARS‐CoV‐2 B.1.1.7 variant samples: 25 throat swab samples, 9 NP swab samples, and one unknown origin). The details of the clinical sample handling, CoVSense immunosensor preparation, and EIS measurements are provided in the Experimental Section. The characteristics information of the patients is presented in Table S2 (Supporting Information). RT‐PCR's amplification cycle threshold (Ct) cut‐off value of 37 was set for differentiating SARS‐CoV‐2 positive and negative control patients.^[^ ^72^ ^]^ Most of the control samples were prepandemic control samples, from patients without any infection. Patients #36 and #37 had Flu A, while patients #38 and #39 had Flu B, and patient #40 was infected with RSV. Details of each measurement including Ct values and *R*_ct_ signals are given in Table S3a,b (Supporting Information). These measurements were considered benchmark results for the clinical performance assessment of CoVSense. The clinically diagnosed COVID‐19 cases had a mean Ct value of 24.35 ± 5.32 (range: 15.21–35.66). Clinical swab samples were assessed in triplicates.

**Figure 3:**
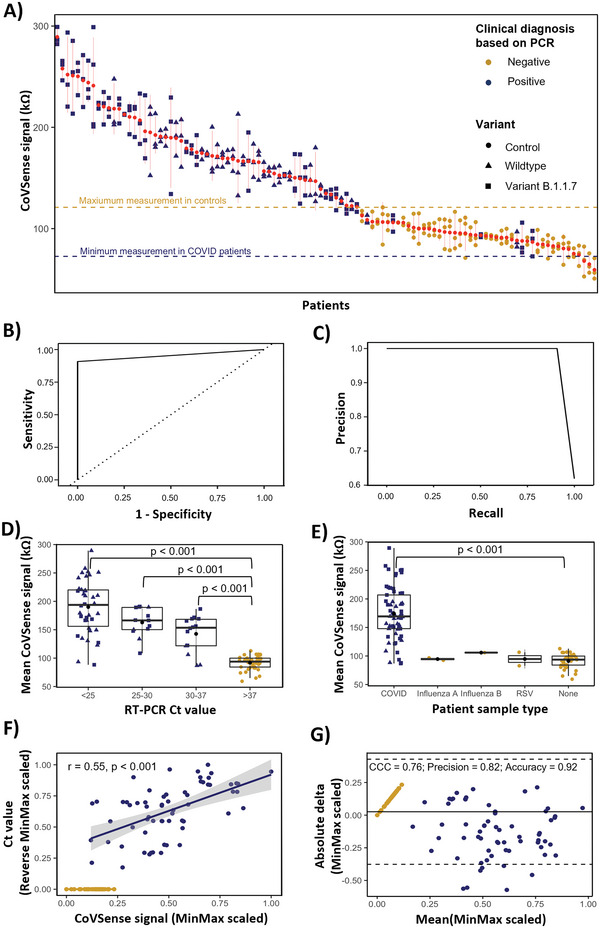
Clinical cohort measurements for infection detection with CoVSense and real‐time reverse transcription‐polymerase chain reaction (RT‐PCR). A) Impedance recorded for the clinical cohort of 105 patient samples: 65 SARS‐CoV‐2 positive samples and 40 negative controls. The measurements were done in triplicates. Red points denote the mean measurement per sample. B) sensitivity and specificity and C) precision and recall stratified by all patients with and without symptoms. D) Mean CoVSense impedance measurements in relation to RT‐PCR Ct value groups within the cohort. E) Mean CoVSense impedance measurements in SARS‐CoV‐2 patients compared to patients with other viral upper respiratory infections. F) Correlation of RT‐PCR Ct values and CoVSense impedance measurements. The measurements were MinMax scaled and assessed by Spearman rank correlation. RT‐PCR Ct values were reverse MinMax normalized whereby the lowest and highest Ct values were normalized to 1 and 0 (range: 15.21–35.66), respectively. G) Lin's concordance correlation coefficient (CCC), comparing the reliability and accuracy of CoVSense measurement compared to RT‐PCR. The measurements were MinMax scaled. The annotations of (A) are applicable to (D–G).

Overall, the majority of SARS‐CoV‐2 positive patients (59 out of 65 PCR positive samples) had higher CoVSense response signal on all three replicates (174.67 ± 26.14 kΩ, Range: 289.25–86.81 kΩ) compared to the controls (90.90 ± 6 kΩ, Range: 112.85–59.28 kΩ) (**Figure** **3**A). The threshold of CoVSense response for SARS‐CoV‐2 detection was optimized using Youden's index and determined to be 118.85 kΩ. Given that in real‐world settings, the diagnosis is made based on only one measurement, we estimated the cut‐off based on the mean and the minimum of the three measurements per patient which resulted in the same cut‐off threshold. We performed all further analyses based on the mean of the measurements per patient. This cut‐off was used to define CoVSense positive and negative patients. CoVSense showed clinical sensitivity of 95% and clinical specificity of 100% for samples with Ct <25. Also, CoVSense showed clinical sensitivity of 91% and clinical specificity of 100% for all clinical samples tested including the patients with and without symptoms (Figure 3B,C). We then compared the CoVSense performance in relation to the RT‐PCR Ct values and for infections with different viral pathogens (Figure 3D,E; and Figure S5, Supporting Information). The minimum CoVSense response showed comparable results to the mean of three CoVSense measurements. CoVSense response was higher for samples with Ct values of below 25 (189.96 ± 48.02 kΩ) and successively decreased with increasing Ct value category (Figure 3D). However, except for six samples (Patient IDs: 4, 18, UK15, UK17, UK23, and UK24), which are false‐negative cases, the rest had significantly higher CoVSense responses than the controls (*p* <0.001). CoVSense was also specific for SARS‐CoV‐2 infection (174.67 ± 26.14 kΩ) as samples from patients with other respiratory viruses had low CoVSense response which was comparable to the negative controls (Figure 3E).

### 2.10 Concordance of CoVSense with RT‐PCR

We found a significant correlation between normalized CoVSense (MinMax normalized) and RT‐PCR (reverse MinMax normalized) measurements in the SARS‐CoV‐2 infected samples (Spearman rank correlation coefficient = 0.55, *p* <0.001; Figure 3F), relating the Ct values of the positive samples with the impedance response obtained using CoVSense. It can be concluded that the CoVSense platform can reliably record higher impedance signals, attributed to the higher concentrations of N‐protein, representing higher viral loads.^[^ ^73^ ^]^ This can open avenues of opportunity for the interchangeable deployment of the presented platform compared to RT‐PCR, for the rapid quantitative detection of SARS‐CoV‐2 antigens. The strong comparability of CoVSense and RT‐PCR performance was further confirmed using Lin's concordance correlation coefficient (CCC). It allows for comparing the accuracy and reliability of CoVSense responses considering RT‐PCR as the gold standard, specifically for equivalently detecting the positive cases. We observed a strong CCC (0.76, 95% CI 0.68–0.82), a precision of 0.82, and overall measurement accuracy of 0.94 (Figure 3G). A bias correction factor of 0.94 was observed which indicates that the CoVSense responses minimally deviated from that of RT‐PCR.

### 2.11 CoVSense Assessment for Wildtype and Alpha Variant of SARS‐CoV‐2

**Figure 4:**
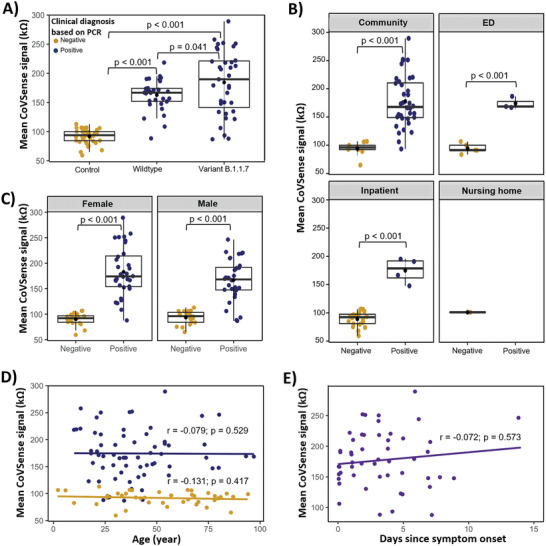
Evaluating the CoVSense's measurements for detecting SARS‐CoV‐2 and its variant in different settings. A) CoVSense measurements for wildtype and UK (B.1.1.7) variant compared to the controls. B) Field application of CoVSense in patients from the community, emergency department (ED), inpatients, and nursing homes. C) Gender‐based CoVSense's performance within the clinical cohort. D) CoVSense measurements are not impacted by patient age, assessed by Spearman rank correlation. E) CoVSense measurements are not impacted by the time since symptom onset, assessed by Spearman rank correlation. The annotations of (A) are applicable to (B–D).

We further analyzed the clinical performance of CoVSense for detecting wildtype SARS‐CoV‐2 versus the alpha/B.1.1.7 variant for the same clinical cohort. CoVSense responses were higher in COVID patients than in controls irrespective of the virus variant (**Figure** **4**A). Mean CoVSense response was higher in B.1.1.7 variant (184.40 ± 54.89 kΩ) compared to the wildtype (162.62 ± 27.05 kΩ; *p* = 0.041). However, the spread of the signals was also wider for the variant (SD = 54.89, range: 86.81–289.25 kΩ) compared to the wildtype (SD = 27.05, range: 88.30–218.41 kΩ; Figure 4A). Despite intra‐ and inter‐sample variation in the signals arising between wildtype and B.1.17 variants stemming from a wide variation in the Ct values, they were uniquely detected compared to the negative controls. Mean impedance from uninfected, healthy controls was 91.03 ± 6.9 kΩ, detected within the 59.3–112.85 kΩ range. All SARS‐CoV‐2 (*N* = 65) samples recording signals, confirmed through RT‐PCR, relative to negative samples from healthy controls (*N* = 40) prove the accurate assessment and detection of N‐protein as a surrogate COVID‐19 biomarker in biofluids. Moreover, regardless of sample type (NP or throat swab), CoVSense identified and quantified the presence of the COVID‐19 antigen biomarker, demonstrating its accurate and noninvasive capability to detect and diagnose SARS‐CoV‐2 infection.

### 2.12 CoVSense Assessment Based on Patients’ Characteristics and Demographic Variables

Every diagnostic platform should perform equally well for all patients regardless of the disease setting, gender, age, and clinical manifestation. We assessed the CoVSense signals in the cohort used in the clinical evaluation based on patient type community (emergency department (ED), inpatient, and long‐term care). We observed an equally well performance of CoVSense in COVID patients compared to controls in all assessed settings with a sufficient sample size (Figure 4B). Similarly, CoVSense indiscriminately performed equally well for male and female patients (Figure 4C) and the measurements were not impacted by patient age (Figure 4D) or days passed since disease onset (Figure 4E). Our results suggest that the CoVSense measurements are not impacted by patient characteristics. Our results indicate the consistency in the performance of CoVSense when testing the samples originated from varying genders or settings in the tested clinical cohort.

**Figure 5:**
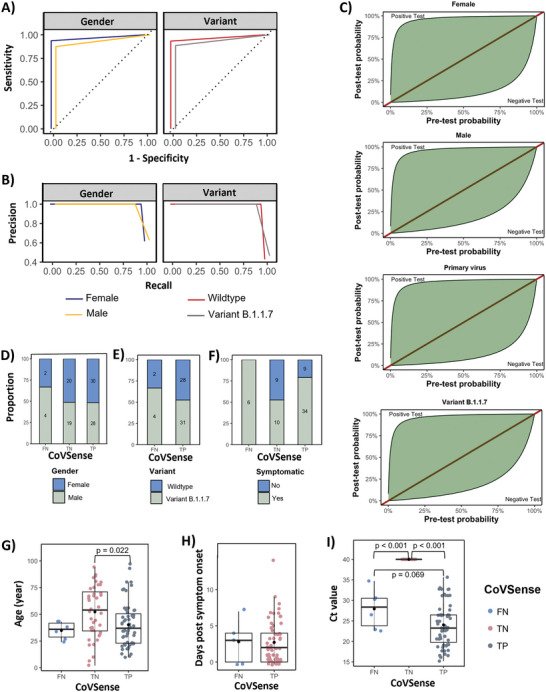
Performance of CoVSense in the detection of SARS‐CoV‐2 infection in comparison with the gold standard clinical approach using RT‐PCR. Receiver operation characteristic (ROC) curve representing. A) Sensitivity and specificity and B) precision and recall stratified by patient gender and viral variants. C) Conditional probabilities showing the diagnostic capability of the CoVSense in the clinical cohort according to the pretest probabilities of infection stratified by patient gender and viral variant. The distribution of CoVSense SARS‐CoV‐2 infection diagnosis results based on D) gender, E) virus variant, F) symptoms, G) age, H) days postsymptom onset, and I) Ct‐value. TN, TP, and FN are true negative, true positive, and false negative, respectively.

The CoVSense performance was compared to that of the RT‐PCR while considering demographic factors. CoVSense had high sensitivity, specificity, precision, and recall for SARS‐CoV‐2 detection for male (area under the curve (AUC) = 0.94, 95% CI: 0.88–0.99) and female AUC = 0.97, 95% CI: 0.93–0.99) patients as well as wildtype (AUC = 0.97, 95% CI: 0.92–0.99) and variant B.1.1.8 (AUC = 0.94, 95% CI: 0.89–0.99) viruses (**Figure** **5**A,B; and Figure S6, Supporting Information). Clinical diagnosis is made based on a combination of clinical manifestation plus laboratory assessment. In other words, some patients have a high probability of having COVID‐19 infection from the medical history and clinical examinations prior to a laboratory test. In this case, a highly sensitive and specific test might not add significantly to the clinical diagnosis. On the other hand, when the probability of COVID‐29 is low based on the clinical assessment, a highly accurate diagnostic method could provide valuable information for clinical decision‐making. We next assessed the CoVSense performance considering the pretest probability of COVID based on gender and viral variants (Figure 5C). Regardless of the gender or virus variants, a positive test is associated with a high probability of infection irrespective of the pretest probability. However, a negative test is associated with lower post‐test probability at higher pretest probabilities in female patients and wildtype infections. For example, at 75% pretest probability, a negative test is associated with 27% and 16% post‐test probability in male and female patients, respectively. Approximately 9% of the patients tested with CoVSense were not clinically diagnosed as COVID patients (Figure S7A,B, Supporting Information). From a diagnostic point of view, FN is more important resulting in falsely not diagnosing the SARS‐CoV‐2 infection. The virus variant B.1.1.7 had a considerably higher FN rate compared to the wildtype (11.4% vs 6.7%) (Figure S7C, Supporting Information). Acknowledging the small sample size for subgroup analysis, we examined patients’ characteristics based on the CoVSense‐based diagnosis of SARS‐CoV‐2. True‐positives (TP) and true‐negatives (TN) were detected equally well for male and female patients (Figure 5D) and the virus variants (Figure 5E). FNs were higher in male patients and variant B.1.1.7 (Figure 5D,E). CoVSense's TP detection rate was higher in symptomatic patients (79%), while TN detection was composed of both symptomatic and asymptomatic patients at a comparable proportion (Figure 5F). Intriguingly, all CoVSense FN cases were symptomatic patients (Figure 5F) and if this trend is dominant in larger cohort clinical examinations, retesting is then suggested for symptomatic individuals, who are mainly suggested to quarantine until the symptoms are resolved. Overall, the FN detection was observed in patients of younger age albeit not statistically significant (34.83 ± 8.04, *p* = 0.165) (Figure 5G–I).

### 2.13 CoVSense Diagnostic Performance in Comparison with Abbott Panbio

**Figure 6:**
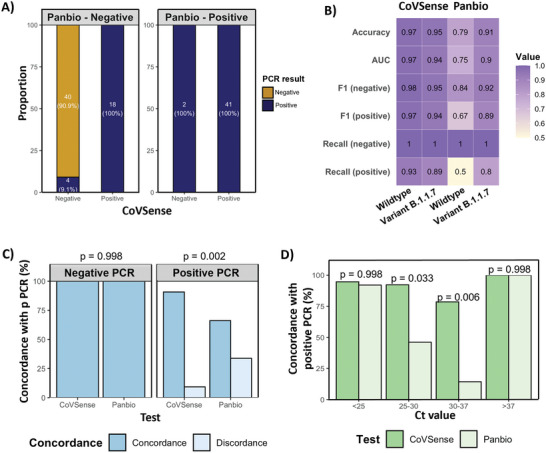
Performance of CoVSense in the detection of SARS‐CoV‐2 infection compared to COVID rapid diagnostics from Panbio. A) Concordance of CoVSense and Panbio SARS‐CoV‐2 diagnosis compared to the gold standard RT‐PCR. B) Diagnostic performance of CoVSense and Panbio stratified by the virus variant. C) Concordance of CoVSense and Panbio in relation to RT‐PCR results. D) Concordance of accurate diagnosis of SARS‐CoV‐2 infection by CoVSense and Panbio in relation to RT‐PCR Ct values.

We next assessed the performance of CoVSense with the commercially available Panbio rapid antigen tests, in our cohort of 65 PCR‐positive samples and 40 PCR‐negative samples. Overall, there was a good concordance between the CoVSense when Panbio was positive. In this group of 43 Panbio positive samples, all patients were RT‐PCR positive, whereas 41 (95.4%) patients were CoVSense positive, with only 2 patients (4.6%) being CoVSense negative suggesting a comparable performance with positive Panbio (**Figure** **6**A). However, CoVSense had a more accurate performance in Panbio negative cases. In this group, 22 (35.5%) samples were RT‐PCR positive; of which 18 (81.8%) were accurately diagnosed by CoVSense. Additionally, 40 patients were RT‐PCR negative; all of whom were correctly diagnosed negative by CoVSense. However, CoVSense detected 6 (9.1%) RT‐PCR positive patients as negative which is much fewer than the false‐negative rate of Panbio (40, 90.9%; Figure 6A). Detailed performance comparisons of CoVSense and Panbio for different virus variants are also presented in Figure 6B; and Table S4 (Supporting Information). CoVSense has high accuracy, AUC, F1 score, and recall for both wildtype and variant B.1.1.7. While Panbio performance is also high for variant B.1.1.7, the performance is not as good as CoVSense for the wildtype. Specifically, the F1 score and recall for positive cases are very low for the wildtype as assessed by Panbio (Figure 6B). Overall, both Panbio and CoVSense demonstrate high concordance with a negative RT‐PCR result. In contrast, CoVSense has higher concordance with positive RT‐PCR compared to Panbio (Figure 6C) with the most discordance occurring at Ct values of 25–37 (Figure 6D). Based on the data obtained from the clinical cohort of the present study, a sensitivity and specificity of 66% and 100%, respectively, were obtained for the Panbio kits, which is noticeably lower than the sensitivity reported for CoVSense (91%), therefore CoVSense depicts even higher sensitivity by enlargement of the clinical cohort tested.

## 3 Discussion

The addition of various conductive nanomaterials and nanocomposites to the surface of carbon or graphene electrodes has significantly enhanced the sensitivity of electrochemical immunosensors by 2–3 orders of magnitude higher than ELISAs for detecting protein analytes.^[^ ^74^ ^]^ However, scalable and reproducible production of such nano‐immunosensors is still a challenge due to the complex and multistep deposition of nanomaterials, the risk of the coffee‐ring effect, or the limited anchoring of nanomaterials to the substrate.^[^ ^75^ ^]^ Several strategies have been implemented to address these concerns, such as on‐chip coating of nanomaterials to eliminate manual steps and create a reproducible deposition process, one‐step nanomaterial coating (e.g., polycatecholamine), or two‐step nanoparticle doping.^[^ ^76^ ^]^ In this work, Graphene@PEDOT:PSS hybrid ink was developed for screen‐printing of three‐electrode designs, providing increased surface area, high conductivity, improved sensitivity, and more importantly enriched functional moieties for immobilization of capture antibodies without any intermediate preparation steps. The screen‐printing process allows for reproducible production of the bare electrode, characterized as a quality control step using the EIS technique to ensure a similar electrical response (RSD <3%) in different batches stored in the laboratory environment over a 6 month period. Also, the antibody immobilization step was performed in a reproducible manner, with a repeatable coating process for each set of experiments, resulting in an RSD <9%, for three replications. Also, the immunosensor response in detecting spiked N‐protein samples was also reproducible with RSD <10% for both WEs.

The application of the immunosensor for the detection of COVID‐19 patients was assessed utilizing 105 clinical samples. This ultrasensitive and rapid antigen immunoassay accurately detected clinical SARS‐CoV‐2 positive NP or throat swab samples with a sensitivity of 91% compared to RT‐PCR, in patients with and without symptoms both in high‐ and low‐prevalence scenarios. Although the results of this work should not be considered as clinical validation of CoVSense, larger prospective clinical validation is warranted on nonarchived samples. CoVSense proved to have a dynamic detection range of 1–1000 pg mL^−1^, the sensitivity of 5037 and 4883 Ω mL pg mm^−2^ and LoD of 421 fg mL^−1^ for WE1, and 410 fg mL^−1^ for WE2, for diagnosis of N‐proteins in spiked samples. Several other immunosensors have been introduced for the detection of N‐proteins in swab samples, including works done by Eissa et al.^[^ ^51^ ^]^ on the development of electrochemical immunosensors, while other studies incorporating techniques, such as chemiluminescence^[^ ^77^ ^]^ or optoelectrical sensing.^[^ ^51^, ^78^ ^]^ Among the electrochemical immunosensors used for N‐protein detection, the sensor prepared by Eissa et al., in which the carbon electrodes were modified with gold‐nanoparticle functionalized with 11‐mercaptoundecanoic acid (MUA), showed the lowest LoD of 400 fg mL^−1^. Apart from the complexity of the fabrication of such sensors, the clinical performance of these sensors in a large clinical cohort, in comparison with RT‐PCR and in detecting samples with low viral load has not been examined. On the other hand, the Simoa N‐protein assay showed the lowest LoD of 99 fg mL^−1^, where it outperformed all other antigen immunosensors or immunoassays.^[^ ^54^ ^]^ Apart from the complexity of the fabrication of such sensors, the clinical performance of these sensors in a large clinical cohort, in comparison with RT‐PCR and in detecting samples with low viral load has not been examined. On the other hand, Simoa N‐protein assay showed the lowest LoD of 99 fg mL^−1^, where it outperformed all other antigen immunosensors or immunoassays.^[^ ^54^ ^]^ The clinical sensitivity of 96% and specificity of 100% were reported by Simoa N‐protein assay when it was tested on 898 patients, with comparable performance to gold standard qRT‐PCR.^[^ ^79^ ^]^ With the clinical sensitivity of 95% for the samples with Ct value <25 and 91% for all samples, and specificity of 100% presented here by CoVSense for combined symptomatic and asymptomatic patients, it performs as reasonably comparable to Simoa antigen testing assay for detection of SARS‐CoV‐2 but in form of a rapid (<10 min) and PoCT kit.^[^ ^80^ ^]^ Ultrasensitive CoVSense also addresses the false‐negative RT‐PCR results of about 30% for commercial rapid antigen testing kits like Panbio rapid testing kits^[^ ^81^ ^]^ which represented a significant challenge for adapting those POCTs as a diagnostic kit during the COVID‐19 pandemic. Moreover, the reported sensitivity of this study has been achieved through the existence of an additional layer of reliability investigation, considering the two‐working electrode self‐validation mechanism, which might have not been the case for methods considering only one signal for each replicate. It means that we relied on the signals obtained for both working electrodes for our decision making, with positive cases where one WE was positive and one negative, and hence were considered unacceptable instead of positive, while in other techniques it might not be trackable and hence reported as positive, increasing their sensitivity. Notably, in a large study comparing 64 lateral flow assays, only 4 of them (orient Gene, Deepblue, Abbott, and Innova SARS‐CoV‐2 Antigen Rapid Qualitative Test) showed an acceptable sensitivity when tested in a large population with the highest clinical sensitivity of 78%.^[^ ^82^ ^]^ Also, the two rapid antigen testing kits recommended by WHO reported a clinical sensitivity of 68%.^[^ ^82^ ^]^ Moreover, in one of the most comparative studies done in Germany comparing the performance of 122 SARS‐CoV‐2 antigen testing kits tested on a population with 17<Ct<36, the highest clinical sensitivity reported was 86%, while many others showed clinical sensitivity of <80%,^[^ ^83^ ^]^ all demonstrating the reliability of CoVSense, even as a potential diagnostic testing kit, in compared to the present research‐based devices and commercial SARS‐CoV‐2 antigen testing kits.

One standard technique to evaluate the clinical limit of detection of a new assay is to test it with diluted clinical samples while comparing it with the gold standard method. The ideal SARS‐CoV‐2 antigen immunosensors are therefore expected to perform comparably to RT‐PCR in detecting positive samples with the lowest viral load (for RT‐PCR, samples with the highest acceptable Ct value). Based on the CDC guideline,^[^ ^84^ ^]^ the highest acceptable Ct value for detecting positive SARS‐CoV‐2 genes is 37, wherein the ideal antigen immunosensors need to show a positive response for both samples with Ct nearly to 37 and diluted samples reaching a Ct value of nearly to 35. Here, CoVSense demonstrated its capability for detecting positive samples with low Ct values of 36. CoVSense could successfully detect positive SARS‐CoV‐2 samples with a Ct range of 30–35. Also, CoVSense showed its success in detecting positive clinical samples #13 and #15 (with the initial Ct values of 26.49 and 26.33, respectively) after their 1000X dilution (new equivalent Ct of 35 and 35 respectively; *R*_ct_ >120 kΩ). The success of the CoVSense N‐protein immunosensor in detecting intact and diluted clinical samples with low viral load comparable to RT‐PCR is one crucial step forward toward introducing antigen rapid tests as reliable diagnostic kits. More importantly, CoVSense could potentially be employed to diagnose early‐stage COVID‐19 infected patients, with no symptoms and, also with low viral load (Ct value = 33).

Out of 65 SARS‐CoV‐2 clinical samples, CoVSense had negative responses for 6 positive samples (with Ct values of 22.91, 26.64, 34.73, 30.25, 30.61, and 22.56), 4 of which were wildtype SARS‐CoV‐2 samples, and the others were samples with SARS‐CoV‐2 B.1.1.7 variant. The RT‐PCR tests on all clinical samples were performed on fresh samples, while the CoVSense tests were performed on samples stored at −80 °C for at least 10 months. There exist multiple factors affecting the performance of the test, and as a result, false‐negative responses. To evaluate the effect of sample storage (at temperature −80 °C) on the negative response of CoVSense for those 6 positive samples, these samples were retested with qRT‐PCR. The retesting of these samples did not show any change in Ct values, confirming that the negative response of CoVSense on these 6 positive samples is not probably attributed to the sample storage. It is noted that Panbio N‐protein antigen testing kits also showed negative responses for 4 out of 6 positive samples. Given that antigen assays and RT‐PCR detect two different molecules, the negative response of CoVSense on 4 RT‐PCR positive samples does not challenge the sensitivity of the antigen assay unless these samples are proven to be negative for their N‐proteins tested with ultrasensitive and quantitative assays like Simoa. Further testing of these 6 samples with Simoa could unravel whether there have ever existed detectable N‐proteins in these 6 positive samples.

SARS‐CoV‐2 virus has undergone several mutations, which have caused genetic alterations in the population of circulating viral strains. Clinical laboratory personnel and health care providers are hence alerted by FDA on the effect of mutations on test results. These mutations in the specific part of the virus genome may result in false‐negative responses. The drop in the sensitivity of assays, when used for detecting variant samples, especially with lower viral loads, has been reported for both RT‐PCR and antigen testing kits. Noting that most of the positive clinical samples with negative CoVSense responses (4 out of 6) in this work are clinical samples with the SARS‐CoV‐2 B.1.1.7 variant, one may argue that employing more selective antibodies to the B.1.1.7 variant can improve the sensitivity of antigen testing kits. This strategy is though impractical to implement given the unknown type of mutation for each clinical sample. Based on the FDA guideline, molecular tests developed to detect multiple genetic targets of SARS‐CoV‐2 are less susceptible to genetic variations than those used to detect a single genetic target.^[^ ^85^ ^]^ A similar strategy can be further implemented for the detection of multiple antigen targets of SARS‐CoV‐2 in one single kit, making antigen testing kits less likely to be impacted by the increased prevalence of genetic variants. Electrochemical immunosensors are well‐positioned to perform this role using their low‐cost multiplex POCT. The dual N‐protein immunosensor used for self‐verification in this work can be further adapted to simultaneously detect nucleocapsid and spiked proteins on the same sensor unit. Multiplex electrochemical immunosensors can also be developed to detect viral antigens of different target respiratory viruses and therefore provide the ability to differentiate SARS‐CoV‐2 from other respiratory viruses. This is imperative particularly in influenza seasons due to the similarity of COVID‐19 and influenza clinical symptoms, which later on demand different disease management.

There are, however, several restrictions subjected to this study: limited accessibility to clinical samples particularly control samples, inaccurate data of clinical symptoms of some patients, and lack of access to a reference quantitative antigen testing assay like Simoa. The cohorts tested were predominantly selected from patients admitted to the hospitals, which can illustrate N‐protein concentrations in severely infected individuals. Most samples were collected from residents mainly of older ages (mean: 47 yr). We performed clinical sample dilution tests for only two donors. We determined our cut‐off from our initial clinical feasibility test, without incorporating any separate or prospective patient cohort, although the cut‐off value was further verified by the larger clinical validation. The cut‐off presented here is preliminary and may change upon further testing the CoVSense in larger cohorts. A prospective sample collection procedure is now in progress, which will help to characterize CoVSense in a more solid cohort of positive and control samples. Further clinical validation studies and arrangement with Simoa is also ongoing to perform correlation analysis between CoVSense and Simoa N‐protein assays in a prospective clinical cohort.

One critical advantage of our immunosensor assay is the rapid response of the testing kit. The results of optimizing the incubation time show that CoVSense is expected to detect positive samples as quickly as even 2 min testing and less than 10 min sample‐to‐result turnaround time. Similar rapid antigen testing kits have already been demonstrated for the detection of SARS‐CoV‐2. However, almost all RT‐PCR and isothermal assays still require at least 30 min to reliably detect clinical samples.^[^ ^86^ ^]^ The rapid response of antigen testing kits, once proven to be clinically applicable for diagnostic purposes, can be a game‐changer for settings like airports, travels, and schools, where immediate COVID response is well, merited. While the assay incubation period is 10 min, the reader only needs up to 2 min to detect and analyze the signals for each immunosensor, presenting this detection method as relatively high‐throughput. Further integration of the CoVSense sensor into autonomous microfluidic assays, automating the entire process of electrochemical biosensors, can create true sample‐to‐result antigen testing kits with comparable clinical performance to RT‐PCR for rapid diagnosis of SARS‐CoV‐2. The detection of antigen using our low‐cost, battery‐powered, hand‐held, and CoVSense‐customized potentiostat makes this testing platform an easy‐to‐conduct rapid antigen test near patients, in a community setting, emergency rooms, outpatient facilities, in places with limited resources, and with limited access to molecular diagnostics such as in developing countries where an outbreak has occurred, and for population‐wide screening for SARS‐CoV‐2 infection. CoVSense platform has all the capabilities needed for advancement toward quantitative N‐protein detection.

There are several benefits for quantitative measurement of antigen concentrations with respect to qualitative assessments. Quantification of viral loads not only helps to have a better objective decision for diagnosis purposes but also assists to hint the stage and kinetic of infection and the risk of transmission, notify seroconversion, and is an indication of the risk of progression and increased severity.^[^ ^87^ ^]^ A positive result with an indication of the high viral load makes the tested person a highly contagious individual for spreading the disease or used as a management tool for dismissing the patients from the hospital's infectious disease wards.^[^ ^88^ ^]^ A patient with a lower viral load, on the other hand, is possibly less prone to cause immediate disease spread. Also, the viral load can be attributed to either the progression of the disease, which enables the illness's diagnosis during the next rounds of physician's examinations, or subsequently decreases as part of the natural recovery from infection. The results on diluted clinical samples indicated that CoVSense could detect clinical swab samples even after their dilution for 1000 times, therefore it is expected to accurately measure variation in concentration of SARS‐CoV‐2 N‐proteins in clinical samples with high or moderate viral loads during the first weeks after infection. However, longitudinal quantification of viral load requires low‐cost and easy‐to‐use antigen testing kits while employing noninvasive sampling. Further testing of CoVSense with saliva samples of infected patients for quantification and monitoring of their viral loads may present a low‐cost solution for monitoring the stage of infection.

The stability of CoVSense for detecting SARS‐CoV‐2 N‐protein clinical samples was examined in this study within 5 h of removing the kits from 4 °C storage. Although this window fulfills the requirements of most test settings, further investigation is needed to examine the stability and shelf‐life of CoVSense for longer periods. It is noted that the room temperature storage of these kits may need replacing the capture antibodies with selective thermostable aptamers specifically developed for the detection of N‐proteins.

In the future, we will integrate on‐chip lysis into the microfluidic unit to provide access to the antigens within the nonlysed cells, with a promise to improve the sensitivity of sensors and eliminate the risk of false‐negative responses. We will also implement the wireless data transfer unit of the bi‐potentiostat to communicate and have access to the Cloud so that the testing result can be verified on the Cloud, while the cut‐off values are updated on a real‐time basis and under future machine learning models implemented.

## 4 Experimental Section

### Electrochemical Dual‐Sensing Electrode Fabrication

The two‐working electrode design of the immunosensor was sketched utilizing AutoCAD 2021 Version R.47.0.0, based on dimensional constraints imposed by the commercial connectors (Molex, 047 286 1001) and conventionally used immunosensing strips. The electrode design was fabricated using a semiautomated polyester‐meshed screen‐printer, Micro Flatbed Printer (A.W.T. World Trade, Inc., USA), upon the development of the hybrid ink, which contains graphene nanosheets and poly(3,4‐ethylenedioxythiophene) polystyrene sulfonate (PEDOT:PSS) conductive polymers dispersed in DMF (Sigma, USA) and is mechanically intermixed with a base carbon ink (7102, Dupont, USA), upon addition of DAAM (Fisher Scientific, Canada) as a binder agent to achieve high uniformity and enhance the miscibility. In brief, the primary Graphene@PEDOT:PSS ink was exposed to ultrasonic mixing in tandem with heating at 100 °C to induce total exfoliation or delamination of all graphene sheets and enhance access to all their contained functional groups. This heating step continued for 30 min, while DAAM binders were gradually added to the mixture starting from minute 15th. Following the printing of the electrodes with the intended design, the curing process was performed via an InfraRed curing machine (Customized Natgraph Air Force Dryer, Natgraph Ltd., UK). For directing the carboxyl‐rich graphene sheets to the surface, the sheets were heated at 150 °C for 5 min followed by cooling down to the fan temperature (≈ 15 °C). The strips contain carboxyl functional groups needed for antibody immobilization, hence requiring no additional cross‐linking or functionalization steps. As for the insulating mask needed to cover conductive areas except for the four electrodes of the electrochemical cell, a medical‐grade double‐sided adhesive sheet (ARcare 90106NB, Adhesive Research Inc., USA) was laser cut (Speedy 360 flex CO_2_ laser engraver machine, Trotec, USA) and placed on the strips before proceeding with antibody immobilization.

### CoVSense Reader Fabrication

The portable custom‐made readout system utilized a microcontroller (MC) as its main component, which conducts Analog to Digital (ADC) and digital‐to‐analog (DAC) signal processing with high resolution. The microcontroller‐based impedance measurement system relied on the time‐based analysis of amplitude and phase shift of sinusoidal excitation and response signals. The resolution of the measurement is 1% of the nominal amplitude. The measurement of the phase is possible from 0° to 180° with a fixed resolution of 6.5°. The proposed low‐complexity system is composed of an ARM Cortex M3 microcontroller for impedance analysis and an ARM Cortex‐A microprocessor for user configuration and data visualization. For rapidly and accurately performing the EIS measurements, seven different frequencies of 1, 3, 11, 37, 125, 412, and 1390 Hz was selected for data sampling points, and the least‐square curve fitting method was employed for obtaining the Nyquist diagrams, and consequently the charge transfer resistance (*R*_ct_) values. The reader's electronic board (EIS module) is connected to a low‐powered single‐board computer using a standard serial port to provide higher computational power for model analysis, advanced graphical interface, and wireless access to the Cloud through the internet or local network. The boards integrated with a 3.5 in. liquid crystal display (LCD), ICR18500 rechargeable battery, and charging module are accommodated into a custom design box using a 3D printer to avoid any leakage between the device interior and environment. The entire CoVSense readout system is hence handheld and user‐friendly, and thus, could be used for quantitative and digital POCT (Supporting Information S6, Figures S8–S15, and Tables S5 and S6, Supporting Information).

### N‐Protein Antigen Immunosensor Preparation

Immunosensors were independently prepared in a separate laboratory, by placing 4 µL of 50 µg mL^−1^ N‐protein antibody (HC2003, GenScript Inc., USA), and for control assessments, anti‐SARS‐CoV‐2 S‐protein antibody (#HC2001, Genscript Inc., USA) prepared in PBS, on each working electrode, and incubated at 4 °C overnight. Upon rinsing the electrode, the antibody‐immobilized electrodes were incubated with 0.005% BSA (#A2153, Sigma, USA), for 20 min, prior to proceeding to clinical sample testing.

### Clinical Sample Testing

All clinical samples analyzed in this study were provided by Alberta Public Health Laboratory (Alberta Precision Laboratories, Calgary, AB) which were at first stored in UTM for clinical diagnosis of SARS‐CoV‐2 and maintained at −80 °C after testing. They were tested for validating the existence of the SARS‐CoV‐2 virus or other viral types using RT‐PCR with the established cut‐off of Ct value less than 35 for a positive test at the university hospital's diagnostic lab. Samples were stored and tested in a specialized laboratory for infectious pathogens. Nasopharyngeal or throat clinical samples acquired from patients, stored in cryo‐vials, were thawed at room temperature. Samples were then drop‐casted on the working electrodes with no prior lysing, filtering, or heat treatment. Following the optimal 10 min incubation with clinical samples, the electrodes were rinsed, dried under the safety cabinet, and EIS measurements were conducted using 2.5 mm [Fe(CN)_6_]^3−/4−^ redox (a solution of 2.5 mm Potassium ferricyanide (III) (#702 587, Sigma, USA) and 2.5 mm potassium hexacyanoferrate (II) trihydrate (#33 358, Alfa Aesar, Thermo Fisher Scientific., USA)) prepared in 1x PBS. Parallel experimentation was performed using the Panbio Rapid Antigen test kits (Abbott Laboratories, USA), conducted in an off‐label manner, for comparing the functionality of the CoVSense with a commercially available test. Clinical swab samples were assessed in triplicates.

### Characterizations and Instruments

Morphological assessments of the bioready strips and fabricated nano‐immunosensors were performed using FESEM (Sigma‐ ZEISS, Germany) and AFM (Bruker Nanoscope, Germany) in tapping mode. All electrodes were sputtered with gold prior to performing the FESEM imaging for enhanced resolution.

### Statistical Analysis

All data analysis was performed in R v.4.0.3. CoVSense measurements in relation to RT‐PCR results were compared using the mean and the minimum of the measurements per person exhibiting comparable results. Therefore, the downstream analyses were conducted on the mean of the CoVSense measurements per person. Association of CoVSense measurement with different factors was assessed using ANOVA with Tukey post hoc comparison. The relative performance of CoVSense and RT‐PCR were assessed using Spearman rank correlation and Lin's concordance correlation following MinMax normalization of the CoVsense impedance measurements and RT‐PCR Ct values. Lin's concordance correlation was performed using epiR package v. 2.0.26 and used to estimate the concordance correlation coefficient (CCC), precision, and accuracy. Next, to assess the diagnostic performance of CoVSense, the optimum cut‐off threshold using Youden's index using OptimalCutpoints package v.1.1.4 was estimated. It was confirmed that the optimum cut‐off would be the same based on the minimum of the CoVSense measurement. The CoVSense measurements were then categorized into positive or negative according to the identified cut‐off. Subsequently, the diagnostic performance was assessed by sensitivity, specificity, and precision metrics using MLmetrics v.1.1.1, pROC v.1.16.2, and tidymodels v.0.1.3 packages. The post‐test probabilities were estimated using Leaf plot v.0.9. Diagnostic results were categorized as false negative, true negative, and true positive and were compared based on patient characteristics using *χ*2 (post hoc) for categorical factors and ANOVA (Tukey post hoc) for continuous factors. Finally, the diagnostic performance of CoVSense was compared to Panbio using performance metrics including accuracy, AUC, Fr for positive and negative, and recall for positive and negative. Concordance of CoVSense and Panbio results with RT‐PCR results and Ct values were assessed using *χ*2. A *p*‐value of less than 0.05 was considered significant.

### Ethical Statement

The present study has been thoroughly reviewed and approved internally by the University of Calgary's Ethics Board, Alberta, Canada (ethics No.: REB20‐1032). All experiments were performed under the Class II biosafety cabinet according to the bio‐safety protocols set in the ethics. It is noted that no written informed consent was required by the institutional reviewers since the clinical samples were anonymously provided to the research team.

## Conflict of Interest

The authors declare no conflict of interest.

## Author Contributions

Concept and design: R.S.; Electrochemical sensor design and fabrication: R.S.; Electrode physical characterization: R.S., F.H.; Immunosensor electrochemical characterization: R.S., F.H., Immunosensor preparation: R.S, F.H.; Readout/potentiostat hardware development: H.O.T., G. A.C., E.G.Z., R.S., Readout software development: H.O.T., R.S., G.A.C., E.G.Z., A.S.N.; Clinical sample collection, and preparation: R.S., J.E.H.; Clinical sample electrochemical and rapid testing: R.S., J.E.H.; Statistical data analysis: R.S., S.M.; Drafting and revising the manuscript: R.S., J.E.H., F.H., H.O.T., S.K., S.M., B.M.B., Y.D.N., E.G.Z., A.S.N.; Experimentation & data acquisition: R.S., J.E.H., F.H., H.O.T.; Data analysis and/or interpretation: R.S., H.O.T., Y.D.N., E.G.Z., A.S.N.; Clinical sample analysis: R.S., S.K., S.M., B.M.B., Y.D.N., A.S.N.; Drafting and revising the manuscript: R.S., J.E.H., F.H., H.O.T., S.K., S.M., G.A.C., B.M.B., Y.D.N., E.G.Z., A.S.N.

## Supporting information

Supporting Information

Supporting Information

## Data Availability

The data that support the findings of this study are available in the supplementary material of this article.
