# Supplementary material for: CoVSense: Ultrasensitive Nucleocapsid Antigen Immunosensor for Rapid Clinical Detection of Wildtype and Variant SARS‐CoV‐2
Source: Adv Sci (Weinh). 2023 Mar 30;10(15):2206615. doi: 10.1002/advs.202206615 (PMC10214237; doi:10.1002/advs.202206615)
Supplement: Supplementary file 1 — Supporting Information [file ADVS-10-2206615-s002.pdf]

ADVANCED SCIENCE
Open Access

Supporting Information

for *Adv. Sci.*, DOI 10.1002/advs.202206615

CoVSense: Ultrasensitive Nucleocapsid Antigen Immunosensor for Rapid Clinical Detection of Wildtype and Variant SARS-CoV-2

*Razieh Salahandish, Jae Eun Hyun, Fatemeh Haghayegh, Hamed Osouli Tabrizi, Shirin Moossavi, Sultan Khetani, Giancarlo Ayala-Charca, Byron M. Berenger, Yan Dong Niu, Ebrahim Ghafar-Zadeh and Amir Sanati Nezhad**

Supplementary materials

CoVSense: Ultrasensitive nucleocapsid antigen immunosensor for rapid clinical detection of wildtype and variant SARS-CoV-2

Razieh Salahandish[1,2,3], Jae Eun Hyun[4], Fatemeh Haghayegh[1,2], Hamed Osouli Tabrizi[5], Shirin Moossavi[1,6,7], Sultan Khetani[1], GiancarloAyala-Charca[5], Byron M. Berenger[8,9], Yan Dong Niu[4], Ebrahim Ghafar-Zadeh[5], Amir Sanati Nezhad[1,2,10*]

[1]BioMEMS and Bioinspired Microfluidic Laboratory, Department of Biomedical Engineering, University of Calgary; Calgary, Alberta T2N 1N4, Canada

[2]Department of Mechanical and Manufacturing Engineering, University of Calgary; Calgary, Alberta T2N 1N4, Canada

[3]Laboratory of Advanced Biotechnologies for Health Assessments (LAB-HA), Department of Electrical Engineering and Computer Science, Lassonde School of Engineering, York University; Toronto M3J 1P3, Canada

[4]Department of Ecosystem and Public Health, Faculty of Veterinary Medicine, University of Calgary; Calgary, Alberta T2N 1N4, Canada

[5]Biologically Inspired Sensors and Actuators (BioSA), Department of Electrical Engineering and Computer Science, Lassonde School of Engineering, York University; Toronto M3J 1P3, Canada

[6]Department of Physiology and Pharmacology, University of Calgary; Calgary, Alberta T2N 1N4, Canada

[7]International Microbiome Centre, Cumming School of Medicine, Health Sciences Centre, University of Calgary; Calgary, Alberta T2N 1N4, Canada

[8]Alberta Public Health Laboratory, Alberta Precision Laboratories, 3330 Hospital Drive; Calgary, Alberta T2N 4W4, Canada

[9]Department of Pathology and Laboratory Medicine, Faculty of Medicine, University of Calgary; Calgary, Alberta T2N 1N4, Canada

[10]Biomedical Engineering Graduate Program, University of Calgary; Calgary, Alberta T2N 1N4, Canada

S1: Fabrication process of the bio-ready immunosensing strips

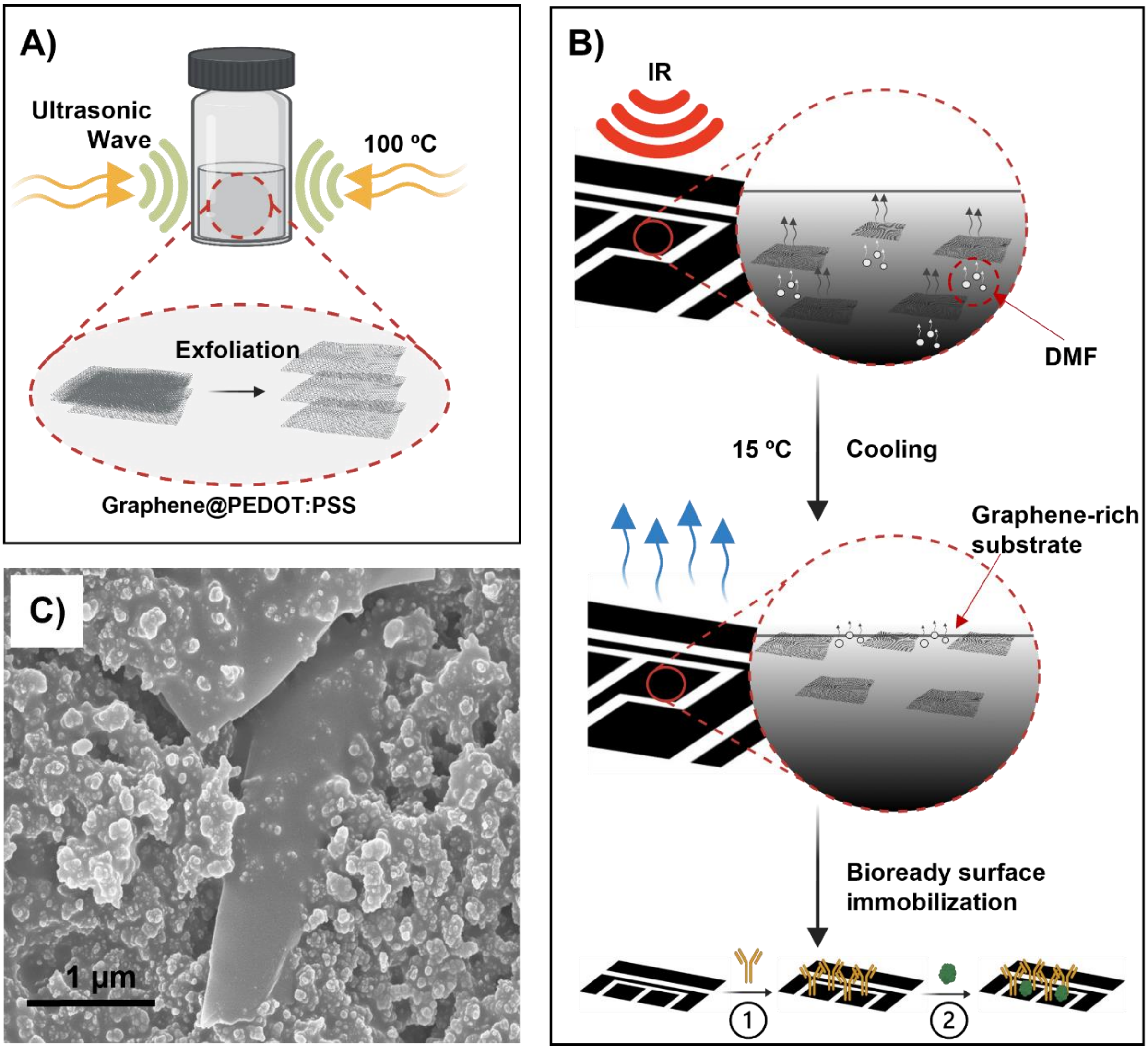

Figure S1. Representation of the second-generation bio-ready strips fabrication process to improve surface concentrations of the graphene nanosheets. A) Fully exfoliating the graphene nanosheets in the primary ink of Graphene@PEDOT:PSS using a coupled ultrasonic and heating process. The ink is heated at 100 °C which is below the evaporation temperature of the dimethylformamide (DMF) solvent. B) Infrared (IR) curing of the ink composition used to evaporate the DMF solvent, resulted in directing the graphene nanosheets to the surface upon cooling, and creating a graphene-rich bioready surface potent for direct immobilization of (1) antibodies, and (2) surface passivation using bovine serum albumin (BSA) towards the immunosensor fabrication. C) Field emission scanning electron microscopy (FESEM) image of the adjacent fully exfoliated transparent graphene nanosheets on the graphene-rich surface.

S2: Electrode morphological characterization using Atomic Force Microscopy (AFM)

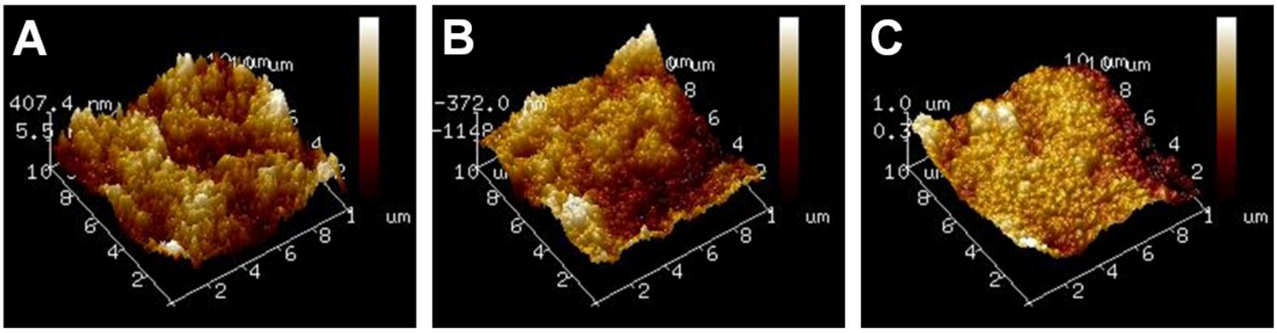

Figure S2. Physical characterization of the sensing strip using Atomic Force Microscopy (AFM) images obtained from **(A)** pure carbon bare electrode, **(B)** Graphene@PEDOT:PSS-Carbon (GPePC) screen-printed electrode, and **(C)** antibody immobilized nano-immunosensor.

S3: CoVSense functionality

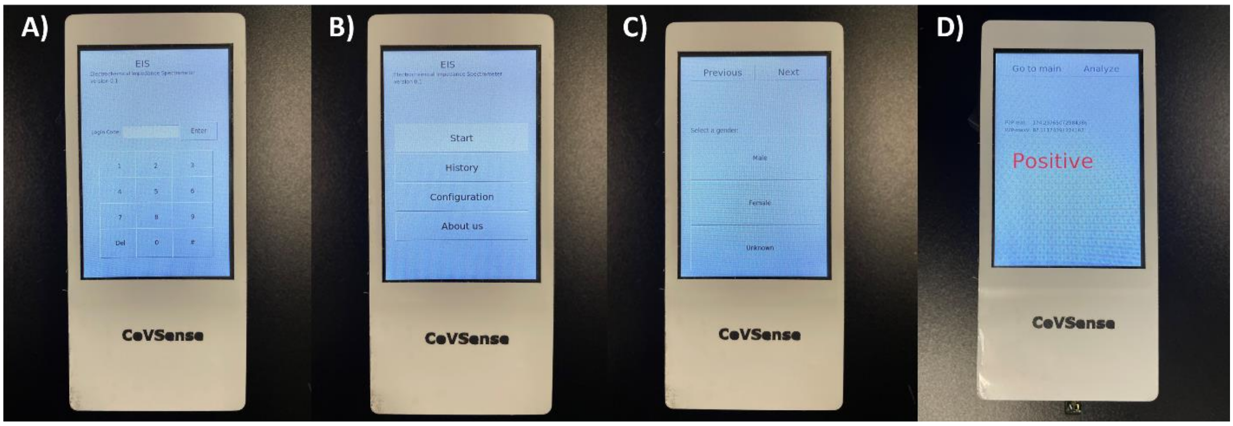

Figure S3. Portable and easy-to-use CoVSense potentiostat device and its step-by-step functionality. (A) The user logs in utilizing a specified identifier number. **(B)** The Electrochemical Impedance Spectroscopy (EIS) test is initiated. **(C)** The patient-specific data are entered in an embedded questionnaire. **(D)** The result of the test is represented upon completion of the measurement.

S4: Additional control assessments for the electrochemical sensor's reliability assurance

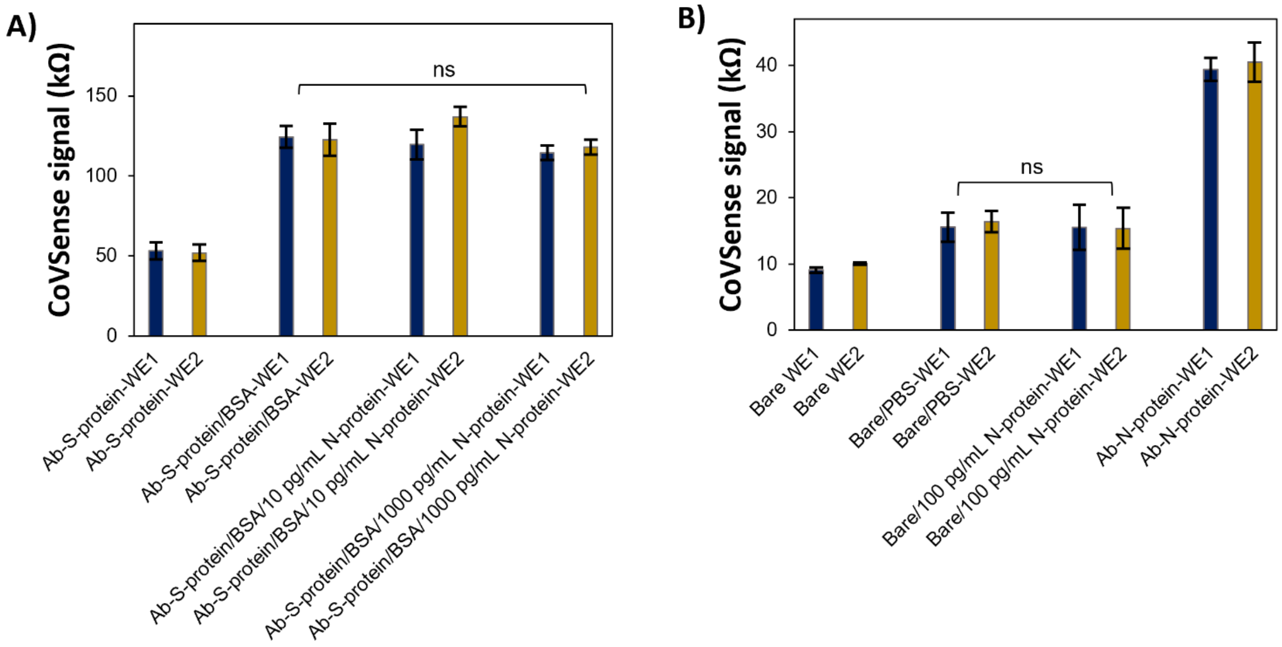

Figure S4. Electrochemical impedance spectroscopy (EIS) signals obtained from (A) the strips coated with the spike (S)-protein antibodies and subject to the nucleocapsid (N)-protein antigens, and **(B)** the bare electrodes incubated with phosphate-buffered saline (PBS) and N-protein antigens with no prior antibody immobilization.

S5: Clinical samples information

Table S1. Stability of CoVSense kit in ambient condition for up to 5 hours of opening the testing kit.

Patient ID	Impedance (kΩ)	Std. Err	Patient ID	Impedance (kΩ)	Std. Err
**3:00 hr - 3:30 hr**			**3:30 hr - 4:00 hr**		
**Naso 1**	82.49667	1.39021	**Naso 2**	64.84667	5.62379
**Naso 5**	83.55	6.85347	**Naso 6**	97.84	5.69213
**Naso 9**	73.98333	5.89121	**Naso 10**	84.61	7.07785
**Naso 13**	99.01667	3.55762	**Naso 14**	93.55	6.75569
**Naso 17**	90.30333	10.0086	**Naso 18**	59.27667	5.98079
**Naso 21**	84.48333	2.26546	**Naso 22**	91.14667	3.7642
**Naso 25**	75.30667	2.63521	**Naso 26**	68.02333	8.55948
**Naso 29**	94.03	3.74882	**Naso 30**	106.15667	4.33257
**4:00 hr - 4:30 hr**			**4:30 hr - 5:00 hr**		
**Naso 3**	91.13333	4.40607	**Naso 4**	89.12333	3.63557
**Naso 7**	95.94667	10.7431	**Naso 8**	100.03333	8.68048
**Naso 11**	99.91	2.36229	**Naso 12**	96.11333	8.89841
**Naso 15**	100.99667	7.05673	**Naso 16**	95.37667	7.99128
**Naso 19**	106.83	5.00317	**Naso 20**	80.38	8.2818
**Naso 23**	106.73333	1.17545	**Naso 24**	95.46	7.11787
**Naso 27**	102.68	1.86605	**Naso 28**	79.74	4.17845

Table S2. The characteristics information of the patients.

Sample ID	Patient ID	Patient gender	Specimen source	Target	Specimen type	Specimen age	Symptomatic/asymptomatic based on lab database	Symptom Onset-Swab Date (d)
**S1**	Naso 1	Female	Nasopharyngeal Swab	None	Unknown	10	Yes Symptomatic	Missing
**S2**	Naso 2	Male	Nasopharyngeal Swab	None	Community Patient	86	Yes Symptomatic	Missing
**S3**	Naso 3	Female	Nasopharyngeal Swab	None	Emergency	42	Missing	Missing
**S4**	Naso 4	Female	Nasopharyngeal Swab	None	Community Patient	65	Yes Symptomatic	Missing
**S5**	Naso 5	Male	Nasopharyngeal Swab	None	Emergency	46	Missing	Missing
**S6**	Naso 6	Male	Nasopharyngeal Swab	None	Inpatient	67	No Asymptomatic	Missing
**S7**	Naso 7	Female	Nasopharyngeal Swab	None	Unknown	26	Yes Symptomatic	Missing
**S8**	Naso 8	Female	Nasopharyngeal Swab	None	Emergency	31	Yes Symptomatic	Missing
**S9**	Naso 9	Male	Nasopharyngeal Swab	None	Inpatient	74	Missing	Missing

Table S2 continued.

Sample ID	Patient ID	Patient gender	Specimen source	Target	Specimen type	Specimen age	Symptomatic/asymptomatic based on lab database	Symptom Onset-Swab Date (d)
**S10**	Naso 10	Female	Nasopharyngeal Swab	None	Inpatient	70	Missing	Missing
**S11**	Naso 11	Male	Nasopharyngeal Swab	None	Community Patient	84	Yes Symptomatic	Missing
**S12**	Naso 12	Male	Nasopharyngeal Swab	None	Community Patient	33	Yes Symptomatic	Missing
**S13**	Naso 13	Female	Nasopharyngeal Swab	None	Community Patient	94	Yes Symptomatic	Missing
**S14**	Naso 14	Female	Nasopharyngeal Swab	None	Inpatient	81	No Asymptomatic	Missing
**S15**	Naso 15	Male	Nasopharyngeal Swab	None	Nursing Home	75	No Asymptomatic	Missing
**S16**	Naso 16	Female	Nasopharyngeal Swab	None	Inpatient	78	No Asymptomatic	Missing
**S17**	Naso 17	Female	Nasopharyngeal Swab	None	Emergency	22	No Asymptomatic	Missing
**S18**	Naso 18	Female	Nasopharyngeal Swab	None	Inpatient	30	No Asymptomatic	Missing
**S19**	Naso 19	Male	Nasopharyngeal Swab	None	Community Patient	37	Yes Symptomatic	Missing
**S20**	Naso 20	Female	Nasopharyngeal Swab	None	Inpatient	21	Yes Symptomatic	Missing
**S21**	Naso 21	Male	Nasopharyngeal Swab	None	Inpatient	63	Missing	Missing
**S22**	Naso 22	Male	Nasopharyngeal Swab	None	Inpatient	80	Missing	Missing
**S23**	Naso 23	Female	Nasopharyngeal Swab	None	Inpatient	65	Missing	Missing
**S24**	Naso 24	Male	Nasopharyngeal Swab	None	Inpatient	68	Missing	Missing
**S25**	Naso 25	Male	Nasopharyngeal Swab	None	Inpatient	55	Missing	Missing
**S26**	Naso 26	Female	Nasopharyngeal Swab	None	Inpatient	35	Missing	Missing
**S27**	Naso 27	Female	Nasopharyngeal Swab	None	Inpatient	51	Missing	Missing
**S28**	Naso 28	Male	Nasopharyngeal Swab	None	Inpatient	69	Missing	Missing
**S29**	Naso 29	Female	Nasopharyngeal Swab	None	Inpatient	87	Missing	Missing
**S30**	Naso 30	Male	Nasopharyngeal Swab	None	Inpatient	38	No Asymptomatic	Missing
**S31**	Naso 31	Female	Nasopharyngeal Swab	None	Emergency	26	Missing	Missing
**S32**	Naso 32	Female	Nasopharyngeal Swab	None	Community Patient	40	No Asymptomatic	Missing
**S33**	Naso 33	Male	Nasopharyngeal Swab	None	Inpatient	67	Missing	Missing
**S34**	Naso 34	Male	Nasopharyngeal Swab	None	Unknown	22	No Asymptomatic	Missing
**S35**	Naso 35	Male	Nasopharyngeal Swab	None	Inpatient	35	Missing	Missing

S36	Naso 36	Missing	Nasopharyngeal Swab	FLUA	Hospital	57	Missing	Missing

Table S2 continued.

Sample ID	Patient ID	Patient gender	Specimen source	Target	Specimen type	Specimen age	Symptomatic/asymptomatic based on lab database	Symptom Onset-Swab Date (d)
S37	Naso 37	Male	Nasopharyngeal Swab	FLUA	Community	44	Missing	Missing
S38	Naso 38	Male	Nasopharyngeal Swab	FLUB	Community	6	Missing	Missing
S39	Naso 39	Female	Auger Suction	RSV	ER	1y8m	Missing	Missing
S40	Naso 40	Female	Nasopharyngeal Swab	RSV	Hospital	53	Missing	Missing
S41	1	Female	Nasopharyngeal Swab	COVID	Unknown	34	Yes Symptomatic	2
S42	2	Female	Nasopharyngeal Swab	COVID	Unknown	54	No Asymptomatic	0
S43	3	Male	Nasopharyngeal Swab	COVID	Unknown	22	Yes Symptomatic	3
S44	4	Male	Nasopharyngeal Swab	COVID	Unknown	43	Yes Symptomatic	3
S45	5	Male	Nasopharyngeal Swab	COVID	Inpatient	39	Missing	2
S46	6	Female	Nasopharyngeal Swab	COVID	Inpatient	74	Missing	1
S47	7	Female	Nasopharyngeal Swab	COVID	Community Patient	52	Yes Symptomatic	1
S48	8	Male	Nasopharyngeal Swab	COVID	Community Patient	33	Yes Symptomatic	0
S49	9	Female	Nasopharyngeal Swab	COVID	Community Patient	30	Yes Symptomatic	0
S50	10	Female	Nasopharyngeal Swab	COVID	Emergency	97	Yes Symptomatic	0
S51	11	Male	Nasopharyngeal Swab	COVID	Inpatient	80	Yes Symptomatic	9
S52	12	Female	Nasopharyngeal Swab	COVID	Community Patient	18	No Asymptomatic	0
S53	13	Male	Nasopharyngeal Swab	COVID	Inpatient	73	Yes Symptomatic	7
S54	14	Male	Nasopharyngeal Swab	COVID	Community Patient	19	No Asymptomatic	0
S55	15	Male	Nasopharyngeal Swab	COVID	Community Patient	56	Missing	0
S56	16	Male	Nasopharyngeal Swab	COVID	Emergency	41	Missing	0
S57	17	Male	Nasopharyngeal Swab	COVID	Community Patient	30	Yes Symptomatic	0
S58	18	Female	Nasopharyngeal Swab	COVID	Unknown	38	Yes Symptomatic	Missing
S59	19	Male	Nasopharyngeal Swab	COVID	Community Patient	35	Missing	0
S60	20	Male	Nasopharyngeal Swab	COVID	Unknown	35	Missing	0

**S61**	21	Male	Nasopharyngeal Swab	COVID	Unknown	45	Yes Symptomatic	1
**S62**	22	Male	Nasopharyngeal Swab	COVID	Unknown	22	Yes Symptomatic	0

Table S2 continued.

Sample ID	Patient ID	Patient gender	Specimen source	Target	Specimen type	Specimen age	Symptomatic/asymptomatic based on lab database	Symptom Onset-Swab Date (d)
**S63**	23	Female	Nasopharyngeal Swab	COVID	Community Patient	49	Yes Symptomatic	5
**S64**	24	Female	Nasopharyngeal Swab	COVID	Community Patient	93	No Asymptomatic	0
**S65**	25	Male	Nasopharyngeal Swab	COVID	Emergency	49	Yes Symptomatic	1
**S66**	26	Female	Nasopharyngeal Swab	COVID	Community Patient	32	Yes Symptomatic	0
**S67**	27	Male	Nasopharyngeal Swab	COVID	Unknown	24	No Asymptomatic	Missing
**S68**	28	Male	Nasopharyngeal Swab	COVID	Community Patient	77	No Asymptomatic	0
**S69**	29	Female	Nasopharyngeal Swab	COVID	Community Patient	48	No Asymptomatic	0
**S70**	30	Male	Nasopharyngeal Swab	COVID	Unknown	29	No Asymptomatic	2
**S71**	UK1	Female	Throat	COVID	Community Patient	38	Yes Symptomatic	2
**S72**	UK2	Male	Nasopharyngeal Swab	COVID	Unknown	80	Missing	14
**S73**	UK3	Male	Nasopharyngeal Swab	COVID	Community Patient	13	Missing	4
**S74**	UK4	Female	Throat	COVID	Community Patient	72	Yes Symptomatic	6
**S75**	UK5	Female	Throat	COVID	Unknown	21	Yes Symptomatic	3
**S76**	UK6	Female	Nasopharyngeal Swab	COVID	Community Patient	41	Missing	3
**S77**	UK7	Male	Throat	COVID	Community Patient	23	Yes Symptomatic	4
**S78**	UK8	Female	Throat	COVID	Community Patient	23	Yes Symptomatic	3
**S79**	UK9	Female	Throat	COVID	Community Patient	37	Yes Symptomatic	2
**S80**	UK10	Female	Throat	COVID	Unknown	13	Yes Symptomatic	0
**S81**	UK11	Female	Nasopharyngeal Swab	COVID	Community Patient	54	Missing	6
**S82**	UK12	Male	Throat	COVID	Unknown	45	Yes Symptomatic	1

**S83**	UK13	Female	Throat	COVID	Unknown	23	No Asymptomatic	1
**S84**	UK14	Female	Throat	COVID	Community Patient	37	Yes Symptomatic	5
**S85**	UK15	Female	Throat	COVID	Unknown	24	Yes Symptomatic	7

Table S2 continued.

Sample ID	Patient ID	Patient gender	Specimen source	Target	Specimen type	Specimen age	Symptomatic/asymptomatic based on lab database	Symptom Onset-Swab Date (d)
**S86**	UK16	Male	Throat	COVID	Unknown	47	Missing	1
**S87**	UK17	Male	Throat	COVID	Unknown	34	Yes Symptomatic	0
**S88**	UK18	Female	Nasopharyngeal Swab	COVID	Unknown	41	Yes Symptomatic	2
**S89**	UK19	Female	Throat	COVID	Unknown	18	Missing	6
**S90**	UK20	Female	Throat	COVID	Community Patient	20	Yes Symptomatic	5
**S91**	UK21	Missing	Swab, No Source Indicated	COVID	Unknown	14	Missing	Missing
**S92**	UK22	Female	Nasopharyngeal Swab	COVID	Community Patient	34	Missing	0
**S93**	UK23	Male	Throat	COVID	Community Patient	43	Yes Symptomatic	0
**S94**	UK24	Male	Throat	COVID	Community Patient	27	Yes Symptomatic	4
**S95**	UK25	Male	Nasopharyngeal Swab	COVID	Community Patient	42	Missing	8
**S96**	UK26	Male	Throat	COVID	Community Patient	10	Yes Symptomatic	3
**S97**	UK27	Male	Throat	COVID	Community Patient	11	Yes Symptomatic	2
**S98**	UK28	Female	Nasopharyngeal Swab	COVID	Community Patient	56	Missing	3
**S99**	UK29	Female	Nasopharyngeal Swab	COVID	Community Patient	54	Missing	4
**S100**	UK30	Female	Throat	COVID	Community Patient	17	Yes Symptomatic	5
**S101**	UK31	Male	Throat	COVID	Community Patient	47	Yes Symptomatic	4
**S102**	UK32	Female	Throat	COVID	Community Patient	29	Yes Symptomatic	4
**S103**	UK33	Male	Throat	COVID	Community Patient	56	Yes Symptomatic	7

**S104**	UK34	Male	Throat	COVID	Community Patient	24	Yes Symptomatic	7
**S105**	UK35	Female	Throat	COVID	Community Patient	34	Yes Symptomatic	7

Table S3a. The attributes of clinical nasopharyngeal and throat swab control samples.

Patient ID	Mean Impedance (kΩ)	Specimen type	Specimen source	Target	Ct value	Pureness
**Naso 1**	82.50	Swab	Nasopharyngeal Swab	None	Collected before COVID-19	No tissue
**Naso 2**	64.85	Swab	Nasopharyngeal Swab	None	Collected before COVID-19	No tissue
**Naso 3**	91.13	Swab	Nasopharyngeal Swab	None	Collected before COVID-19	No tissue
**Naso 4**	89.12	Swab	Nasopharyngeal Swab	None	Collected before COVID-19	No tissue
**Naso 5**	83.55	Swab	Nasopharyngeal Swab	None	Collected before COVID-19	No tissue
**Naso 6**	97.84	Swab	Nasopharyngeal Swab	None	Collected before COVID-19	No tissue
**Naso 7**	95.95	Swab	Nasopharyngeal Swab	None	Collected before COVID-19	No tissue
**Naso 8**	100.03	Swab	Nasopharyngeal Swab	None	Collected before COVID-19	No tissue
**Naso 9**	73.98	Swab	Nasopharyngeal Swab	None	Collected before COVID-19	No tissue
**Naso 10**	84.61	Swab	Nasopharyngeal Swab	None	Collected before COVID-19	No tissue
**Naso 11**	99.91	Swab	Nasopharyngeal Swab	None	Collected before COVID-19	No tissue
**Naso 12**	96.11	Swab	Nasopharyngeal Swab	None	Collected before COVID-19	Big tissues
**Naso 13**	99.02	Swab	Nasopharyngeal Swab	None	Collected before COVID-19	No tissue
**Naso 14**	93.55	Swab	Nasopharyngeal Swab	None	Collected before COVID-19	No tissue
**Naso 15**	101.00	Swab	Nasopharyngeal Swab	None	Collected before COVID-19	No tissue
**Naso 16**	95.38	Swab	Nasopharyngeal Swab	None	Collected before COVID-19	No tissue
**Naso 17**	90.30	Swab	Nasopharyngeal Swab	None	Collected before COVID-19	No tissue
**Naso 18**	59.28	Swab	Nasopharyngeal Swab	None	Collected before COVID-19	No tissue
**Naso 19**	106.83	Swab	Nasopharyngeal Swab	None	Collected before COVID-19	No tissue
**Naso 20**	80.38	Swab	Nasopharyngeal Swab	None	Collected before COVID-19	No tissue
**Naso 21**	84.48	Swab	Nasopharyngeal Swab	None	Collected before COVID-19	No tissue
**Naso 22**	91.15	Swab	Nasopharyngeal Swab	None	Collected before COVID-19	No tissue
**Naso 23**	106.73	Swab	Nasopharyngeal Swab	None	Collected before COVID-19	No tissue
**Naso 24**	95.46	Swab	Nasopharyngeal Swab	None	Collected before COVID-19	No tissue
**Naso 25**	75.31	Swab	Nasopharyngeal Swab	None	Collected before COVID-19	No tissue

**Naso 26**	68.02	Swab	Nasopharyngeal Swab	None	Collected before COVID-19	No tissue
**Naso 27**	102.68	Swab	Nasopharyngeal Swab	None	Collected before COVID-19	No tissue
**Naso 28**	79.74	Swab	Nasopharyngeal Swab	None	Collected before COVID-19	No tissue
**Naso 29**	94.03	Swab	Nasopharyngeal Swab	None	Collected before COVID-19	No tissue
**Naso 30**	106.16	Swab	Nasopharyngeal Swab	None	Collected before COVID-19	No tissue

Table S3a continued.

**Patient ID**	**Mean Impedance (kΩ)**	**Specimen type**	**Specimen source**	**Target**	**Ct value**	**Pureness**
**Naso 31**	91.35	Swab	Nasopharyngeal Swab	None	>37	No tissue
**Naso 32**	92.78	Swab	Nasopharyngeal Swab	None	>37	No tissue
**Naso 33**	105.98	Swab	Nasopharyngeal Swab	None	>37	No tissue
**Naso 34**	112.86	Swab	Nasopharyngeal Swab	None	>37	No tissue
**Naso 35**	99.80	Swab	Nasopharyngeal Swab	None	>37	No tissue
**Naso 36**	96.69	Swab	Nasopharyngeal Swab	FLUA	>37	No tissue
**Naso 37**	92.40	Swab	Nasopharyngeal Swab	FLUA	>37	No tissue
**Naso 38**	105.88	Swab	Nasopharyngeal Swab	FLUB	>37	Big tissues
**Naso 39**	106.52	Fluid	Auger Suction	RSV	>37	No tissue
**Naso 40**	82.78	Swab	Nasopharyngeal Swab	RSV	>37	No tissue

Table S3b. The attributes of clinical SARS-CoV-2 positive nasopharyngeal and throat swab samples.

**Patient ID**	**Mean Impedance (kΩ)**	**Specimen type**	**Specimen source**	**Target**	**Ct value**	**Pureness**
**1**	218.41	Swab	Nasopharyngeal Swab	COVID	19.16	With tissues
**2**	192.40	Swab	Nasopharyngeal Swab	COVID	20.7	With tissues
**3**	171.37	Swab	Nasopharyngeal Swab	COVID	21.14	With tissues
**4**	88.30	Swab	Nasopharyngeal Swab	COVID	22.91	With tissues
**5**	195.04	Swab	Nasopharyngeal Swab	COVID	21.24	With tissues
**6**	166.54	Swab	Nasopharyngeal Swab	COVID	23.17	With tissues
**7**	173.55	Swab	Nasopharyngeal Swab	COVID	22.52	With tissues
**8**	138.98	Swab	Nasopharyngeal Swab	COVID	22.84	With tissues
**9**	127.17	Swab	Nasopharyngeal Swab	COVID	22.67	Big tissues
**10**	168.60	Swab	Nasopharyngeal Swab	COVID	21.57	With tissues
**11**	147.75	Swab	Nasopharyngeal Swab	COVID	28.71	With tissues
**12**	174.52	Swab	Nasopharyngeal Swab	COVID	26.4	With tissues

**13**	190.93	Swab	Nasopharyngeal Swab	COVID	26.49	With tissues
**14**	156.73	Swab	Nasopharyngeal Swab	COVID	26.38	No tissue
**15**	189.29	Swab	Nasopharyngeal Swab	COVID	26.33	With tissues
**16**	166.86	Swab	Nasopharyngeal Swab	COVID	26.44	Big tissues
**17**	190.27	Swab	Nasopharyngeal Swab	COVID	26.43	Beads of tissues

Table S3b continued.

Patient ID	Mean Impedance (kΩ)	Specimen type	Specimen source	Target	Ct value	Pureness
**18**	108.60	Swab	Nasopharyngeal Swab	COVID	26.49	Beads of tissues
**19**	166.42	Swab	Nasopharyngeal Swab	COVID	26.42	Beads of tissues
**20**	151.65	Swab	Nasopharyngeal Swab	COVID	25.62	Beads of tissues
**21**	152.59	Swab	Nasopharyngeal Swab	COVID	33.06	No tissue
**22**	122.37	Swab	Nasopharyngeal Swab	COVID	35.66	No tissue
**23**	156.35	Swab	Nasopharyngeal Swab	COVID	32.67	Tiny tissues
**24**	169.28	Swab	Nasopharyngeal Swab	COVID	31.14	Tiny tissues
**25**	186.58	Swab	Nasopharyngeal Swab	COVID	31.21	Tiny tissues
**26**	165.57	Swab	Nasopharyngeal Swab	COVID	32.77	Tiny tissues
**27**	169.36	Swab	Nasopharyngeal Swab	COVID	31.17	No tissue-Thick like saliva
**28**	146.83	Swab	Nasopharyngeal Swab	COVID	32.74	Big tissues
**29**	154.41	Swab	Nasopharyngeal Swab	COVID	32.75	No tissue
**30**	171.78	Swab	Nasopharyngeal Swab	COVID	31.24	No tissue
**UK1**	252.05	Swab	Throat	COVID	19. 0	No tissue-Very thick like Saliva
**UK2**	246.35	Swab	Nasopharyngeal Swab	COVID	20.57	No tissue-Very thick like Saliva
**UK3**	220.44	Swab	Nasopharyngeal Swab	COVID	19.23	No tissue-Very thick like Saliva
**UK4**	244.17	Swab	Throat	COVID	19.11	No tissue-Very thick like Saliva
**UK5**	186.50	Swab	Throat	COVID	24.91	Little bit Jelly
**UK6**	253.88	Swab	Nasopharyngeal Swab	COVID	17.41	No tissue
**UK7**	209.66	Swab	Throat	COVID	17.71	No tissue
**UK8**	250.34	Swab	Throat	COVID	18.91	No tissue
**UK9**	251.08	Swab	Throat	COVID	20.6	No tissue
**UK10**	257.96	Swab	Throat	COVID	23.86	With tissues

**UK11**	289.25	Swab	Nasopharyngeal Swab	COVID	16.57	No tissue
**UK12**	210.31	Swab	Throat	COVID	18.54	No tissue
**UK13**	189.74	Swab	Throat	COVID	27.84	No tissue
**UK14**	121.50	Swab	Throat	COVID	31.5	No tissue
**UK15**	87.84	Swab	Throat	COVID	34.73	No tissue

Table S3b continued.

Patient ID	Mean Impedance (kΩ)	Specimen type	Specimen source	Target	Ct value	Pureness
**UK16**	222.02	Swab	Throat	COVID	16.88	No tissue
**UK17**	86.81	Swab	Throat	COVID	30.25	No tissue
**UK18**	130.48	Swab	Nasopharyngeal Swab	COVID	23.25	No tissue
**UK19**	179.23	Swab	Throat	COVID	24.26	No tissue
**UK20**	212.73	Swab	Throat	COVID	18.69	No tissue
**UK21**	134.62	Swab	Swab, No Source Indicated	COVID	17.69	Small tissues
**UK22**	166.43	Swab	Nasopharyngeal Swab	COVID	23.36	No tissue
**UK23**	106.11	Swab	Throat	COVID	30.6	No tissue-Very thick like Saliva
**UK24**	93.41	Swab	Throat	COVID	22.56	No tissue
**UK25**	149.44	Swab	Nasopharyngeal Swab	COVID	23.55	No tissue
**UK26**	218.23	Swab	Throat	COVID	15.21	No tissue
**UK27**	219.10	Swab	Throat	COVID	17.81	No tissue
**UK28**	150.20	Swab	Nasopharyngeal Swab	COVID	26.07	No tissue
**UK29**	196.07	Swab	Nasopharyngeal Swab	COVID	23.13	No tissue
**UK30**	122.80	Swab	Throat	COVID	22.62	No tissue
**UK31**	175.31	Swab	Throat	COVID	16.14	With tissues
**UK32**	152.55	Swab	Throat	COVID	20.31	No tissue
**UK33**	133.66	Swab	Throat	COVID	27.66	No tissue
**UK34**	147.92	Swab	Throat	COVID	22.7	No tissue
**UK35**	206.95	Swab	Throat	COVID	16.54	No tissue

S5-1: Clinical samples analysis

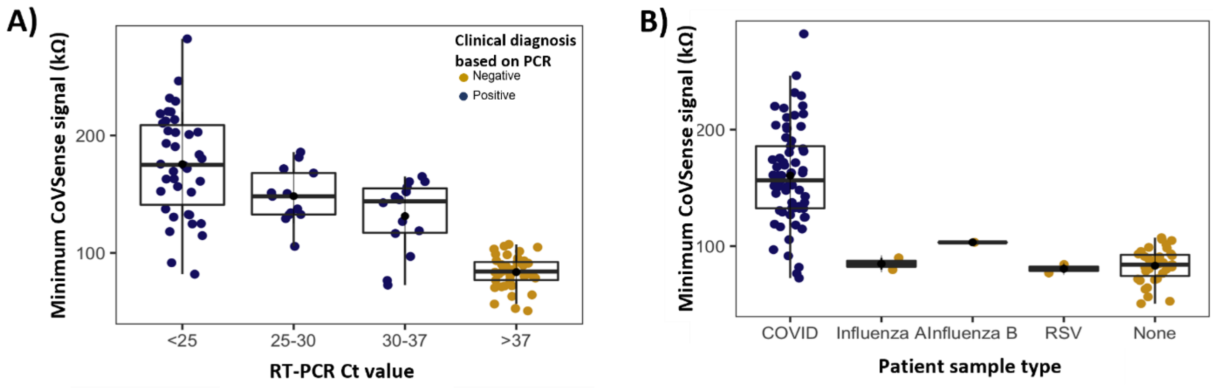

Figure S5. Clinical cohort measurements for infection detection with CoVSense and real-time reverse transcription-polymerase chain reaction (RT-PCR). (A) Minimum CoVSense impedance measurements in relation to RT-PCR Ct value groups within the cohort. **(B)** Minimum CoVSense impedance measurements in SARS-CoV-2 patients compared to patients with other viral upper respiratory infections. The annotations of Figure A are applicable to Figure B.

S5-2: Performance metrics of CoVSense based on the virus variant and the genders

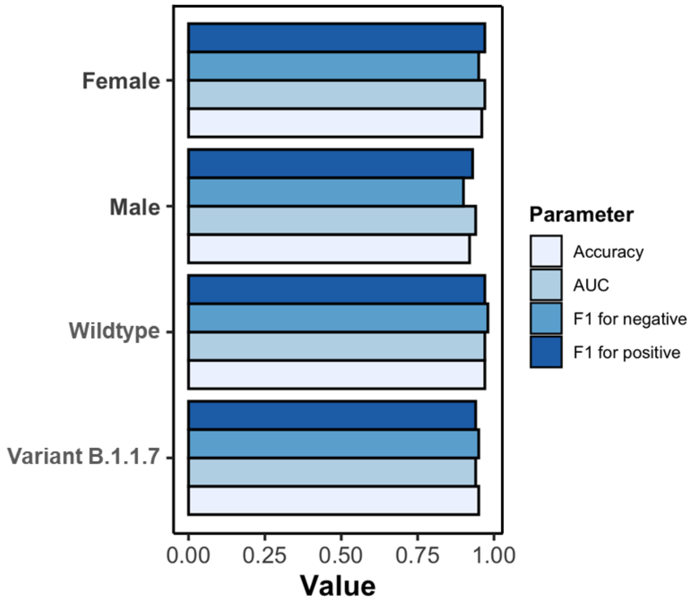

Figure S6. Performance metrics of CoVSense based on the virus variant and the genders.

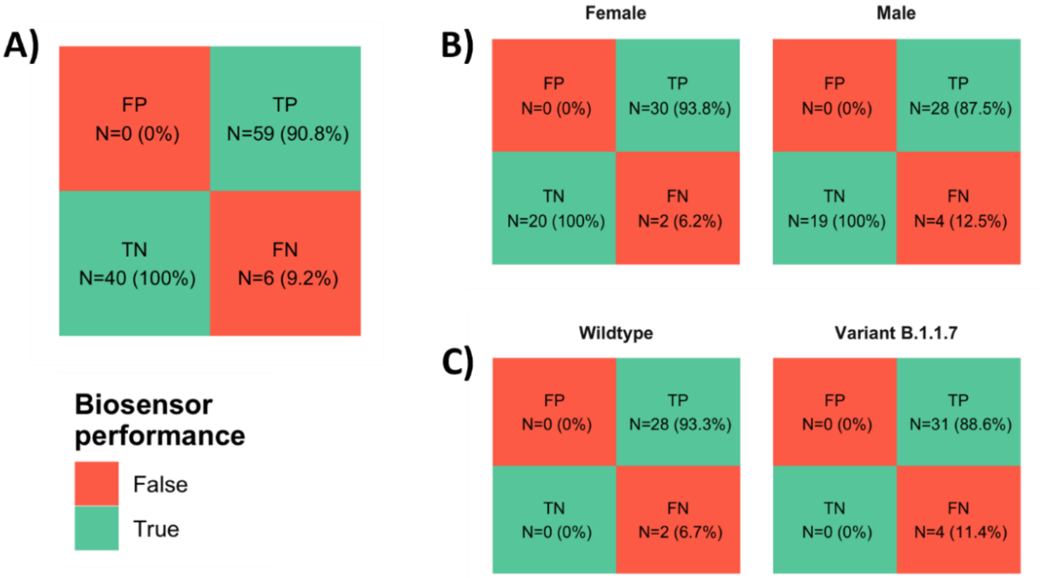

Figure S7. Clinical performance of CoVSense. **(A)** Sensitivity and specificity of the clinical samples measured by the CoVSense for all 105 clinical samples with 37 < Ct <15. **(B)** Clinical sensitivity and specificity of the clinical samples measured by the CoVSense based on the genders and the variant detected. **(C)** The clinical sensitivity and specificity of the measurements of SARS-CoV-2 wildtype and variant B.1.1.7 samples (FP: false-positive, FN: false-negative, TP: true-positive, and TN: true-negative).

Table S4. CoVSense diagnostic performance in compared to PCR as a gold standard and Abbott Panbio.

Sample ID	Patient ID	RT-PCR	Panbio	CoVSense
**S1**	Naso 1	NEG	NEG	NEG
**S2**	Naso 2	NEG	NEG	NEG
**S3**	Naso 3	NEG	NEG	NEG
**S4**	Naso 4	NEG	NEG	NEG
**S5**	Naso 5	NEG	NEG	NEG
**S6**	Naso 6	NEG	NEG	NEG
**S7**	Naso 7	NEG	NEG	NEG
**S8**	Naso 8	NEG	NEG	NEG
**S9**	Naso 9	NEG	NEG	NEG
**S10**	Naso 10	NEG	NEG	NEG
**S11**	Naso 11	NEG	NEG	NEG
**S12**	Naso 12	NEG	NEG	NEG
**S13**	Naso 13	NEG	NEG	NEG
**S14**	Naso 14	NEG	NEG	NEG
**S15**	Naso 15	NEG	NEG	NEG
**S16**	Naso 16	NEG	NEG	NEG
**S17**	Naso 17	NEG	NEG	NEG
**S18**	Naso 18	NEG	NEG	NEG
**S19**	Naso 19	NEG	NEG	NEG
**S20**	Naso 20	NEG	NEG	NEG

Table S4 continued.

Sample ID	Patient ID	RT-PCR	Panbio	CoVSense
**S21**	Naso 21	NEG	NEG	NEG
**S22**	Naso 22	NEG	NEG	NEG
**S23**	Naso 23	NEG	NEG	NEG
**S24**	Naso 24	NEG	NEG	NEG
**S25**	Naso 25	NEG	NEG	NEG
**S26**	Naso 26	NEG	NEG	NEG
**S27**	Naso 27	NEG	NEG	NEG
**S28**	Naso 28	NEG	NEG	NEG
**S29**	Naso 29	NEG	NEG	NEG
**S30**	Naso 30	NEG	NEG	NEG
**S31**	Naso 31	NEG	NEG	NEG
**S32**	Naso 32	NEG	NEG	NEG
**S33**	Naso 33	NEG	NEG	NEG
**S34**	Naso 34	NEG	NEG	NEG
**S35**	Naso 35	NEG	NEG	NEG
**S36**	Naso 36	NEG	NEG	NEG
**S37**	Naso 37	NEG	NEG	NEG
**S38**	Naso 38	NEG	NEG	NEG
**S39**	Naso 39	NEG	NEG	NEG
**S40**	Naso 40	NEG	NEG	NEG
**S41**	1	POS	POS	POS
**S42**	2	POS	POS	POS
**S43**	3	POS	POS	POS
**S44**	4	POS	POS	NEG
**S45**	5	POS	POS	POS
**S46**	6	POS	POS	POS
**S47**	7	POS	POS	POS
**S48**	8	POS	POS	POS
**S49**	9	POS	POS	POS
**S50**	10	POS	POS	POS
**S51**	11	POS	NEG	POS
**S52**	12	POS	POS	POS
**S53**	13	POS	NEG	POS
**S54**	14	POS	NEG	POS
**S55**	15	POS	POS	POS
**S56**	16	POS	POS	POS
**S57**	17	POS	POS	POS
**S58**	18	POS	NEG	NEG
**S59**	19	POS	NEG	POS

Table S4 continued.

Sample ID	Patient ID	RT-PCR	Panbio	CoVSense
**S60**	20	POS	POS	POS
**S61**	21	POS	NEG	POS
**S62**	22	POS	NEG	POS
**S63**	23	POS	NEG	POS
**S64**	24	POS	NEG	POS
**S65**	25	POS	NEG	POS
**S66**	26	POS	NEG	POS
**S67**	27	POS	NEG	POS
**S68**	28	POS	NEG	POS
**S69**	29	POS	NEG	POS
**S70**	30	POS	NEG	POS
**S71**	UK1	POS	POS	POS
**S72**	UK2	POS	POS	POS
**S73**	UK3	POS	POS	POS
**S74**	UK4	POS	POS	POS
**S75**	UK5	POS	POS	POS
**S76**	UK6	POS	POS	POS
**S77**	UK7	POS	POS	POS
**S78**	UK8	POS	POS	POS
**S79**	UK9	POS	POS	POS
**S80**	UK10	POS	NEG	POS
**S81**	UK11	POS	POS	POS
**S82**	UK12	POS	POS	POS
**S83**	UK13	POS	NEG	POS
**S84**	UK14	POS	POS	POS
**S85**	UK15	POS	NEG	NEG
**S86**	UK16	POS	NEG	POS
**S87**	UK17	POS	POS	NEG
**S88**	UK18	POS	POS	POS
**S89**	UK19	POS	POS	POS
**S90**	UK20	POS	POS	POS
**S91**	UK21	POS	POS	POS
**S92**	UK22	POS	POS	POS
**S93**	UK23	POS	NEG	NEG
**S94**	UK24	POS	NEG	NEG
**S95**	UK25	POS	POS	POS
**S96**	UK26	POS	POS	POS
**S97**	UK27	POS	POS	POS
**S98**	UK28	POS	POS	POS

Table S4 continued.

Sample ID	Patient ID	RT-PCR	Panbio	CoVSense
**S99**	UK29	POS	POS	POS
**S100**	UK30	POS	POS	POS
**S101**	UK31	POS	POS	POS
**S102**	UK32	POS	POS	POS
**S103**	UK33	POS	NEG	POS
**S104**	UK34	POS	POS	POS
**S105**	UK35	POS	POS	POS

NEG: Negative; POS: Positive

S6. The handheld impedance spectroscopy system

S6-1. Principle of impedance measurement

In the low complexity potentiostat readout system developed and used in this work, the impedance is measured based on equations (1) and (2).

$$Z_{\text{Amplitude}} = \alpha \times \frac{A}{B} \tag{1}$$

$$Z_{phase} = \frac{T}{P} \times \frac{\pi}{180} \tag{2}$$

where α represents the gain related to the implemented circuit, P is the period of the excitation or response signals seen in **Figure S8**. A and B are amplitudes of excitation and response signals. The required sinusoidal excitation signal is created by the microcontroller using a 10 bits DAC channel. The response signal is the signal recorded by the microcontroller using a 10 bits ADC channel.

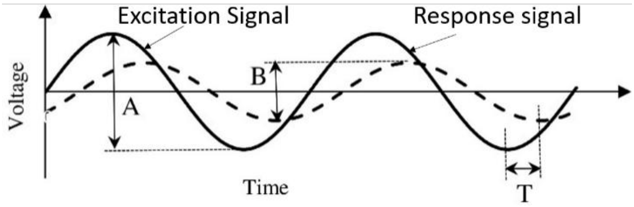

Figure S8. Illustration of excitation and response signals.

S6-2. Potentiostat readout system

The handheld potentiostat readout system developed and used in this work consists of a microcontroller unit, an analog front-end circuitry, and a graphical user interface (GUI) unit as described below in the block diagram shown in **Figure S9**.

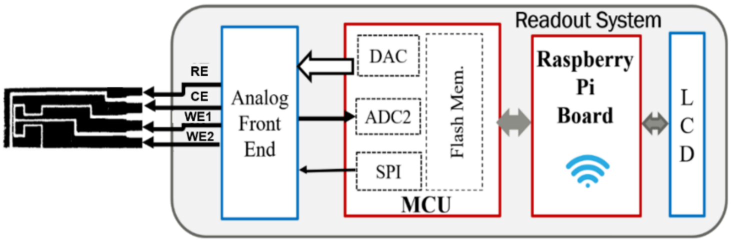

Figure S9. Overview of the block diagram of the handheld potentiostat system.

S6-2-1. Microcontroller Unit

The microcontroller unit (MCU) breakout is the Arduino DUE with an ARM Cortex-M3 32-bit processor (64 MHz, two 256 kbytes embedded Flash, 128-bit wide access, memory accelerator, dual bank 100 (64+32) Kbytes embedded SRAM with dual banks). The USB interface allows for programming the MCU, powering the unit, and communicating through UART. The breakout can also be powered by a Lithium-Polymer (LiPo) battery and charged using the same USB interface. The firmware was developed using Arduino IDE.

The firmware of the MC was developed in C program to control the digitally controlled resistors in the analog front-end circuitry and generate excitation and record response sinusoidal signals. To decrease the error, the excitation and output sinusoidal signals were smoothened using a moving average operator of the buffer size of 11 samples. To further suppress the effect of noise, each measurement was repeated 20 times and the output signal was obtained by averaging the 20 measurements.

S5-2-2. Analog Front End circuitry

The Analog Front End circuitry consists of Low Pass Filter (LPF), Voltage Attenuator, and Potentiostat as described and demonstrated below.

Low-pass filter. A Sallen-Key LPF with variable cut-off frequency [1] was developed with two digitally controlled resistors via SPI serial interface. The microcontroller enables tuning the filter at seven different frequencies of 1, 3, 11, 37, 125, 412, and 1390 Hz.

Voltage attenuator. The excitation signal is a sinusoidal signal with an amplitude of 10 mV peak-to-peak. Therefore, to generate a small signal voltage from the output signal of LPF, a voltage attenuator (1/20) was developed using an inverting operational amplifier (OPAMP).

Potentiostat. A standard potentiostat circuitry consisting of three OPAMPs was employed as shown in **Figure 10**. The digitally controlled feedback resistor (FB) via SPI serial interface enables the operation of the impedance measurement system in seven different ranges. A switch (SW) is used to select the first and second working electrodes (WE1 or WE2).

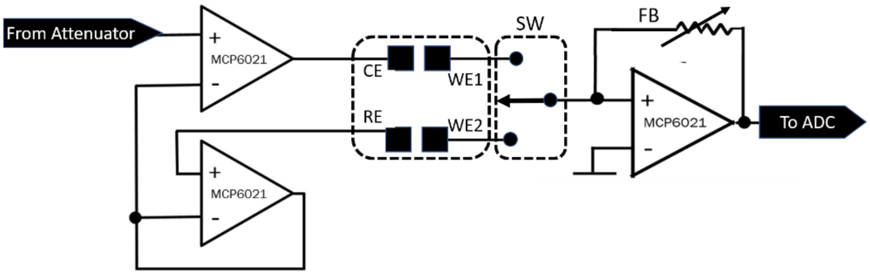

Figure S10. The circuit diagram of the implemented three-electrode potentiostat.

Printed circuit board design and implementation. The analog front was developed in a PCB using Altium Designer software with the layout shown in **Figure S11**. In this layout, the PCB ground plane was split into an analog plane (AGND) and a digital plane (DGND) to reduce the noise coupled from digital circuits into the analog signals. Analog and digital components were separated and their corresponding signal traces were placed right above their respective ground planes. In this way, the signals return to their ground planes by vias. This PCB was manufactured by www.seeedstudio.com (**Figure S12**). A list of elements used for fabricating this PCB board is presented in **Table S5**.

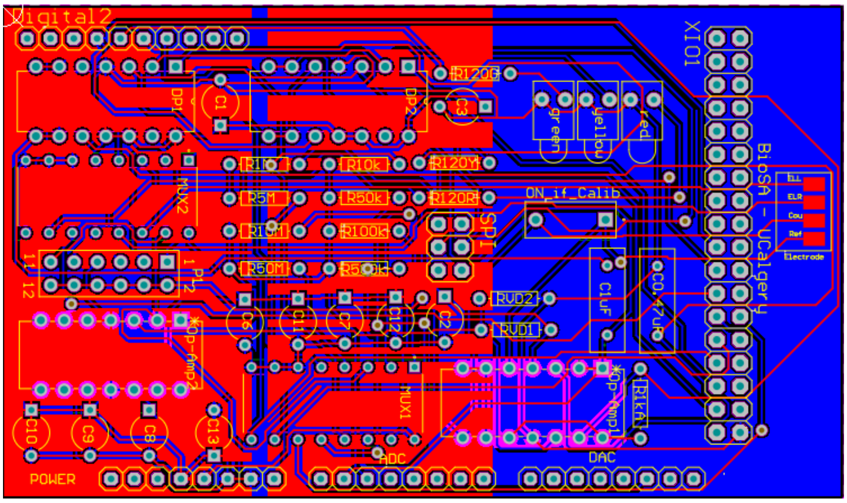

Figure S11. The final layout of the printed circuit board (PCB) for the potentiostat circuit.

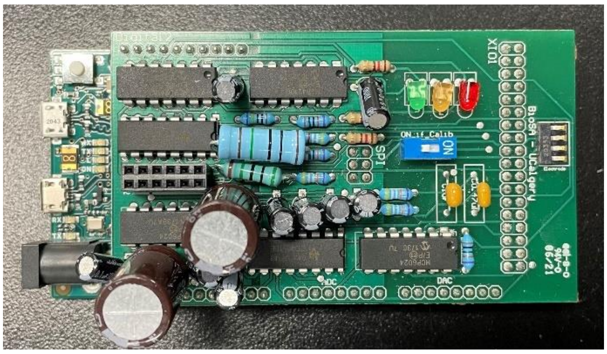

Figure S12. The final PCB of the readout interface. The female header connectors on the left are the electrode

sockets.

Table S5. The list of electronic components for the potentiostat readout system.

Designator	Supplier part number	Description	Manufacturer	Quantity	Unit price (USD)
**Op-Amp1, Op-Amp2**	MCP6024-E/P	IC OPAMP GP 4 CIRCUIT 14DIP	Microchip Technology	2	2.25
**DP1, DP2**	MCP42100-E/P	IC DGT POT 100KOHM 256TAP 14DIP	Microchip Technology	2	2.72
**MUX1, MUX2**	CD4051BE	IC MUX/DEMUX 8X1 16DIP	Texas Instruments	2	0.67
**R10k**	YR1B10KCC	RES 10.0K OHM 1/4W 0.1% AXIAL	TE Connectivity Passive Product	1	0.81
**R50k**	YR1B49K9CC	RES 49.9K OHM 1/4W 0.1% AXIAL	TE Connectivity Passive Product	1	0.81
**R100k**	YR1B100KCC	RES 100K OHM 1/4W 0.1% AXIAL	TE Connectivity Passive Product	1	0.81
**R500K, RVD1, RVD2**	YR1B499KCC	RES 499K OHM 1/4W 0.1% AXIAL	TE Connectivity Passive Product	3	0.81
**R1M**	YR1B1M0CC	RES 1.00M OHM 1/4W 0.1% AXIAL	TE Connectivity Passive Product	1	0.81
**R5M**	RNF14FAD4M75	RES 4.75M OHM 1/4W 1% AXIAL	Stackpole Electronics Inc	1	0.14
**R10M**	VR68000001005FAC00	RES 10M OHM 1W 1% AXIAL	Vishay Beyschlag/Draloric/ BC Components	1	1.05
**R50M**	HVA12FA50M0	RES 50M OHM 1.2W 1% AXIAL	Stackpole Electronics Inc	1	0.74
**R120Y, R120R, R120G**	CFR-25JB-52-120R	RES 120 OHM 1/4W 5% AXIAL	Yageo	3	0.12
**C1, C2, C3, C7, C8, C10, C11, C12, C13**	UPS1H010MDD1TD	CAP ALUM 0.1UF 20% 100V RADIAL	Nichicon	9	0.26
**C6, C9**	UCY2H100MHD1TO	CAP ALUM 10UF 20% 500V RADIAL	Nichicon	2	1.90
**C0.47uF**	C330C474K5R5TA	CAP CER 0.47UF 50V X7R RADIAL	KEMET	1	0.48
**C1uF**	C330C105J5R5TA	CAP CER 1UF 5% 50V X7R RADIAL	KEMET	1	1.86
**P1**	PPTC062LFBN-RC	12 Position Header Connector Through Hole	Sullins Connector Solutions	1	0.87

**Electrode**	472861001	4 Position Spring Battery Contact Connector Surface Mount	Molex	1	0.69
**Digital2**	PEC10SABN	Connector Header Through Hole 10 position 0.100" (2.54mm)	Sullins Connector Solutions	1	0.96
**DAC, ADC, POWER**	PEC08SABN	Connector Header Through Hole 8 position 0.100" (2.54mm)	Sullins Connector Solutions	3	0.85
**XI01**	PEC18DABN	Connector Header Through Hole 36 position 0.100" (2.54mm)	Sullins Connector Solutions	1	2.45
**SPI**	PPPC032LFBN-RC	6 Position Header Connector Through Hole	Sullins Connector Solutions	1	0.69
**red**	SLR-343VCT32	Red LED Indication - Discrete 2V Radial	Rohm Semiconductor	1	0.62
**TOTAL**				45	**23.37**

S6-2-3. Graphical user interface

A Raspberry pi board connected to a 3.5-inch touchscreen LCD was used as an easy and friendly user interface, with the capability of wireless internet and Cloud storage. The Raspberry pi computer communicates with the EIS readout system through a serial port and controls the LCD via GPIO pins. A python-based App was developed and run under a customized Raspbian OS to make data collection, storage, and analysis easier, while the GUI provides instructions to collect the sample, run the test, and show the results.

S6-2-4. Full readout system

The readout system consists of a developed PCB board incorporated with Arduino and Raspberry pi boards and LCD and as seen in **Figure S13**.

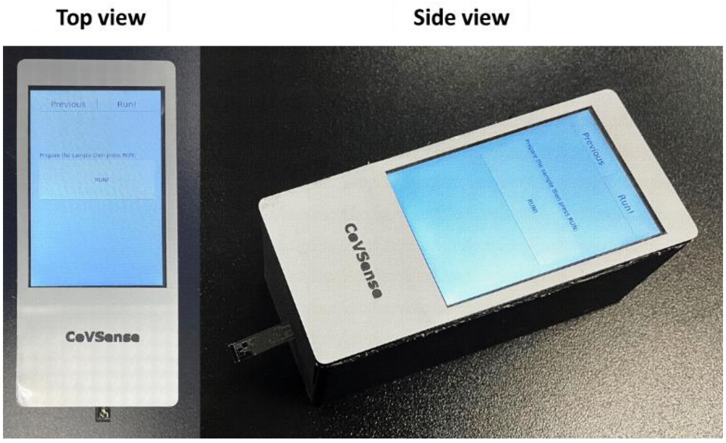

Figure S13. The integration of developed PCB and other boards and the fully packaged readout system.

S6-3. Characterization of the proposed handheld reader

S6-3-1. Testing the handheld impedimetric reader with known Resistor and capacitor values

For the detection of COVID-19 patients, measurement of the impedance in five different frequencies

ranging logarithmically from 1 Hz to 1.4 kHz was proven to be sufficient based on the tests of the immunosensor with Metrohm Autolab measurement device. The results were then validated against the Autolab PGSTAT204 (Metrohm), with FRA32M electrochemical impedance spectroscopy (EIS) module and NOVA software. These frequencies were observed to be sufficient to uncover the impedance difference between the SARS-CoV-2 positive and negative clinical samples. Functionality assessment of the handheld reader was performed by testing them in the selected logarithmically-spaced frequencies. The functionality assessment was performed using the known resistors and capacitors combination (**Figure S14**) by repeating each experiment 5 times. The repeatability was significant with only 1% variation among all the runs. As a result, the worst-case error between the measurements using the proposed readout system and the AutoLab device as an industrial and research-grade tool was below 20% in some frequencies.

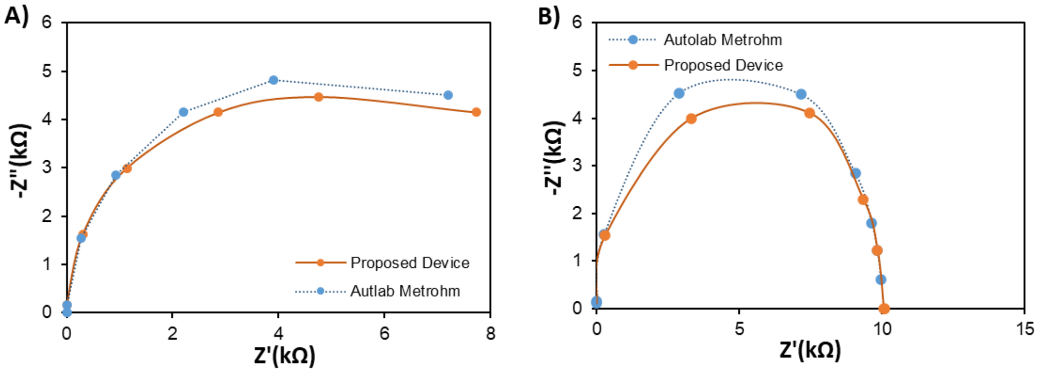

Figure S14. Test comparison between the proposed handheld reader and AutoLab for the known resistor and capacitor combinations; **(A)** a resistor (10 kΩ) in parallel with a capacitor (10 µF), **(B)** a resistor (10 kΩ) in parallel with a capacitor (1µF). Measured frequencies are 1, 3, 5, 10, 10, 30, and 1400 Hz.

S6-3-2. Redox testing of the proposed reader

The tests with the redox solution were performed first by sweeping the frequencies in the left electrode (L), followed by the right electrode (R). The results show similarity in high frequencies with more errors in low frequencies. The higher errors at lower frequencies are attributed to the limited phase measure resolution to the number of ADC values and the time-based phase measurement (**Figure S15**).

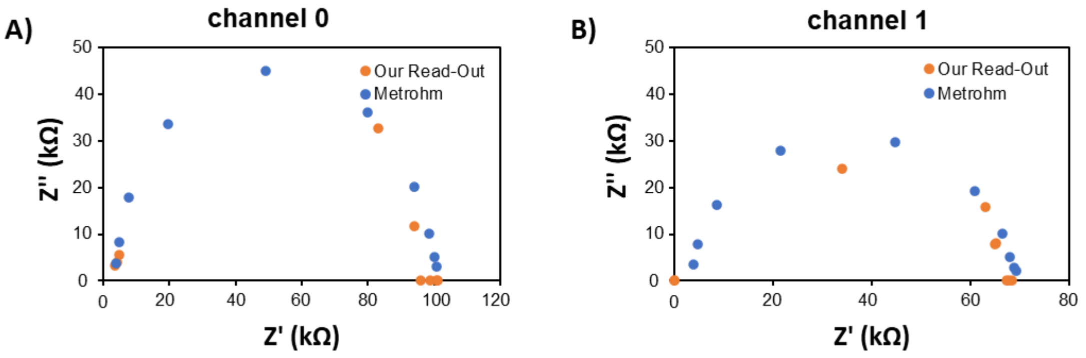

Figure S15. Test comparison of working electrodes, measured frequency 1, 3, 11, 37, 125, 412, and 1390 Hz; **(A)** data obtained from measurement of the left working electrode (L), and **(B)** the right working electrode (R).

S6-4. Comparison of the handheld potentiostat reader and the state-of-the-art potentiostat

We addressed the challenge of developing a low-cost but highly accurate impedimetric-based readout system using low complexity, yet programmable system suitable for performing an efficient calibration. The electrical characterization and biochemical testing results proved the functionality, sensitivity, and selectivity of this reader. The proposed low-cost readout system was successful in detecting COVID-19-positive patients. **Table S6** compares the performance of our potentiostat reader once used in combination with our biosensor and other state-of-the-art electrochemical biosensors used for the detection of different SARS-CoV-2 biomarkers. The key benefits of our reader are its cost-effectiveness, fast measurement time, and the capability of transmitting the data to the Cloud.

Table S6. Impedimetric biosensors developed for the detection of various SARS-CoV-2 biomarkers.

Dedicated Readout	Portable Read-out	Readout price (USD)	Measurement time (min)	Data to Cloud	Target	BRE	Surface modification	Sample	Ref.
NO	Yes	>800	5	NO	S protein	Ab	Graphene/carbon ink	Nasopharyngeal fluid	[2]
NO	Yes	>800	>30	NO	N and S genes	DNA	SiNPs<br>PAA<br>PSS	Clinical	[3]
NO	NO	>10000	>30	NO	IgG, IgM	DNA	-	Human serum	[4]
NO	NO	-	>50	NO	HEV	Ab	GQDs@AuNP-PAni	Fecal (monkey)	[5]
NO	NO	-	21	NO	S-protein	ACE2	Palladium	OEC-M1	[6]
Yes	Yes	>200	10	NO	SARS-CoV-2 (spike proteins)	Ab	SPCE	Oro/nasopharyngeal fluid and saliva samples	[7]
**Yes**	**Yes**	**<25**	**4**	**Yes**	**SARS-CoV-2 N-protein**	**Ab**	**Engineered Graphene@PEDOT:PSS/ carbon**	**Nasopharyngeal and throat Swabs**	**This work**

HEV: Hepatitis E virus, GQDs@AuNP-PAni: graphene quantum dots and gold-embedded polyaniline nanowires, AIV: avian influenza

virus, IDA: Interdigitated array, SiNPs: silica nanoparticles, PAA: poly(allylamine)hydrochloride, PSS: poly(sodium 4-styrene) sulfonate, ACE2: angiotensin-converting enzyme 2, OEC-M1: human oral cavity squamous carcinoma cells.

References

[1] Z. Hank, Linear circuit design handbook. Chapter: **2008**.

[2] M. A. Ehsan, S. A. Khan, A. Rehman, *Diagnostics (Basel)* **2021**, *11* (6), https://doi.org/10.3390/diagnostics11061030.

[3] P. Chandra, *Sensors International* **2020**, *1*, 100019, https://doi.org/10.1016/j.sintl.2020.100019.

[4] M. Labib, A. S. Zamay, D. Muharemagic, A. V. Chechik, J. C. Bell, M. V. Berezovski, *Anal Chem* **2012**, *84* (4), 1813, https://doi.org/10.1021/ac203412m.

[5] A. D. Chowdhury, K. Takemura, T.-C. Li, T. Suzuki, E. Y. Park, *Nature communications* **2019**, *10* (1), https://doi.org/10.1038/s41467-019-11644-5.

[6] Xiaohong Wang, Zhuo Zhao, Yuhe Wang, Yuhe Wang, Jianhan Lin, J. Lin., presented at *International Conference on Computer and Computing Technologies in Agriculture*, **2015**.

[7] S. A. Perdomo, V. Ortega, A. Jaramillo-Botero, N. Mancilla, J. H. Mosquera-DeLaCruz, D. P. Valencia, M. Quimbaya, J. D. Contreras, G. E. Velez, O. A. Loaiza, A. Gómez, J. de la Roche, *IEEE Transactions on Instrumentation and Measurement* **2021**, *70*, 1, https://doi.org/10.1109/TIM.2021.3119147.
