# Supplementary material for: CoVSense: Ultrasensitive Nucleocapsid Antigen Immunosensor for Rapid Clinical Detection of Wildtype and Variant SARS‐CoV‐2
Source: Adv Sci (Weinh). 2023 Mar 30;10(15):2206615. doi: 10.1002/advs.202206615 (PMC10214237; doi:10.1002/advs.202206615)
Supplement: Supplementary file 2 — Supporting Information [file ADVS-10-2206615-s001.zip › SI_File_1_CoVSense_Clinical testing_Advanced Science.html]

CoVSense: Ultrasensitive nucleocapsid antigen immunosensor for rapid clinical detection of wildtype and variant SARS-CoV-2


# CoVSense: Ultrasensitive nucleocapsid antigen immunosensor for rapid clinical detection of wildtype and variant SARS-CoV-2

#### Razieh Salahandish, Jae Eun Hyun, Fatemeh Haghayegh, Hamed Osouli Tabrizi, Shirin Moossavi, Sultan Khetani, GiancarloAyala-Charca, Byron M. Berenger, Yan Dong Niu, Ebrahim Ghafar-Zadeh, Amir Sanati Nezhad

#### 2022-06-02

```
library(tidyverse)
library(tidymodels)
library(epiR)
library(ggrepel)
library(broom)
```

# 1 **Functions**

```
# produce summary statistics (mean and +/- sd) 
data_summary <- function(x) {
   m <- mean(x, na.rm = TRUE)
   ymin <- m-sd(x)
   ymax <- m+sd(x)
   return(c(y=m,ymin=ymin,ymax=ymax))
}
```

```
# minmax normalisation
normalize <- function(x, na.rm = TRUE) {
    return((x- min(x)) /(max(x)-min(x)))
}
```

```
# reverse of minmax for CT value
# we want the lowest CT value to be considered the highest value in the concordance analysis

normalize2 <- function(x, na.rm = TRUE) {
    return((max(x)- x) /(max(x)-min(x)))
}
```

# 2 **Study design**

Recruitment flow chart, intention to treat

# 3 **Characterization**

## 3.1 Figure 1B

Electrochemical impedance spectroscopy (EIS) signals recorded by the working electrode-1 (WE-1) and working electrode-2 (WE-2) of CoVSense for different surface modification steps, including antibody immobilization

```
df <- read.table("AbsoluteEIS.txt", 
                 header=TRUE, 
                 sep="\t", 
                 na.strings="NA", 
                 dec=".", 
                 strip.white=TRUE) %>%
  rename("Electrode surface"=Electrode_surface)
  

df
```

```
df$`Electrode surface` <- factor(df$`Electrode surface`, levels = c("Bare", "Antibody", "BSA", "UTM media"))
```

```
fig <- ggplot(df, aes(`Electrode surface`, Measurement, color=Electrode))+
  geom_jitter(position=position_jitter(0.2),  size=1.2, color="goldenrod") + 
  stat_summary(fun= mean, geom="line", aes(group=Electrode), color="grey") +
  stat_summary(fun=mean, geom="point", color="black", size=1.5, aes(group=Electrode))+
  ylab("CoVSense signal (kΩ)")+
  theme_bw()+
  theme(panel.grid.major = element_blank(), panel.grid.minor = element_blank())+
  facet_wrap(~Electrode)+
  theme(plot.title = element_text(size=10, hjust = 0.5, face = "bold"),
        axis.title = element_text(face = "bold", size = 12),
        legend.title = element_text(face = "bold"), 
        legend.text.align = 0,
        strip.text.x = element_text(face = "bold", size = 12),
        axis.text.x = element_text(angle = 45, hjust = 1))
fig
```

## 3.2 Figure 1D

Biosensor’s feasibility study with 20 clinical samples, 10 controls, and 10 B.1.1.7 variant positive (+ve) samples measured with CoVSense on both working electrodes

```
df <- read.table("EIS_Nyquist_plots.txt", 
                 header=TRUE, 
                 sep="\t", 
                 na.strings="NA", 
                 dec=".", 
                 strip.white=TRUE) %>%
  gather(R1:R3, key=Replicate, value=Measurement)
  

df
```

```
fig <- ggplot(df, aes(reorder(Sample, Rank), Measurement, color=Electrode))+
  geom_jitter(position=position_jitter(0.2),  size=1.2) + 
  geom_boxplot(alpha=0.1)+
  theme_bw()+
  theme(panel.grid.major = element_blank(), panel.grid.minor = element_blank())+
  ylab("CoVSense signal (kΩ)")+
  xlab("Patient sample #")+
  theme(plot.title = element_text(size=10, hjust = 0.5, face = "bold"),
        axis.title = element_text(face = "bold", size = 12),
        legend.title = element_text(face = "bold"), 
        legend.text.align = 0,
        axis.text.x = element_text(angle = 45, hjust = 1),
        strip.text.x = element_text(face = "bold", size = 12))+
  geom_hline(yintercept = 121.02, linetype = "dashed", color="#B35806")+
  theme(legend.position = "bottom")+
  scale_color_manual( values=c("dodgerblue4",  "goldenrod"))
fig
```

## 3.3 Figure 1E

Optimal incubation period of the clinical sample on the biosensor required for detecting N-protein in nasopharyngeal (NP) samples. incubation

```
df <- read.table("incubation_time.txt", 
                 header=TRUE, 
                 sep="\t", 
                 na.strings="NA", 
                 dec=".", 
                 strip.white=TRUE) 
  

df
```

```
df$Time <- factor(df$Time)
```

```
fig <- ggplot(df, aes(Time, Measurement))+
  geom_jitter(position=position_jitter(0.2),  size=1.2) + 
  geom_boxplot(alpha=0.1)+
  ylab("CoVSense signal (kΩ)")+
  xlab("Time (min)")+
  theme_bw()+
  theme(panel.grid.major = element_blank(), panel.grid.minor = element_blank())+
  theme(plot.title = element_text(size=10, hjust = 0.5, face = "bold"),
        axis.title = element_text(face = "bold", size = 12),
        legend.title = element_text(face = "bold"), 
        legend.text.align = 0,
        strip.text.x = element_text(face = "bold", size = 12))+
  geom_hline(yintercept = 121.02, linetype = "dashed", color="#B35806")+
   theme(legend.position = "none")
fig
```

## 3.4 Figure 1F

Up to 1000X diluted samples can be reliably detected by CoVSense when two clinical +ve samples #13 and #15 diluted between 1X-10,000X were measured for their N-proteins

```
df <- read.table("dilution.txt", 
                 header=TRUE, 
                 sep="\t", 
                 na.strings="NA", 
                 dec=".", 
                 strip.white=TRUE) 
  

df
```

```
fig <- ggplot(df, aes(Dilution, Measurement))+
  geom_jitter(position=position_jitter(0.2), color="goldenrod", size=1.2) + 
  stat_summary(fun= mean, geom="line", aes(group=Patient), color = "grey") +
  stat_summary(fun=mean, geom="point", color="black", size=1.5, aes(group=Patient))+
  theme_bw()+
  theme(panel.grid.major = element_blank(), panel.grid.minor = element_blank())+
  facet_wrap(~Patient)+
  scale_x_log10()+
  ylab("CoVSense signal (kΩ)")+
  xlab("Dilution rate")+
  theme(plot.title = element_text(size=10, hjust = 0.5, face = "bold"),
        axis.title = element_text(face = "bold", size = 12),
        legend.title = element_text(face = "bold"), 
        legend.text.align = 0,
        strip.text.x = element_text(face = "bold", size = 12))+
  geom_hline(yintercept = 121.02, linetype = "dashed", color="#B35806")
fig
```

## 3.5 Figure 1G

Selective response of CoVSense recorded when the biosensor was incubated with samples containing Influenza A (Flu-A), Influenza B (Flu-B), respiratory syncytial virus (RSV) and SARS-CoV-2 +ve (sample #13), and their mixture with sample #13

```
df <- read.table("selectivity.txt", 
                 header=TRUE, 
                 sep="\t", 
                 na.strings="NA", 
                 dec=".", 
                 strip.white=TRUE) 
  

df
```

```
df$Sample <- factor(df$Sample, levels = c("BSA (Blank control)", "FLUA", "FLUB", "RSV", "P13 (positive)", "P13+FLUA" ,"P13+FLUB", "P13+RSV",      "P13+FLUA+FLUB+RSV" ))
```

```
fig <- ggplot(df, aes(Sample, Measurement, color=COVID))+
  geom_jitter(position=position_jitter(0.2),  size=1.2) + 
  geom_boxplot(alpha=0.1)+
  theme_bw()+
  theme(panel.grid.major = element_blank(), panel.grid.minor = element_blank())+
  ylab("CoVSense signal (kΩ)")+
  xlab("Patient sample")+
  theme(plot.title = element_text(size=10, hjust = 0.5, face = "bold"),
        axis.title = element_text(face = "bold", size = 12),
        legend.title = element_text(face = "bold"), 
        legend.text.align = 0,
        axis.text.x = element_text(angle = 45, hjust = 1),
        strip.text.x = element_text(face = "bold", size = 12))+
  geom_hline(yintercept = 121.02, linetype = "dashed", color="grey")+
   theme(legend.position = "bottom")+
  scale_color_manual(name= "SARS-CoV-2\ninfection", values=c("goldenrod3",  "midnightblue"))
fig
```

```
mod <- aov(Measurement ~ Sample, data = df)
summary(mod)
```

```
##             Df Sum Sq Mean Sq F value   Pr(>F)    
## Sample       8  49437    6180   116.2 6.74e-14 ***
## Residuals   18    957      53                     
## ---
## Signif. codes:  0 '***' 0.001 '**' 0.01 '*' 0.05 '.' 0.1 ' ' 1
```

```
TukeyHSD(mod)
```

```
##   Tukey multiple comparisons of means
##     95% family-wise confidence level
## 
## Fit: aov(formula = Measurement ~ Sample, data = df)
## 
## $Sample
##                                             diff       lwr        upr     p adj
## FLUA-BSA (Blank control)              -10.410000 -31.27567  10.455675 0.7120908
## FLUB-BSA (Blank control)               -1.213333 -22.07901  19.652342 0.9999998
## RSV-BSA (Blank control)               -24.316667 -45.18234  -3.450992 0.0156087
## P13 (positive)-BSA (Blank control)     83.833333  62.96766 104.699008 0.0000000
## P13+FLUA-BSA (Blank control)           70.566667  49.70099  91.432342 0.0000000
## P13+FLUB-BSA (Blank control)           77.030000  56.16433  97.895675 0.0000000
## P13+RSV-BSA (Blank control)            76.990000  56.12433  97.855675 0.0000000
## P13+FLUA+FLUB+RSV-BSA (Blank control)  70.646667  49.78099  91.512342 0.0000000
## FLUB-FLUA                               9.196667 -11.66901  30.062342 0.8209208
## RSV-FLUA                              -13.906667 -34.77234   6.959008 0.3722815
## P13 (positive)-FLUA                    94.243333  73.37766 115.109008 0.0000000
## P13+FLUA-FLUA                          80.976667  60.11099 101.842342 0.0000000
## P13+FLUB-FLUA                          87.440000  66.57433 108.305675 0.0000000
## P13+RSV-FLUA                           87.400000  66.53433 108.265675 0.0000000
## P13+FLUA+FLUB+RSV-FLUA                 81.056667  60.19099 101.922342 0.0000000
## RSV-FLUB                              -23.103333 -43.96901  -2.237658 0.0236441
## P13 (positive)-FLUB                    85.046667  64.18099 105.912342 0.0000000
## P13+FLUA-FLUB                          71.780000  50.91433  92.645675 0.0000000
## P13+FLUB-FLUB                          78.243333  57.37766  99.109008 0.0000000
## P13+RSV-FLUB                           78.203333  57.33766  99.069008 0.0000000
## P13+FLUA+FLUB+RSV-FLUB                 71.860000  50.99433  92.725675 0.0000000
## P13 (positive)-RSV                    108.150000  87.28433 129.015675 0.0000000
## P13+FLUA-RSV                           94.883333  74.01766 115.749008 0.0000000
## P13+FLUB-RSV                          101.346667  80.48099 122.212342 0.0000000
## P13+RSV-RSV                           101.306667  80.44099 122.172342 0.0000000
## P13+FLUA+FLUB+RSV-RSV                  94.963333  74.09766 115.829008 0.0000000
## P13+FLUA-P13 (positive)               -13.266667 -34.13234   7.599008 0.4294115
## P13+FLUB-P13 (positive)                -6.803333 -27.66901  14.062342 0.9589187
## P13+RSV-P13 (positive)                 -6.843333 -27.70901  14.022342 0.9575646
## P13+FLUA+FLUB+RSV-P13 (positive)      -13.186667 -34.05234   7.679008 0.4368419
## P13+FLUB-P13+FLUA                       6.463333 -14.40234  27.329008 0.9692373
## P13+RSV-P13+FLUA                        6.423333 -14.44234  27.289008 0.9703160
## P13+FLUA+FLUB+RSV-P13+FLUA              0.080000 -20.78567  20.945675 1.0000000
## P13+RSV-P13+FLUB                       -0.040000 -20.90567  20.825675 1.0000000
## P13+FLUA+FLUB+RSV-P13+FLUB             -6.383333 -27.24901  14.482342 0.9713673
## P13+FLUA+FLUB+RSV-P13+RSV              -6.343333 -27.20901  14.522342 0.9723917
```

## 3.6 Figure 1H

CoVSense’s stability remained intact when 30 clinical controls were measured after their exposure to the ambient condition within 3-5 hrs after assay opening.

```
df <- read.table("stability.txt", 
                 header=TRUE, 
                 sep="\t", 
                 na.strings="NA", 
                 dec=".", 
                 strip.white=TRUE)%>%
  mutate(Rank=Sample)%>%
  separate(Rank, into=c("X","Rank"), sep=4)%>%
  select(-X)
  

df
```

```
fig <- ggplot(df, aes(Sample, Measurement, color=Time))+
  geom_jitter(position=position_jitter(0.2),  size=1.2) + 
  geom_boxplot(alpha=0.1)+
  theme_bw()+
  theme(panel.grid.major = element_blank(), panel.grid.minor = element_blank())+
  facet_wrap(~Time, scales = "free_x", nrow = 1)+
  ylab("CoVSense signal (kΩ)")+
  xlab("Patient sample #")+
  theme(plot.title = element_text(size=10, hjust = 0.5, face = "bold"),
        axis.title = element_text(face = "bold", size = 12),
        legend.title = element_text(face = "bold"), 
        legend.text.align = 0,
        axis.text.x = element_text(angle = 45, hjust = 1, size = 5),
        strip.text.x = element_text(face = "bold", size = 12))+
  theme(legend.position = "none")+
  scale_color_manual( values=c("lightblue4" ,"lightcyan3",  "goldenrod", "lightcoral"))
fig
```

# 4 **Clinical deployment and validation**

```
d <- read.table("covid_data.txt", 
                header=TRUE, 
                sep="\t", 
                na.strings="NA", 
                dec=".", 
                strip.white=TRUE) %>%
  mutate(Row.names = Sample_ID)%>%
  arrange(Rank)%>%
  mutate(CT_cat = ifelse(CT_2 <=25, "<25",
                          ifelse(CT_2 <=30, "25-30", 
                                 ifelse(CT_2 <= 37, "30-37", ">37"))))%>%
  column_to_rownames("Sample_ID")%>%
  mutate(Variant = recode(Variant, 
                           #"Wildtype"="Wildtype", 
                            "Variant-B.1.1.7" = "Variant B.1.1.7"))%>%
  mutate(status = recode(status,
                         "Nursing_Home" = "Nursing home"))

d
```

```
d$CT_cat <- factor(d$CT_cat, levels = c("<25", "25-30",  "30-37", ">37" ))
```

```
d%>%
  group_by(Covid_PCR_result)%>%
  summarize_at("Measurement", c(min, max))%>%
   rename(min=fn1, max=fn2)
```

## 4.1 Figure 2A

Impedance recorded for the clinical cohort of 105 patient samples: 65 SARS-CoV-2 positive samples and 40 negative controls. The measurements were done in triplicates. Red points denote the average measurement per person.

```
fig <- ggplot(d, aes(reorder(Patient_ID, -Measurement), Measurement))+
  geom_jitter(aes(color=Covid_PCR_result, shape = Variant), position=position_jitter(0.01)) + 
  stat_summary(fun.data=data_summary, color="red", size=0.08)+
  theme_bw()+
  theme(panel.grid.major = element_blank(), panel.grid.minor = element_blank())+
  xlab("Patients")+
  ylab("CoVSense signal (kΩ)")+
  theme(legend.position = "bottom", legend.direction = "vertical")+
  theme(plot.title = element_text(size=10, hjust = 0.5, face = "bold"),
        axis.title = element_text(face = "bold", size = 12),
        legend.title = element_text(face = "bold", size = 10), 
        legend.text.align = 0,
        axis.text.x = element_blank(),
        axis.ticks.x = element_blank())+
  scale_color_manual(name= "Clinical diagnosis\nbased on PCR", values=c("goldenrod3",  "midnightblue"))+
  geom_hline(yintercept = 121.02, linetype = "dashed", color="goldenrod3")+
  annotate("text", x = 19.5, y = 128, label = "Maxiumum measurement in controls", color = "goldenrod3", size = 3)+
  geom_hline(yintercept = 72.59, linetype = "dashed", color="midnightblue")+
  annotate("text", x = 22, y = 78, label = "Minimum measurement in COVID patients", color = "midnightblue", size = 3)
  
fig
```

```
d_mean <- d%>%
  filter(Measurement != "NA")%>%
  group_by(Patient_ID)%>%
  mutate(Measurement2=mean(Measurement))%>%
  ungroup()%>%
  distinct(Patient_ID, .keep_all=TRUE)
d_mean
```

```
d_mean%>%
  group_by(CT_cat)%>%
  summarize_at("Measurement2", c(mean, sd))%>%
   rename(meam=fn1, sd=fn2)
```

## 4.2 Identifying the optimum cutoff

```
library(OptimalCutpoints)
```

### 4.2.1 Cutoff based on the mean of 3 replicates

```
d2 <- d_mean %>%
   filter(Measurement2 != "NA")%>%
   column_to_rownames("Row.names")
d2
```

```
d2$Covid_PCR_result <- as.factor(d2$Covid_PCR_result)
d2$Covid_PCR_result <- relevel(d2$Covid_PCR_result, ref = "Negative")
```

```
set.seed(678194671)

optimal.cutpoint <- optimal.cutpoints(X = "Measurement" , 
                                      status = "Covid_PCR_result",
                                      tag.healthy = "Negative", 
                                      methods = "Youden",  
                                      data = d2, 
                                      pop.prev = NULL,
                                      control = control.cutpoints(),
                                      ci.fit = FALSE, 
                                      conf.level = 0.95, 
                                      trace = FALSE) 

summary(optimal.cutpoint)
```

```
## 
## Call:
## optimal.cutpoints.default(X = "Measurement", status = "Covid_PCR_result", 
##     tag.healthy = "Negative", methods = "Youden", data = d2, 
##     pop.prev = NULL, control = control.cutpoints(), ci.fit = FALSE, 
##     conf.level = 0.95, trace = FALSE)
## 
## Area under the ROC curve (AUC):  0.966 (0.934, 0.998) 
## 
## CRITERION: Youden
## Number of optimal cutoffs: 1
## 
##                      Estimate
## cutoff            118.8500000
## Se                  0.8769231
## Sp                  1.0000000
## PPV                 1.0000000
## NPV                 0.8333333
## DLR.Positive              Inf
## DLR.Negative        0.1230769
## FP                  0.0000000
## FN                  8.0000000
## Optimal criterion   0.8769231
```

### 4.2.2 Cutoff based on the minimum of 3 replicates

```
d_min <- d%>%
  filter(Measurement != "NA")%>%
  group_by(Patient_ID)%>%
  mutate(Measurement2=min(Measurement))%>%
  ungroup()%>%
  distinct(Patient_ID, .keep_all=TRUE)
d_min
```

```
d4 <- d_min %>%
   filter(Measurement2 != "NA")%>%
    column_to_rownames("Row.names")
d4
```

```
d4$Covid_PCR_result <- as.factor(d4$Covid_PCR_result)
d4$Covid_PCR_result <- relevel(d4$Covid_PCR_result, ref = "Negative")
```

```
set.seed(678194671)

optimal.cutpoint <- optimal.cutpoints(X = "Measurement" , 
                                      status = "Covid_PCR_result",
                                      tag.healthy = "Negative", 
                                      methods = "Youden",  
                                      data = d4, 
                                      pop.prev = NULL,
                                      control = control.cutpoints(),
                                      ci.fit = FALSE, 
                                      conf.level = 0.95, 
                                      trace = FALSE) 

summary(optimal.cutpoint)
```

```
## 
## Call:
## optimal.cutpoints.default(X = "Measurement", status = "Covid_PCR_result", 
##     tag.healthy = "Negative", methods = "Youden", data = d4, 
##     pop.prev = NULL, control = control.cutpoints(), ci.fit = FALSE, 
##     conf.level = 0.95, trace = FALSE)
## 
## Area under the ROC curve (AUC):  0.966 (0.934, 0.998) 
## 
## CRITERION: Youden
## Number of optimal cutoffs: 1
## 
##                      Estimate
## cutoff            118.8500000
## Se                  0.8769231
## Sp                  1.0000000
## PPV                 1.0000000
## NPV                 0.8333333
## DLR.Positive              Inf
## DLR.Negative        0.1230769
## FP                  0.0000000
## FN                  8.0000000
## Optimal criterion   0.8769231
```

## 4.3 Overal performance of CoVSense (Figure 2B and 2C)

Receiver operation characteristic (ROC) curve representing (A) sensitivity and specificity and (B) precision and recall

```
library(MLmetrics)
library(pROC)
```

```
d3 <- d_mean%>% 
  select(Measurement, Measurement2, 
         Covid_PCR_result, CT_2, CT_cat, 
         Panbio, Row.names, age,Variant, gender,    status,
         test_day_post_symptom_onset)%>% 
  filter(Measurement2 != "NA") %>%
  mutate(Measurement_cat = ifelse(Measurement2 < 118.85, 
                                  "Negative", "Positive"))%>%
  mutate(CT_cat2 = ifelse(CT_cat == ">37", 
                          "Negative", "Positive"))
d3
```

```
d3$Measurement2 <- as.numeric(d3$Measurement2)
d3$Covid_PCR_result <- as.factor(d3$Covid_PCR_result)
d3$Panbio <- as.factor(d3$Panbio)
d3$CT_cat2 <- as.factor(d3$CT_cat2)
d3$Measurement_cat <- as.factor(d3$Measurement_cat)
```

```
m <-  d3

mylogit1 <- glm(Covid_PCR_result ~ Measurement_cat,
                data = m, family = "binomial")
prob <- as.data.frame(predict(mylogit1,type=c("response")))


y <- m%>%
  select(Covid_PCR_result)%>%
  mutate(y = ifelse(Covid_PCR_result == "Positive", 1, 0))


pred <- as.data.frame(ifelse(mylogit1$fitted.values < 0.5, 0, 1))

data <- cbind(prob, y, pred)
data <- data %>%
  select(-Covid_PCR_result)%>% 
  rename(score = `predict(mylogit1, type = c("response"))`,true_class=y,
         pred_class= `ifelse(mylogit1$fitted.values < 0.5, 0, 1)`)


data$pred_class <- factor(data$pred_class, levels = c("1", "0"))
data$true_class <- factor(data$true_class, levels = c("1", "0"))


roc_dat <- data %>%
  roc_curve(true_class, score)


pr_dat <- data %>%
  pr_curve(true_class, score)

d_sens <- data%>%
    sens(true_class, pred_class)
  
print(paste0("Sensitivity = ", d_sens$.estimate))
```

```
## [1] "Sensitivity = 0.907692307692308"
```

```
d_spec <- data%>%
    spec(true_class, pred_class)
   
print(paste0("Specificity = ", d_spec$.estimate))
```

```
## [1] "Specificity = 1"
```

```
roc_dat <- roc_dat %>%
  rename(Specificity = specificity, Sensitivity = sensitivity)
roc_dat
```

```
pr_dat <- pr_dat %>%
  rename(Recall = recall, Precision = precision)
pr_dat
```

```
roc_dat %>%
  arrange(.threshold) %>% 
  ggplot() +
  geom_path(aes(1 - Specificity, Sensitivity), 
            position = position_dodge(width = 0.1)) + 
  geom_abline(intercept = 0, slope = 1, linetype = "dotted") + 
  coord_equal()+
 theme_bw()+
  theme(panel.grid.major = element_blank(), panel.grid.minor = element_blank())+
  guides(color=guide_legend(ncol=2))+
  theme(plot.title = element_text(size=10, hjust = 0.5, face = "bold"))+
  theme(axis.title = element_text(face = "bold", size = 12),
        legend.title = element_text(face = "bold"), 
        legend.text.align = 0,
        strip.text.x = element_text(face = "bold"))
```

```
pr_dat %>%
  arrange(.threshold) %>% 
  ggplot() +
  geom_path(aes(Recall, Precision), 
            position = position_dodge(width = 0.1)) + 
 theme_bw()+
  theme(panel.grid.major = element_blank(), panel.grid.minor = element_blank())+
  guides(color=guide_legend(ncol=2))+
  theme(plot.title = element_text(size=10, hjust = 0.5, face = "bold"))+
  theme(axis.title = element_text(face = "bold", size = 12),
        legend.title = element_text(face = "bold"), 
        legend.text.align = 0,
        strip.text.x = element_text(face = "bold"))
```

## 4.4 Figure 2D

CoVSense impedance measurements in relation to RT-PCR Ct values groups within the cohort

```
fig <- ggplot(d_mean, aes(CT_cat, Measurement2))+
  geom_jitter(aes(color=Covid_PCR_result, 
                  shape=Variant), 
              position=position_jitter(0.2)) +
  geom_boxplot(alpha = 0)+
  stat_summary(fun.data=data_summary, color="black", size=0.15)+
  theme_bw()+
  theme(panel.grid.major = element_blank(), panel.grid.minor = element_blank())+
  xlab("RT-PCR Ct value")+ ylab("Mean CoVSense signal (kΩ)")+
  theme(plot.title = element_text(size=10, hjust = 0.5, face = "bold"),
        axis.title = element_text(face = "bold", size = 12),
        legend.title = element_text(face = "bold"), 
        legend.text.align = 0)+
  scale_color_manual(name= "Clinical diagnosis\nbased on PCR",
                     values=c("goldenrod3",  "midnightblue"))
  
fig
```

```
mod <- aov(Measurement2 ~ CT_cat, data = d_mean)
summary(mod)
```

```
##              Df Sum Sq Mean Sq F value Pr(>F)    
## CT_cat        3 193622   64541   58.12 <2e-16 ***
## Residuals   101 112152    1110                   
## ---
## Signif. codes:  0 '***' 0.001 '**' 0.01 '*' 0.05 '.' 0.1 ' ' 1
```

```
TukeyHSD(mod)
```

```
##   Tukey multiple comparisons of means
##     95% family-wise confidence level
## 
## Fit: aov(formula = Measurement2 ~ CT_cat, data = d_mean)
## 
## $CT_cat
##                  diff        lwr         upr     p adj
## 25-30-<25   -27.14853  -55.11848   0.8214237 0.0604909
## 30-37-<25   -47.29536  -74.51083 -20.0798819 0.0000910
## >37-<25     -98.12640 -117.84588 -78.4069106 0.0000000
## 30-37-25-30 -20.14683  -53.67550  13.3818330 0.4004812
## >37-25-30   -70.97787  -98.76901 -43.1867322 0.0000000
## >37-30-37   -50.83104  -77.86271 -23.7993652 0.0000205
```

```
x <- d_mean %>%
  filter(Measurement2 < 121)%>%
  filter(Covid_PCR_result == "Positive")
unique(x$Patient_ID2)
```

```
## [1] "P4"   "P18"  "UK15" "UK17" "UK23" "UK24"
```

```
x <- d_mean %>%
  filter(Measurement2 < 121)%>%
  filter(Covid_PCR_result == "Negative")


x1 <- d_mean %>%
  filter(Measurement2 > 121)


z <- rbind(x, x1)
z
```

```
mod <- aov(Measurement2 ~ Covid_PCR_result, data = z)
summary(mod)
```

```
##                  Df Sum Sq Mean Sq F value Pr(>F)    
## Covid_PCR_result  1 195507  195507     199 <2e-16 ***
## Residuals        97  95291     982                   
## ---
## Signif. codes:  0 '***' 0.001 '**' 0.01 '*' 0.05 '.' 0.1 ' ' 1
```

## 4.5 Figure 2E

CoVSense impedance measurements in SARS-CoV-2 patients compared to patients with other viral upper respiratory infections

```
d_mean$Clinical_dx <- factor(d_mean$Clinical_dx, levels = c("COVID", "Influenza A", "Influenza B", "RSV", "None"))


fig <- ggplot(d_mean, aes(Clinical_dx, Measurement2))+
  geom_jitter(aes(color=Covid_PCR_result, 
                  shape = Variant), 
              position=position_jitter(0.2)) + 
  geom_boxplot(alpha = 0)+
  stat_summary(fun.data=data_summary, color="black", size=0.15)+
  theme_bw()+
  theme(panel.grid.major = element_blank(), panel.grid.minor = element_blank())+
   xlab("Patient sample type")+ ylab("Mean CoVSense signal (kΩ)")+
  theme(plot.title = element_text(size=10, hjust = 0.5, face = "bold"),
        axis.title = element_text(face = "bold",size = 12),
        legend.title = element_text(face = "bold"), 
        legend.text.align = 0)+
  scale_color_manual(name= "Clinical diagnosis\nbased on PCR",
                     values=c("goldenrod3",  "midnightblue"))
  
fig
```

```
mod <- aov(Measurement2 ~ Clinical_dx, data = d_mean)
summary(mod)
```

```
##              Df Sum Sq Mean Sq F value Pr(>F)    
## Clinical_dx   4 168822   42206   30.82 <2e-16 ***
## Residuals   100 136952    1370                   
## ---
## Signif. codes:  0 '***' 0.001 '**' 0.01 '*' 0.05 '.' 0.1 ' ' 1
```

```
TukeyHSD(mod)
```

```
##   Tukey multiple comparisons of means
##     95% family-wise confidence level
## 
## Fit: aov(formula = Measurement2 ~ Clinical_dx, data = d_mean)
## 
## $Clinical_dx
##                                diff        lwr        upr     p adj
## Influenza A-COVID       -79.8061026 -153.61531  -5.996898 0.0272399
## Influenza B-COVID       -68.4644359 -172.06452  35.135644 0.3589858
## RSV-COVID               -79.6994359 -153.50864  -5.890231 0.0275470
## None-COVID              -83.2264121 -104.78171 -61.671111 0.0000000
## Influenza B-Influenza A  11.3416667 -114.57709 137.260424 0.9991210
## RSV-Influenza A           0.1066667 -102.70557 102.918902 1.0000000
## None-Influenza A         -3.4203095  -78.16780  71.327186 0.9999401
## RSV-Influenza B         -11.2350000 -137.15376 114.683758 0.9991531
## None-Influenza B        -14.7619762 -119.03261  89.508661 0.9948670
## None-RSV                 -3.5269762  -78.27447  71.220519 0.9999323
```

## 4.6 Figure S3A and S3B

CoVSense impedance measurements in relation to RT-PCR Ct values groups within the cohort

```
fig <- ggplot(d_min, aes(CT_cat, Measurement2))+
  geom_jitter(aes(color=Covid_PCR_result), 
              position=position_jitter(0.2)) + 
  geom_boxplot(alpha = 0)+
  stat_summary(fun.data=data_summary, color="black", size=0.15)+
  theme_bw()+
  theme(panel.grid.major = element_blank(), panel.grid.minor = element_blank())+
 xlab("RT-PCR Ct value")+ ylab("Minimum CoVSense\nsignal (kΩ)")+
  theme(plot.title = element_text(size=10, hjust = 0.5, face = "bold"),
        axis.title = element_text(face = "bold", size = 12),
        legend.title = element_text(face = "bold"), 
        legend.text.align = 0)+
  scale_color_manual(name= "Clinical diagnosis\nbased on PCR",
                     values=c("goldenrod3",  "midnightblue"))
  
fig
```

CoVSense impedance measurements in SARS-CoV-2 patients compared to patients with other viral upper respiratory infections

```
d_min$Clinical_dx <- factor(d_min$Clinical_dx, levels = c("COVID", "Influenza A", "Influenza B", "RSV", "None"))


fig <- ggplot(d_min, aes(Clinical_dx, Measurement2))+
  geom_jitter(aes(color=Covid_PCR_result), 
              position=position_jitter(0.2)) + 
  geom_boxplot(alpha = 0)+
  stat_summary(fun.data=data_summary, color="black", size=0.15)+
  theme_bw()+
  theme(panel.grid.major = element_blank(), panel.grid.minor = element_blank())+
   xlab("Patient sample type")+ ylab("Minimum CoVSense\nsignal (kΩ)")+
  theme(plot.title = element_text(size=10, hjust = 0.5, face = "bold"),
        axis.title = element_text(face = "bold", size = 12),
        legend.title = element_text(face = "bold"), 
        legend.text.align = 0)+
  scale_color_manual(name= "Clinical diagnosis\nbased on PCR",
                     values=c("goldenrod3",  "midnightblue"))
  
fig
```

# 5 **Concordance between CoVSense and RT-PCR**

## 5.1 Data preparatoion: MinMax normalisation

```
x <- d_mean
x$CT_2 <- as.numeric(x$CT_2)

x1 <- x %>%
  mutate(Measurement3 = normalize(Measurement2), CT_3 = normalize2(CT_2))%>%
  mutate(mean=((Measurement3+CT_3)/2), delta = Measurement3-CT_3)
x1
```

## 5.2 Figure 2F

Correlation of RT-PCR Ct values with CoVSense impedance measurements. The measurements were MinMax scaled and assessed by Spearman rank correlation

```
fig <- ggplot(x1, aes(Measurement3, CT_3, color=Covid_PCR_result))+
  scale_color_manual(name= "Clinical diagnosis\nbased on PCR",
                     values=c("goldenrod3",  "midnightblue"))+
  geom_point()+
  theme_bw()+
  theme(panel.grid.major = element_blank(), panel.grid.minor = element_blank())+
  geom_smooth(method = "lm")+
  xlab("CoVSense signal (MinMax scaled)")+
  ylab("Ct value\n(Reverse MinMax scaled)")+
  theme(plot.title = element_text(size=10, hjust = 0.5, face = "bold"),
        axis.title = element_text(face = "bold", size = 12),
        legend.title = element_text(face = "bold"), 
        legend.text.align = 0)
fig
```

```
shapiro.test(x1$Measurement3)
```

```
## 
##  Shapiro-Wilk normality test
## 
## data:  x1$Measurement3
## W = 0.93319, p-value = 5.032e-05
```

```
shapiro.test(x1$CT_3)
```

```
## 
##  Shapiro-Wilk normality test
## 
## data:  x1$CT_3
## W = 0.84953, p-value = 6.167e-09
```

```
m <- x1%>% filter(Covid_PCR_result == "Positive")
res2 <-cor.test(m$Measurement3, m$CT_3,  method = "spearman")
res2
```

```
## 
##  Spearman's rank correlation rho
## 
## data:  m$Measurement3 and m$CT_3
## S = 20977, p-value = 3.183e-06
## alternative hypothesis: true rho is not equal to 0
## sample estimates:
##       rho 
## 0.5415765
```

## 5.3 Figure 2G

Lin’s concordance correlation coefficient (CCC), comparing the reliability and accuracy of CoVSense measurement compared to RT-PCR. The measurements were MinMax scaled

```
concord_test <- epi.ccc(x1$CT_3, 
                        x1$Measurement3, 
                        ci = "z-transform", 
                        conf.level = 0.95, 
                        rep.measure = FALSE, 
                        subjectid = Patient_ID)
concord_test$rho.c
```

```
concord_test$C.b
```

```
## [1] 0.9239005
```

```
fig <- ggplot(x1, aes(mean, delta, color=Covid_PCR_result))+
  scale_color_manual(name= "Clinical diagnosis\nbased on PCR",
                     values=c("goldenrod3",  "midnightblue"))+
  geom_point()+
  theme_bw()+
  theme(panel.grid.major = element_blank(), panel.grid.minor = element_blank())+
  geom_hline(data = concord_test$sblalt, 
             aes(yintercept = lower), linetype = 2) + 
  geom_hline(data = concord_test$sblalt, 
             aes(yintercept = upper), linetype = 2) + 
  geom_hline(data = concord_test$sblalt, 
             aes(yintercept = est), linetype = 1) +
  xlab("Mean (MinMax scaled)")+
  ylab("Absolute delta\n(MinMax scaled)")+
  theme(plot.title = element_text(size=10, hjust = 0.5, face = "bold"),
        axis.title = element_text(face = "bold", size = 12),
        legend.title = element_text(face = "bold"), 
        legend.text.align = 0)
fig
```

### 5.3.1 Overall concordance correlation coefficient

```
x2<- x1 %>%
  select(Measurement3,CT_3)
x2
```

```
epi.occc(x2, na.rm = FALSE, pairs = TRUE)
```

```
## 
## Overall CCC           0.7596
## Overall precision     0.8221
## Overall accuracy      0.9239
```

# 6 **Evaluating CoVSense in different settings**

## 6.1 Figure 3A

CoVSense measurements for wild-type and UK (B.1.1.7) variant compared to the controls.

```
d_mean$Variant <- factor(d_mean$Variant, levels = c("Control", "Wildtype", "Variant B.1.1.7"))
```

```
fig <- ggplot(d_mean, aes(Variant, Measurement2))+
  geom_jitter(aes(color=Covid_PCR_result), 
              position=position_jitter(0.2)) + 
  geom_boxplot(alpha = 0)+
  stat_summary(fun.data=data_summary, color="black", size=0.15)+
  theme_bw()+
  theme(panel.grid.major = element_blank(), panel.grid.minor = element_blank())+
  xlab("")+ 
  ylab("Mean CoVSense signal (kΩ)")+
  theme(plot.title = element_text(size=10, hjust = 0.5, face = "bold"),
        axis.title = element_text(face = "bold", size = 12),
        legend.title = element_text(face = "bold"), 
        legend.text.align = 0)+
  scale_color_manual(name= "Clinical diagnosis\nbased on PCR",
                     values=c("goldenrod3",  "midnightblue"))
  
fig
```

```
d_mean%>%
  group_by(Variant)%>%
  summarize_at("Measurement2", c(mean, sd, min, max))%>%
   rename(meam=fn1, sd=fn2, min=fn3, max=fn4)
```

```
mod <- aov(Measurement2 ~ Variant, data = d_mean)
summary(mod)
```

```
##              Df Sum Sq Mean Sq F value Pr(>F)    
## Variant       2 176245   88123   69.39 <2e-16 ***
## Residuals   102 129530    1270                   
## ---
## Signif. codes:  0 '***' 0.001 '**' 0.01 '*' 0.05 '.' 0.1 ' ' 1
```

```
TukeyHSD(mod)
```

```
##   Tukey multiple comparisons of means
##     95% family-wise confidence level
## 
## Fit: aov(formula = Measurement2 ~ Variant, data = d_mean)
## 
## $Variant
##                              diff        lwr       upr   p adj
## Wildtype-Control         70.77801 50.3074726  91.24854 0.00000
## Variant B.1.1.7-Control  92.56599 72.9487740 112.18321 0.00000
## Variant B.1.1.7-Wildtype 21.78798  0.7001095  42.87586 0.04118
```

## 6.2 Figure 3B

Field application of CoVSense in patients from the local community, emergency room (ER), inpatients, and nursing homes.

```
d2 <- d_mean %>%
  filter(status != "NA")


fig <- ggplot(d2, aes(Covid_PCR_result, Measurement2))+
  geom_jitter(aes(color=Covid_PCR_result), 
              position=position_jitter(0.2)) + 
  geom_boxplot(alpha = 0)+
  stat_summary(fun.data=data_summary, color="black", size=0.15)+
  theme_bw()+
  theme(panel.grid.major = element_blank(), panel.grid.minor = element_blank())+
  xlab("")+ 
   ylab("Mean CoVSense signal (kΩ)")+
  facet_wrap(~status)+
  theme(plot.title = element_text(size=10, hjust = 0.5, face = "bold"),
        axis.title = element_text(face = "bold", size = 12),
        legend.title = element_text(face = "bold"), 
        legend.text.align = 0,
        strip.text.x = element_text(face = "bold"))+
  scale_color_manual(name= "Clinical diagnosis\nbased on PCR",
                     values=c("goldenrod3",  "midnightblue"))
  
fig
```

```
for (i in unique(d2$status)){
  print(paste0(i))
  
  if (i == "Nursing home"){
    print(paste0("Missing category"))
  }else{
    m <- d2 %>%
    filter(status == i)
    mod <- aov(Measurement2 ~ Covid_PCR_result, data = m)
    print(summary(mod))
  }

}
```

```
## [1] "Inpatient"
##                  Df Sum Sq Mean Sq F value   Pr(>F)    
## Covid_PCR_result  1  24971   24971   121.9 6.88e-11 ***
## Residuals        24   4917     205                     
## ---
## Signif. codes:  0 '***' 0.001 '**' 0.01 '*' 0.05 '.' 0.1 ' ' 1
## [1] "Community"
##                  Df Sum Sq Mean Sq F value   Pr(>F)    
## Covid_PCR_result  1  50372   50372   29.44 2.49e-06 ***
## Residuals        43  73584    1711                     
## ---
## Signif. codes:  0 '***' 0.001 '**' 0.01 '*' 0.05 '.' 0.1 ' ' 1
## [1] "ER"
##                  Df Sum Sq Mean Sq F value   Pr(>F)    
## Covid_PCR_result  1  11850   11850   128.2 2.84e-05 ***
## Residuals         6    554      92                     
## ---
## Signif. codes:  0 '***' 0.001 '**' 0.01 '*' 0.05 '.' 0.1 ' ' 1
## [1] "Nursing home"
## [1] "Missing category"
```

## 6.3 Figure 3C

Gender-based CoVSense’s performance within the clinical cohort.

```
d2 <- d_mean %>%
  filter(gender != "NA")


fig <- ggplot(d2, aes(Covid_PCR_result, Measurement2))+
  geom_jitter(aes(color=Covid_PCR_result), 
              position=position_jitter(0.2)) + 
  geom_boxplot(alpha = 0)+
  stat_summary(fun.data=data_summary, color="black", size=0.15)+
  theme_bw()+
  theme(panel.grid.major = element_blank(), panel.grid.minor = element_blank())+
  xlab("")+ 
   ylab("Mean CoVSense signal (kΩ)")+
  facet_wrap(~gender)+
  theme(plot.title = element_text(size=10, hjust = 0.5, face = "bold"),
        axis.title = element_text(face = "bold", size = 12),
        legend.title = element_text(face = "bold"), 
        legend.text.align = 0,
        strip.text.x = element_text(face = "bold"))+
  scale_color_manual(name= "Clinical diagnosis\nbased on PCR",
                     values=c("goldenrod3",  "midnightblue"))
    
  
fig
```

```
for (i in unique(d2$gender)){
  print(paste0(i))
  m <- d2 %>%
    filter(gender == i)

mod <- aov(Measurement2 ~ Covid_PCR_result, data = m)
print(summary(mod))

}
```

```
## [1] "Female"
##                  Df Sum Sq Mean Sq F value   Pr(>F)    
## Covid_PCR_result  1 106054  106054   69.32 5.23e-11 ***
## Residuals        50  76497    1530                     
## ---
## Signif. codes:  0 '***' 0.001 '**' 0.01 '*' 0.05 '.' 0.1 ' ' 1
## [1] "Male"
##                  Df Sum Sq Mean Sq F value   Pr(>F)    
## Covid_PCR_result  1  64582   64582    57.7 7.96e-10 ***
## Residuals        49  54844    1119                     
## ---
## Signif. codes:  0 '***' 0.001 '**' 0.01 '*' 0.05 '.' 0.1 ' ' 1
```

## 6.4 Figure 3D

CoVSense measurements are not impacted by patient age assessed by Spearman rank correlation.

```
d2 <- d_mean %>%
  filter(age != "NA")%>%
  group_by(Covid_PCR_result)%>%
  summarise_at("age", c(min, max, mean, median))%>%
  rename(min=fn1, max=fn2, mean=fn3, median=fn4)
d2
```

```
d2 <- d_mean %>%
  filter(age != "NA")


fig <- ggplot(d2, aes(age, Measurement2))+
  geom_jitter(aes(color=Covid_PCR_result), 
              position=position_jitter(0.2)) + 
  geom_smooth(method = lm, se = FALSE, aes(color = Covid_PCR_result))+
  theme_bw()+
  theme(panel.grid.major = element_blank(), panel.grid.minor = element_blank())+
  xlab("Age (year)")+ 
  ylab("Mean CoVSense signal (kΩ)")+
  theme(plot.title = element_text(size=10, hjust = 0.5, face = "bold"),
        axis.title = element_text(face = "bold", size = 12),
        legend.title = element_text(face = "bold"), 
        legend.text.align = 0,
        strip.text.x = element_text(face = "bold"))+
  scale_color_manual(name= "Clinical diagnosis\nbased on PCR",
                     values=c("goldenrod3",  "midnightblue"))
  
fig
```

```
d2 <- d_mean %>%
  filter(age != "NA")

for (i in c(unique(d2$Covid_PCR_result))){
  print(paste0(i))
  d <- d2 %>% filter (Covid_PCR_result == i)

  
  res2 <-cor.test(d$Measurement2, d$age,  method = "spearman")
  print(res2)
}
```

```
## [1] "Positive"
## 
##  Spearman's rank correlation rho
## 
## data:  d$Measurement2 and d$age
## S = 49395, p-value = 0.5294
## alternative hypothesis: true rho is not equal to 0
## sample estimates:
##         rho 
## -0.07942807 
## 
## [1] "Negative"
## 
##  Spearman's rank correlation rho
## 
## data:  d$Measurement2 and d$age
## S = 12064, p-value = 0.4178
## alternative hypothesis: true rho is not equal to 0
## sample estimates:
##        rho 
## -0.1317382
```

## 6.5 Figure 3E

CoVSense measurements are not impacted by the time since symptom onset assessed by Spearman rank correlation.

```
d2 <- d_mean %>%
  filter(test_day_post_symptom_onset != "NA")%>%
  group_by(Covid_PCR_result)%>%
  summarise_at("test_day_post_symptom_onset", c(min, max, mean, median))%>%
  rename(min=fn1, max=fn2, mean=fn3, median=fn4)
d2
```

```
d2 <- d_mean %>%
  filter(test_day_post_symptom_onset != "NA")


fig <- ggplot(d2, aes(test_day_post_symptom_onset, Measurement2))+
  geom_jitter(aes(color=Covid_PCR_result), 
              position=position_jitter(0.2)) + 
  geom_smooth(method = lm, se = FALSE, 
              aes(color = Covid_PCR_result))+
  theme_bw()+
  theme(panel.grid.major = element_blank(), panel.grid.minor = element_blank())+
  xlab("Days since symptom onset")+ 
  ylab("Mean CoVSense signal (kΩ)")+
  theme(plot.title = element_text(size=10, hjust = 0.5, face = "bold"),
        axis.title = element_text(face = "bold", size = 12),
        legend.title = element_text(face = "bold"), 
        legend.text.align = 0,
        strip.text.x = element_text(face = "bold"))+
  scale_color_manual(name= "Clinical diagnosis\nbased on PCR", 
                     values=c( "#542788"))+
  xlim(0,15)
  
   
fig
```

```
d2 <- d_mean %>%
  filter(test_day_post_symptom_onset != "NA")

d <- d2 %>% filter (Covid_PCR_result == "Positive")

res2 <-cor.test(d$Measurement2, d$age,  method = "spearman")
res2
```

```
## 
##  Spearman's rank correlation rho
## 
## data:  d$Measurement2 and d$age
## S = 42609, p-value = 0.5729
## alternative hypothesis: true rho is not equal to 0
## sample estimates:
##         rho 
## -0.07298609
```

# 7 **CoVSense performance assessment**

## 7.1 Figure S5

```
d3 <- d_mean%>% 
  select(Measurement, Measurement2, 
         Covid_PCR_result, CT_2, 
         CT_cat, Panbio, 
         Row.names, age,Variant, 
         gender, status, test_day_post_symptom_onset)%>% 
  filter(Measurement2 != "NA") %>%
  mutate(Measurement_cat = ifelse(Measurement2 < 118.85, "Negative", "Positive"))%>%
  mutate(CT_cat2 = ifelse(CT_cat == ">37", "Negative", "Positive"))%>%
  column_to_rownames("Row.names")


d3$Measurement2 <- as.numeric(d3$Measurement2)
d3$Covid_PCR_result <- as.factor(d3$Covid_PCR_result)
```

```
m <- d3 %>%
  group_by(CT_cat2, Measurement_cat)%>%
  summarise(n = n()) %>%
  mutate(freq = n*100 / sum(n))%>%
  mutate(Performance = recode(CT_cat2,
            "Negative" = ifelse(Measurement_cat == "Negative", "TN", "FP"),
            "Positive" = ifelse(Measurement_cat == "Positive", "TP", "FN")))%>%
  mutate(Performance2 = recode(CT_cat2,
            "Negative" = ifelse(Measurement_cat == "Negative", "True", "False"),
            "Positive" = ifelse(Measurement_cat == "Positive", "True", "False")))%>%
  ungroup()%>%
  add_row(CT_cat2 = "Negative", 
          Measurement_cat = "Positive", 
          n= 0, 
          freq = 0, 
          Performance = "FP", 
          Performance2 = "False", .before = 2)
m
```

```
fig <- ggplot(m, aes(CT_cat2, Measurement_cat, fill= Performance2)) + 
  geom_tile()+
  geom_text(aes(label=paste0(Performance,"\n N=", n, 
                             " (", round(freq, digits = 1), "%)")))+
  theme_minimal()+
  theme(plot.title = element_text(size=10, hjust = 0.5, face = "bold"),
        axis.title = element_blank(),
        legend.title = element_text(face = "bold"), 
        legend.text.align = 0,
        axis.text = element_blank(),
        panel.border = element_blank(), 
        panel.grid.major = element_blank(),
        panel.grid.minor = element_blank())+
  scale_fill_manual(name= "Biosensor\nperformance",
                    values=c("coral1","aquamarine3"))
fig
```

```
m <- d3 %>%
  filter(gender != "NA")%>%
  group_by(gender, CT_cat2, Measurement_cat)%>%
  summarise(n = n()) %>%
  mutate(freq = n*100 / sum(n))%>%
  mutate(Performance = recode(CT_cat2,
                             "Negative" = ifelse(Measurement_cat == "Negative", 
                                                 "TN", "FP"),
                             "Positive" = ifelse(Measurement_cat == "Positive", 
                                                 "TP", "FN")))%>%
  mutate(Performance2 = recode(CT_cat2,
                             "Negative" = ifelse(Measurement_cat == "Negative", 
                                                 "True", "False"),
                             "Positive" = ifelse(Measurement_cat == "Positive", 
                                                 "True", "False")))%>%
  ungroup()%>%
  add_row(gender = "Female", 
          CT_cat2 = "Negative", 
          Measurement_cat = "Positive", 
          n= 0, 
          freq = 0, 
          Performance = "FP", 
          Performance2 = "False", .before = 2)%>%
  add_row(gender = "Male", 
          CT_cat2 = "Negative", 
          Measurement_cat = "Positive", 
          n= 0, 
          freq = 0, 
          Performance = "FP", 
          Performance2 = "False", .before = 6)

m
```

```
fig <- ggplot(m, aes(CT_cat2, Measurement_cat, fill= Performance2)) + 
  geom_tile()+
  geom_text(aes(label=paste0(Performance,"\n N=", n, 
                             " (", round(freq, digits = 1), "%)")))+
  theme_minimal()+
  facet_wrap(~gender)+
  theme(plot.title = element_text(size=10, hjust = 0.5, face = "bold"),
        strip.text.x = element_text(face = "bold", size = 12),
        axis.title = element_blank(),
        legend.title = element_text(face = "bold"), 
        legend.text.align = 0,
        axis.text = element_blank(),
        panel.border = element_blank(), 
        panel.grid.major = element_blank(),
        panel.grid.minor = element_blank())+
  scale_fill_manual(name= "Biosensor\nperformance",
                    values=c("coral1","aquamarine3"))
fig
```

```
m <- d3 %>%
  filter(Variant != "NA")%>%
  group_by(Variant, CT_cat2, Measurement_cat)%>%
  summarise(n = n()) %>%
  mutate(freq = n*100 / sum(n))%>%
  mutate(Performance = recode(CT_cat2,
                             "Negative" = ifelse(Measurement_cat == "Negative", 
                                                 "TN", "FP"),
                             "Positive" = ifelse(Measurement_cat == "Positive", 
                                                 "TP", "FN")))%>%
  mutate(Performance2 = recode(CT_cat2,
                             "Negative" = ifelse(Measurement_cat == "Negative", 
                                                 "True", "False"),
                             "Positive" = ifelse(Measurement_cat == "Positive", 
                                                 "True", "False")))%>%
  ungroup()%>%
  add_row(Variant = "Wildtype", 
          CT_cat2 = "Negative", 
          Measurement_cat = "Negative", 
          n= 0, 
          freq = 0, 
          Performance = "TN", 
          Performance2 = "True", .before = 2)%>%
   add_row(Variant = "Wildtype", 
            CT_cat2 = "Negative", 
            Measurement_cat = "Positive",
            n= 0, 
            freq = 0, 
            Performance = "FP", 
            Performance2 = "False", .before = 3)%>%
  add_row(Variant = "Variant B.1.1.7", 
          CT_cat2 = "Negative", 
          Measurement_cat = "Negative",
          n= 0, 
          freq = 0, 
          Performance = "TN", 
          Performance2 = "True", .before = 6)%>%
    add_row(Variant = "Variant B.1.1.7", 
            CT_cat2 = "Negative", 
            Measurement_cat = "Positive",
            n= 0, 
            freq = 0, 
            Performance = "FP", 
            Performance2 = "False", .before = 7)
    
m
```

```
m$Variant <- factor(m$Variant, levels = c("Wildtype", "Variant B.1.1.7"))
```

```
m1 <- m%>% filter(Variant != "Control")


fig <- ggplot(m1, aes(CT_cat2, Measurement_cat, fill= Performance2)) + 
  geom_tile()+
  geom_text(aes(label=paste0(Performance,"\n N=", n,
                             " (", round(freq, digits = 1), "%)")))+
  theme_minimal()+
  facet_wrap(~Variant)+
  theme(plot.title = element_text(size=10, hjust = 0.5, face = "bold"),
        strip.text.x = element_text(face = "bold", size = 12),
        axis.title = element_blank(),
        legend.title = element_text(face = "bold"), 
        legend.text.align = 0,
        axis.text = element_blank(),
        panel.border = element_blank(), 
        panel.grid.major = element_blank(),
        panel.grid.minor = element_blank())+
  scale_fill_manual(name= "Biosensor\nperformance",
                    values=c("coral1","aquamarine3"))
fig
```

```
library(MLmetrics)
library(pROC)
```

## 7.2 Figure 4A & B

Receiver operation characteristic (ROC) curve representing (A) sensitivity and specificity and (B) precision and recall stratified by patient gender and viral variants

```
d3 <- d_mean%>% 
  select(Measurement, Measurement2, 
         Covid_PCR_result, CT_2, CT_cat, 
         Panbio, Row.names, age,Variant, gender,    status,
         test_day_post_symptom_onset)%>% 
  filter(Measurement2 != "NA") %>%
  mutate(Measurement_cat = ifelse(Measurement2 < 118.85, 
                                  "Negative", "Positive"))%>%
  mutate(CT_cat2 = ifelse(CT_cat == ">37", 
                          "Negative", "Positive"))
d3
```

```
d3$Measurement2 <- as.numeric(d3$Measurement2)
d3$Covid_PCR_result <- as.factor(d3$Covid_PCR_result)
d3$Panbio <- as.factor(d3$Panbio)
d3$CT_cat2 <- as.factor(d3$CT_cat2)
d3$Measurement_cat <- as.factor(d3$Measurement_cat)
```

```
m <-  d3%>% filter(Variant != "Wildtype")

mylogit1 <- glm(Covid_PCR_result ~ Measurement_cat, 
                data = m, family = "binomial")
prob <- as.data.frame(predict(mylogit1,type=c("response")))


y <- m%>%
  select(Covid_PCR_result)%>%
  mutate(y = ifelse(Covid_PCR_result == "Positive", 1, 0))


pred <- as.data.frame(ifelse(mylogit1$fitted.values < 0.5, 0, 1))

data <- cbind(prob, y, pred)
data <- data %>%
  select(-Covid_PCR_result)%>% 
  rename(score = `predict(mylogit1, type = c("response"))`,true_class=y,
         pred_class= `ifelse(mylogit1$fitted.values < 0.5, 0, 1)`)

data$pred_class <- factor(data$pred_class, levels = c("1", "0"))
data$true_class <- factor(data$true_class, levels = c("1", "0"))


roc_dat1 <- data %>%
  roc_curve(true_class, score)%>%
  add_column(Factor = "Variant B.1.1.7")


pr_dat1 <- data %>%
  pr_curve(true_class, score)%>%
  add_column(Factor = "Variant B.1.1.7")
```

```
m <-  d3%>% filter(Variant != "Variant B.1.1.7")

mylogit1 <- glm(Covid_PCR_result ~ Measurement_cat,
                data = m, family = "binomial")
prob <- as.data.frame(predict(mylogit1,type=c("response")))


y <- m%>%
  select(Covid_PCR_result)%>%
  mutate(y = ifelse(Covid_PCR_result == "Positive", 1, 0))


pred <- as.data.frame(ifelse(mylogit1$fitted.values < 0.5, 0, 1))

data <- cbind(prob, y, pred)
data <- data %>%
  select(-Covid_PCR_result)%>% 
  rename(score = `predict(mylogit1, type = c("response"))`,true_class=y,
         pred_class= `ifelse(mylogit1$fitted.values < 0.5, 0, 1)`)


data$pred_class <- factor(data$pred_class, levels = c("1", "0"))
data$true_class <- factor(data$true_class, levels = c("1", "0"))


roc_dat2 <- data %>%
  roc_curve(true_class, score)%>%
  add_column(Factor = "Wildtype")


pr_dat2 <- data %>%
  pr_curve(true_class, score)%>%
  add_column(Factor = "Wildtype")
```

```
m <-  d3%>% filter(gender == "Male")

mylogit1 <- glm(Covid_PCR_result ~ Measurement_cat, 
                data = m, family = "binomial")
prob <- as.data.frame(predict(mylogit1,type=c("response")))


y <- m%>%
  select(Covid_PCR_result)%>%
  mutate(y = ifelse(Covid_PCR_result == "Positive", 1, 0))


pred <- as.data.frame(ifelse(mylogit1$fitted.values < 0.5, 0, 1))

data <- cbind(prob, y, pred)
data <- data %>%
  select(-Covid_PCR_result)%>% 
  rename(score = `predict(mylogit1, type = c("response"))`,true_class=y,
         pred_class= `ifelse(mylogit1$fitted.values < 0.5, 0, 1)`)


data$pred_class <- factor(data$pred_class, levels = c("1", "0"))
data$true_class <- factor(data$true_class, levels = c("1", "0"))


roc_dat3 <- data %>%
  roc_curve(true_class, score)%>%
  add_column(Factor = "Male")


pr_dat3 <- data %>%
  pr_curve(true_class, score)%>%
  add_column(Factor = "Male")
```

```
m <-  d3%>% filter(gender == "Female")

mylogit1 <- glm(Covid_PCR_result ~ Measurement_cat,
                data = m, family = "binomial")
prob <- as.data.frame(predict(mylogit1,type=c("response")))


y <- m%>%
  select(Covid_PCR_result)%>%
  mutate(y = ifelse(Covid_PCR_result == "Positive", 1, 0))


pred <- as.data.frame(ifelse(mylogit1$fitted.values < 0.5, 0, 1))

data <- cbind(prob, y, pred)
data <- data %>%
  select(-Covid_PCR_result)%>% 
  rename(score = `predict(mylogit1, type = c("response"))`,true_class=y,
         pred_class= `ifelse(mylogit1$fitted.values < 0.5, 0, 1)`)


data$pred_class <- factor(data$pred_class, levels = c("1", "0"))
data$true_class <- factor(data$true_class, levels = c("1", "0"))


roc_dat4 <- data %>%
  roc_curve(true_class, score)%>%
  add_column(Factor = "Female")


pr_dat4 <- data %>%
  pr_curve(true_class, score)%>%
  add_column(Factor = "Female")
```

```
roc_dat <- rbind(roc_dat1, roc_dat2, roc_dat3, roc_dat4)
pr_dat <- rbind(pr_dat1, pr_dat2, pr_dat3, pr_dat4)
```

```
roc_dat <- roc_dat %>%
  rename(Specificity = specificity, Sensitivity = sensitivity)%>%
  mutate(Category = 
           ifelse(Factor %in% c("Wildtype", "Variant B.1.1.7"),
                           "Variant", "Gender"))
roc_dat
```

```
pr_dat <- pr_dat %>%
  rename(Recall = recall, Precision = precision)%>%
  mutate(Category = 
           ifelse(Factor %in% c("Wildtype", "Variant B.1.1.7"),
                           "Variant", "Gender"))
pr_dat
```

```
roc_dat$Factor <- factor(roc_dat$Factor, levels = c("Female", "Male", "Wildtype", "Variant B.1.1.7"))
```

```
roc_dat %>%
  arrange(.threshold) %>% 
  ggplot() +
  geom_path(aes(1 - Specificity, Sensitivity, color=Factor), 
            position = position_dodge(width = 0.1)) + 
  geom_abline(intercept = 0, slope = 1, linetype = "dotted") + 
  coord_equal()+
   scale_color_manual(
     values=c("darkblue", "darkgoldenrod1", "brown3",  "lavenderblush4"))+
 theme_bw()+
  theme(panel.grid.major = element_blank(), panel.grid.minor = element_blank())+
  guides(color=guide_legend(ncol=2))+
  facet_wrap(~Category)+
  theme(plot.title = element_text(size=10, hjust = 0.5, face = "bold"))+
  theme(axis.title = element_text(face = "bold", size = 12),
        legend.title = element_text(face = "bold"), 
        legend.text.align = 0,
        strip.text.x = element_text(face = "bold"))
```

```
pr_dat$Factor <- factor(pr_dat$Factor, levels = c("Female", "Male", "Wildtype", "Variant B.1.1.7"))
```

```
pr_dat %>%
  arrange(.threshold) %>% 
  ggplot() +
  geom_path(aes(Recall, Precision, color=Factor), 
            position = position_dodge(width = 0.1)) + 
  coord_equal()+
   scale_color_manual(
     values=c("darkblue", "darkgoldenrod1", "brown3",  "lavenderblush4"))+
 theme_bw()+
  theme(panel.grid.major = element_blank(), panel.grid.minor = element_blank())+
  guides(color=guide_legend(ncol=2))+
  facet_wrap(~Category)+
  theme(plot.title = element_text(size=10, hjust = 0.5, face = "bold"))+
  theme(axis.title = element_text(face = "bold", size = 12),
        legend.title = element_text(face = "bold"), 
        legend.text.align = 0,
        strip.text.x = element_text(face = "bold"))
```

```
for (i in c("Wildtype", "Variant B.1.1.7")){
  print(paste0("is not ", i))

  m <-  d3%>% filter(Variant != i)

  mylogit1 <- glm(Covid_PCR_result ~ Measurement_cat , 
                  data = m, family = "binomial")
  prob = predict(mylogit1,type=c("response"))


  y <- ifelse(m$Covid_PCR_result == "Positive", 1, 0)

  pred <- ifelse(mylogit1$fitted.values < 0.5, 0, 1)


  print(paste0("Accuracy = ", round(Accuracy(pred, y), digits = 2)))
  print(paste0("AUC = ", round(AUC(mylogit1$fitted.values, y), digits = 2)))


  ConfusionMatrix(y_pred = pred, y_true = y)

  g_ech <- roc(Covid_PCR_result ~ prob, data = m)
  print(paste0("AUC = ", round(g_ech$auc, digits = 2)))  ###AUC
  print(paste0("AUC CI = ", round(ci(g_ech), digits = 2))) ###Confidence Interval

  print(paste0("F1 for positive state = ", 
               round(F1_Score(y_pred = pred, 
                              y_true = y, 
                              positive = "1"), 
                     digits = 2)))
  print(paste0("F1 for negative state = ", 
               round(F1_Score(y_pred = pred, 
                              y_true = y, 
                              positive = "0"), 
                     digits = 2)))


  print(paste0("Recall for positive state = ", 
               round(Recall(y_pred = pred, 
                            y_true = y, 
                            positive = "1"), 
                     digits = 2))) 
  print(paste0("Recall for negative state = ", 
               round(Recall(y_pred = pred, 
                            y_true = y, 
                            positive = "0"), 
                     digits = 2)))
}
```

```
## [1] "is not Wildtype"
## [1] "Accuracy = 0.95"
## [1] "AUC = 0.94"
## [1] "AUC = 0.94"
## [1] "AUC CI = 0.89" "AUC CI = 0.94" "AUC CI = 1"   
## [1] "F1 for positive state = 0.94"
## [1] "F1 for negative state = 0.95"
## [1] "Recall for positive state = 0.89"
## [1] "Recall for negative state = 1"
## [1] "is not Variant B.1.1.7"
## [1] "Accuracy = 0.97"
## [1] "AUC = 0.97"
## [1] "AUC = 0.97"
## [1] "AUC CI = 0.92" "AUC CI = 0.97" "AUC CI = 1"   
## [1] "F1 for positive state = 0.97"
## [1] "F1 for negative state = 0.98"
## [1] "Recall for positive state = 0.93"
## [1] "Recall for negative state = 1"
```

```
for (i in c("Male", "Female")){
  print(paste0("is ", i))

  m <-  d3%>% filter(gender == i)

  mylogit1 <- glm(Covid_PCR_result ~ Measurement_cat , 
                  data = m, family = "binomial")
  prob = predict(mylogit1,type=c("response"))


  y <- ifelse(m$Covid_PCR_result == "Positive", 1, 0)

  pred <- ifelse(mylogit1$fitted.values < 0.5, 0, 1)


  print(paste0("Accuracy = ", round(Accuracy(pred, y), digits = 2)))
  print(paste0("AUC = ", round(AUC(mylogit1$fitted.values, y), digits = 2)))


  ConfusionMatrix(y_pred = pred, y_true = y)
  
 

  g_ech <- roc(Covid_PCR_result ~ prob, data = m)
  print(paste0("AUC = ", round(g_ech$auc, digits = 2)))  ###AUC
  print(paste0("AUC CI = ", round(ci(g_ech), digits = 2))) ###Confidence Interval


  print(paste0("F1 for positive state = ", 
               round(F1_Score(y_pred = pred, 
                              y_true = y, 
                              positive = "1"), 
                     digits = 2)))
  print(paste0("F1 for negative state = ", 
               round(F1_Score(y_pred = pred, 
                              y_true = y, 
                              positive = "0"), 
                     digits = 2)))


  print(paste0("Recall for positive state = ", 
               round(Recall(y_pred = pred, 
                            y_true = y, 
                            positive = "1"), 
                     digits = 2))) 
  print(paste0("Recall for negative state = ", 
               round(Recall(y_pred = pred, 
                            y_true = y, 
                            positive = "0"), 
                     digits = 2)))
}
```

```
## [1] "is Male"
## [1] "Accuracy = 0.92"
## [1] "AUC = 0.94"
## [1] "AUC = 0.94"
## [1] "AUC CI = 0.88" "AUC CI = 0.94" "AUC CI = 1"   
## [1] "F1 for positive state = 0.93"
## [1] "F1 for negative state = 0.9"
## [1] "Recall for positive state = 0.88"
## [1] "Recall for negative state = 1"
## [1] "is Female"
## [1] "Accuracy = 0.96"
## [1] "AUC = 0.97"
## [1] "AUC = 0.97"
## [1] "AUC CI = 0.93" "AUC CI = 0.97" "AUC CI = 1"   
## [1] "F1 for positive state = 0.97"
## [1] "F1 for negative state = 0.95"
## [1] "Recall for positive state = 0.94"
## [1] "Recall for negative state = 1"
```

```
df <- data.frame (Factor  = c("Male", "Female", 
                              "Wildtype", "Variant B.1.1.7"),
                  Accuracy = c(0.92, 0.96, 0.97, 0.95),
                  AUC = c(0.94, 0.97,0.97, 0.94 ),
                  F1_for_positive = c(0.93, 0.97, 0.97 , 0.94),
                  F1_for_negative = c(0.90, 0.95, 0.98, 0.95))%>%
  gather(Accuracy:F1_for_negative, key= Parameter, value = Value)%>%
  mutate(Parameter = recode(Parameter,
                           "F1_for_positive" = "F1 for positive",
                           "F1_for_negative" = "F1 for negative"))
df
```

```
df$Factor <- factor(df$Factor, levels = c("Variant B.1.1.7", "Wildtype",  "Male", "Female"))
```

```
fig <- ggplot(df, aes(Factor, Value, fill = Parameter))+
  geom_bar(stat = "identity", position = position_dodge(), color= "black")+
  coord_flip()+
  theme_bw()+
  theme(panel.grid.major = element_blank(), panel.grid.minor = element_blank())+
  scale_fill_brewer()+
  xlab("")+
  theme(plot.title = element_text(size=10, hjust = 0.5, face = "bold"))+
  theme(axis.title = element_text(face = "bold"),
        legend.title = element_text(face = "bold"), 
        legend.text.align = 0,
        strip.text.x = element_text(face = "bold"),
        axis.text.y = element_text(face = "bold"))
fig
```

## 7.3 Figure 4C

Conditional probabilities showing the diagnostic capability of the CoVSense in the clinical cohort according to the pre-test probabilities of infection stratified by patient gender and viral variant

```
#Leaf plot v 0.9
#Script by Fernando Zampieri
#fzampieri@hcor.com.br
#Use and distribute freely!

leafplot<-function(sens,spec){
  db <- tibble(prob.pre = seq(0.001, 0.999, by=0.001),
             odds.pre = prob.pre / (1 - prob.pre),
             plr = sens / (1 - spec),
             nlr = (1 - sens) / spec,
             odds.pos.p = odds.pre * plr,
             prob.pos.p =odds.pos.p / (1 + odds.pos.p),
             odds.pos.n =odds.pre * nlr,
             prob.pos.n =odds.pos.n / (1 + odds.pos.n))
  return(db)
}
```

```
m <-  d3

mylogit1 <- glm(Covid_PCR_result ~ Measurement_cat,
                data = m, family = "binomial")
prob <- as.data.frame(predict(mylogit1,type=c("response")))


y <- m%>%
  select(Covid_PCR_result)%>%
  mutate(y = ifelse(Covid_PCR_result == "Positive", 1, 0))


pred <- as.data.frame(ifelse(mylogit1$fitted.values < 0.5, 0, 1))

data <- cbind(prob, y, pred)
data <- data %>%
  select(-Covid_PCR_result)%>% 
  rename(score = `predict(mylogit1, type = c("response"))`,true_class=y,
         pred_class= `ifelse(mylogit1$fitted.values < 0.5, 0, 1)`)


data$pred_class <- factor(data$pred_class, levels = c("1", "0"))
data$true_class <- factor(data$true_class, levels = c("1", "0"))


d_sens <- data%>%
    sens(true_class, pred_class)
  
print(paste0("Sensitivity = ", d_sens$.estimate))
```

```
## [1] "Sensitivity = 0.907692307692308"
```

```
d_spec <- data%>%
    spec(true_class, pred_class)
   
print(paste0("Specificity = ", d_spec$.estimate))
```

```
## [1] "Specificity = 1"
```

```
for (i in c("Wildtype", "Variant B.1.1.7")){
  print(paste0("is not ", i))

  m <-  d3%>% filter(Variant != i)

mylogit1 <- glm(Covid_PCR_result ~ Measurement_cat,
                data = m, family = "binomial")
prob <- as.data.frame(predict(mylogit1,type=c("response")))


y <- m%>%
  select(Covid_PCR_result)%>%
  mutate(y = ifelse(Covid_PCR_result == "Positive", 1, 0))


pred <- as.data.frame(ifelse(mylogit1$fitted.values < 0.5, 0, 1))

data <- cbind(prob, y, pred)
data <- data %>%
  select(-Covid_PCR_result)%>% 
  rename(score = `predict(mylogit1, type = c("response"))`,true_class=y,
         pred_class= `ifelse(mylogit1$fitted.values < 0.5, 0, 1)`)


data$pred_class <- factor(data$pred_class, levels = c("1", "0"))
data$true_class <- factor(data$true_class, levels = c("1", "0"))


d_sens <- data%>%
    sens(true_class, pred_class)
  
print(paste0("Sensitivity = ", d_sens$.estimate))

d_spec <- data%>%
    spec(true_class, pred_class)
   
print(paste0("Specificity = ", d_spec$.estimate))

}
```

```
## [1] "is not Wildtype"
## [1] "Sensitivity = 0.885714285714286"
## [1] "Specificity = 1"
## [1] "is not Variant B.1.1.7"
## [1] "Sensitivity = 0.933333333333333"
## [1] "Specificity = 1"
```

```
for (i in c("Male", "Female")){
  print(paste0("is ", i))

  m <-  d3%>% filter(gender == i)

mylogit1 <- glm(Covid_PCR_result ~ Measurement_cat,
                data = m, family = "binomial")
prob <- as.data.frame(predict(mylogit1,type=c("response")))


y <- m%>%
  select(Covid_PCR_result)%>%
  mutate(y = ifelse(Covid_PCR_result == "Positive", 1, 0))


pred <- as.data.frame(ifelse(mylogit1$fitted.values < 0.5, 0, 1))

data <- cbind(prob, y, pred)
data <- data %>%
  select(-Covid_PCR_result)%>% 
  rename(score = `predict(mylogit1, type = c("response"))`,true_class=y,
         pred_class= `ifelse(mylogit1$fitted.values < 0.5, 0, 1)`)


data$pred_class <- factor(data$pred_class, levels = c("1", "0"))
data$true_class <- factor(data$true_class, levels = c("1", "0"))


d_sens <- data%>%
    sens(true_class, pred_class)
  
print(paste0("Sensitivity = ", d_sens$.estimate))

d_spec <- data%>%
    spec(true_class, pred_class)
   
print(paste0("Specificity = ", d_spec$.estimate))

}
```

```
## [1] "is Male"
## [1] "Sensitivity = 0.875"
## [1] "Specificity = 1"
## [1] "is Female"
## [1] "Sensitivity = 0.9375"
## [1] "Specificity = 1"
```

### 7.3.1 Male

```
d <- leafplot(0.875,0.99)


  
fig <-  d %>%
        select(prob.pre,prob.pos.p,prob.pos.n)%>%
        ggplot(aes(x = prob.pre, y = prob.pos.p))+
        geom_line()+
        geom_line(inherit.aes=FALSE, aes(x = prob.pre, y = prob.pos.n))+
        geom_abline(slope=1, intercept=0, color="brown", size = 1.2)+
        geom_ribbon(aes(x = prob.pre, ymax = prob.pos.p, ymin = prob.pos.n),
                    alpha = 0.5, fill = "darkgreen")+
        coord_cartesian(xlim = c(0,1), expand = TRUE, ylim = c(0,1))+
        scale_y_continuous(labels=scales::percent)+
        scale_x_continuous(labels=scales::percent)+
        labs(x = "Pre-test probability", 
             y = "Post-test probability", 
             title = "Male")+
        annotate("text", 
                 x = 0.12, 
                 y = 0.999, 
                 label = "Positive Test", 
                 size=3)+
        annotate("text", 
                 x = 0.923, 
                 y = 0.02, 
                 label = "Negative Test", 
                 size=3)+
        theme_bw()+
        theme(panel.grid.major = element_blank(), panel.grid.minor = element_blank())+
        theme(axis.title = element_text(face = "bold", size = 12), 
              plot.title = element_text(size=10, hjust = 0.5, face = "bold"))
  
fig
```

```
d %>% filter(prob.pre == 0.75) %>% select(prob.pos.p, prob.pos.n)
```

### 7.3.2 Female

```
d %>% filter(prob.pre == 0.75) %>% select(prob.pos.p, prob.pos.n)
```

### 7.3.3 Wildtype virus

### 7.3.4 Variant B.1.1.7

## 7.4 Figure 4D-I

The distribution of CoVSense SARS-CoV-2 infection diagnosis results based on (D) gender, (E) virus variant, (F) symptoms, (G) age, (H) days post symptom onset, and (I) Ct-value

### 7.4.1 Gender

```
d4 <- d_mean %>%
  mutate(Measurement_cat = ifelse(Measurement2 < 118.85, "Negative", "Positive"))%>%
  mutate(biosensor_pcr_concordance = recode(Covid_PCR_result,
                             "Negative" = ifelse(Measurement_cat == "Negative", "TN", "FP"),
                             "Positive" = ifelse(Measurement_cat == "Positive", "TP", "FN")))%>%
  select(Measurement2, sampling, CT_2, CT_cat, InfluenzaA, InfluenzaB, RSV, Variant, age, gender, status, Sympotamtic_based.on.Lab.database, test_day_post_symptom_onset, biosensor_pcr_concordance)
d4
```

```
d5 <- d4 %>%
  group_by(biosensor_pcr_concordance, gender)%>%
  filter(gender != "NA")%>%
  summarise(n = n()) %>%
  mutate(freq = n*100 / sum(n))
d5
```

```
fig <- ggplot(d5, aes(biosensor_pcr_concordance, freq, fill=gender, label=n))+
  geom_bar(stat = "identity", colour="black", width = 0.5)+
  labs(y="Proportion", x="CoVSense", fill = "Gender")+
  theme_bw()+
  theme(panel.grid.major = element_blank(), panel.grid.minor = element_blank())+
  guides(fill = guide_legend(reverse=F))+
  ggtitle("")+
  scale_y_continuous(expand = c(0, 0))+
  theme(plot.title = element_text(size=9, hjust = 0.5, face = "bold.italic"))+
  scale_fill_manual(values=c("cornflowerblue","honeydew3"))+
  theme(axis.title = element_text(face = "bold", size = 12),
        legend.title = element_text(face = "bold"), 
        legend.text.align = 0, 
        strip.text.x = element_text(face = "bold"))+
  geom_text(size = 3, position = position_stack(vjust = 0.5))
 
  
fig
```

### 7.4.2 Virus variant

```
d5 <- d4 %>%
  group_by(biosensor_pcr_concordance, Variant)%>%
  filter(Variant != "Control")%>%
  summarise(n = n()) %>%
  mutate(freq = n*100 / sum(n))
d5
```

```
d5$Variant <- factor(d5$Variant, levels = c("Wildtype", "Variant B.1.1.7"))


fig <- ggplot(d5, aes(biosensor_pcr_concordance, freq, fill=Variant, label=n))+
  geom_bar(stat = "identity", colour="black", width = 0.5)+
  labs(y="Proportion", x="CoVSense", fill = "Variant")+
  theme_bw()+
  theme(panel.grid.major = element_blank(), panel.grid.minor = element_blank())+
  guides(fill = guide_legend(reverse=F))+
  ggtitle("")+
  scale_y_continuous(expand = c(0, 0))+
  theme(plot.title = element_text(size=9, hjust = 0.5, face = "bold.italic"))+
  scale_fill_manual(values=c("cornflowerblue","honeydew3"))+
  theme(axis.title = element_text(face = "bold", size = 12),
        legend.title = element_text(face = "bold"), 
        legend.text.align = 0, 
        strip.text.x = element_text(face = "bold"))+
  geom_text(size = 3, position = position_stack(vjust = 0.5))
 
  
fig
```

### 7.4.3 Symptoms

```
d5 <- d4 %>%
  group_by(biosensor_pcr_concordance, Sympotamtic_based.on.Lab.database)%>%
  filter(Sympotamtic_based.on.Lab.database != "NA")%>%
  summarise(n = n()) %>%
  mutate(freq = n*100 / sum(n))
d5
```

```
fig <- ggplot(d5, aes(biosensor_pcr_concordance, freq, fill=Sympotamtic_based.on.Lab.database, label=n))+
  geom_bar(stat = "identity", colour="black", width = 0.5)+
  labs(y="Proportion", x="CoVSense", fill = "Symptomatic")+
  theme_bw()+
  theme(panel.grid.major = element_blank(), panel.grid.minor = element_blank())+
  guides(fill = guide_legend(reverse=F))+
  ggtitle("")+
  scale_y_continuous(expand = c(0, 0))+
  theme(plot.title = element_text(size=9, hjust = 0.5, face = "bold.italic"))+
  scale_fill_manual(values=c("cornflowerblue","honeydew3"))+
  theme(axis.title = element_text(face = "bold", size = 12),
        legend.title = element_text(face = "bold"), 
        legend.text.align = 0, 
        strip.text.x = element_text(face = "bold"))+
  geom_text(size = 3, position = position_stack(vjust = 0.5))
 
  
fig
```

### 7.4.4 Age

```
d5 <- d4 %>%
  filter(age != "NA")


fig <- ggplot(d5, aes(biosensor_pcr_concordance, age))+
  geom_jitter(aes(color=biosensor_pcr_concordance), position=position_jitter(0.2)) +
  geom_boxplot(alpha = 0)+
  stat_summary(fun.data=data_summary, color="black", size=0.15)+
  labs(y="Age (year)", x="CoVSense", color = "CoVSense")+
  theme_bw()+
  theme(panel.grid.major = element_blank(), panel.grid.minor = element_blank())+
  theme(plot.title = element_text(size=9, hjust = 0.5, face = "bold.italic"))+
  scale_color_manual(values=c("cornflowerblue","lightpink3", "lightsteelblue4"))+
  theme(axis.title = element_text(face = "bold", size = 12),
        legend.title = element_text(face = "bold"), 
        legend.text.align = 0, 
        strip.text.x = element_text(face = "bold"))
 
  
fig
```

```
d5 %>% group_by(biosensor_pcr_concordance)%>%
  summarise_at("age", c(mean, sd))%>%
  rename(mean=fn1, sd=fn2)
```

```
mod <- aov(age ~ biosensor_pcr_concordance, data = d5)
summary(mod)
```

```
##                            Df Sum Sq Mean Sq F value Pr(>F)  
## biosensor_pcr_concordance   2   4051  2025.4   4.275 0.0165 *
## Residuals                 102  48325   473.8                 
## ---
## Signif. codes:  0 '***' 0.001 '**' 0.01 '*' 0.05 '.' 0.1 ' ' 1
```

```
TukeyHSD(mod)
```

```
##   Tukey multiple comparisons of means
##     95% family-wise confidence level
## 
## Fit: aov(formula = age ~ biosensor_pcr_concordance, data = d5)
## 
## $biosensor_pcr_concordance
##             diff        lwr       upr     p adj
## TN-FN  17.441667  -5.222922 40.106255 0.1648966
## TP-FN   5.403955 -16.779521 27.587431 0.8314303
## TP-TN -12.037712 -22.640892 -1.434532 0.0219332
```

### 7.4.5 days post symptom onset

```
d5 <- d4 %>%
  filter(test_day_post_symptom_onset != "NA")


fig <- ggplot(d5, aes(biosensor_pcr_concordance, test_day_post_symptom_onset))+
  geom_jitter(aes(color=biosensor_pcr_concordance), position=position_jitter(0.2)) +
  geom_boxplot(alpha = 0)+
  stat_summary(fun.data=data_summary, color="black", size=0.15)+
  labs(y="Day post symptom onset", x="CoVSense", color = "CoVSense")+
  theme_bw()+
  theme(panel.grid.major = element_blank(), panel.grid.minor = element_blank())+
  theme(plot.title = element_text(size=9, hjust = 0.5, face = "bold.italic"))+
  scale_color_manual(values=c("cornflowerblue","lightpink3", "lightsteelblue4"))+
  theme(axis.title = element_text(face = "bold", size = 12),
        legend.title = element_text(face = "bold"), 
        legend.text.align = 0, 
        strip.text.x = element_text(face = "bold"))
 
  
fig
```

```
d5 %>% group_by(biosensor_pcr_concordance)%>%
  summarise_at("test_day_post_symptom_onset", c(mean, sd))%>%
  rename(mean=fn1, sd=fn2)
```

```
mod <- aov(test_day_post_symptom_onset ~ biosensor_pcr_concordance, data = d5)
summary(mod)
```

```
##                           Df Sum Sq Mean Sq F value Pr(>F)
## biosensor_pcr_concordance  1    0.0   0.018   0.002  0.964
## Residuals                 60  521.9   8.698
```

```
TukeyHSD(mod)
```

```
##   Tukey multiple comparisons of means
##     95% family-wise confidence level
## 
## Fit: aov(formula = test_day_post_symptom_onset ~ biosensor_pcr_concordance, data = d5)
## 
## $biosensor_pcr_concordance
##              diff       lwr      upr     p adj
## TP-FN -0.06315789 -2.814638 2.688322 0.9635304
```

### 7.4.6 Ct values

```
d5 <- d4 %>%
  filter(CT_2 != "NA")


fig <- ggplot(d5, aes(biosensor_pcr_concordance, CT_2))+
  geom_jitter(aes(color=biosensor_pcr_concordance), position=position_jitter(0.2)) +
  geom_boxplot(alpha = 0)+
  stat_summary(fun.data=data_summary, color="black", size=0.15)+
  labs(y="Ct value", x="CoVSense", color = "CoVSense")+
  theme_bw()+
  theme(panel.grid.major = element_blank(), panel.grid.minor = element_blank())+
  theme(plot.title = element_text(size=9, hjust = 0.5, face = "bold.italic"))+
  scale_color_manual(values=c("cornflowerblue","lightpink3", "lightsteelblue4"))+
  theme(axis.title = element_text(face = "bold", size = 12),
        legend.title = element_text(face = "bold"), 
        legend.text.align = 0, 
        strip.text.x = element_text(face = "bold"))
 
  
fig
```

```
d5 %>% group_by(biosensor_pcr_concordance)%>%
  summarise_at("CT_2", c(mean, sd))%>%
  rename(mean=fn1, sd=fn2)
```

```
mod <- aov(CT_2 ~ biosensor_pcr_concordance, data = d5)
summary(mod)
```

```
##                            Df Sum Sq Mean Sq F value Pr(>F)    
## biosensor_pcr_concordance   2   6117  3058.4   185.8 <2e-16 ***
## Residuals                 102   1679    16.5                   
## ---
## Signif. codes:  0 '***' 0.001 '**' 0.01 '*' 0.05 '.' 0.1 ' ' 1
```

```
TukeyHSD(mod)
```

```
##   Tukey multiple comparisons of means
##     95% family-wise confidence level
## 
## Fit: aov(formula = CT_2 ~ biosensor_pcr_concordance, data = d5)
## 
## $biosensor_pcr_concordance
##             diff        lwr         upr     p adj
## TN-FN  12.076667   7.852051  16.3012824 0.0000000
## TP-FN  -3.893333  -8.028271   0.2416043 0.0694322
## TP-TN -15.970000 -17.946403 -13.9935970 0.0000000
```

# 8 **Comparing CoVSense and Panbio**

## 8.1 Figure 5A

Concordance of CoVSense and Panbio SARS-CoV-2 diagnosis in comparison to the gold standard approach using RT-PCR

```
d4 <-  d3 %>%
  group_by(Measurement_cat, Panbio, Covid_PCR_result)%>%
  summarise(n = n()) %>%
  mutate(freq = n*100 / sum(n))%>%
  mutate(Panbio = recode(Panbio,
                         "Neg"="Panbio - Negative",
                          "Pos" = "Panbio - Positive"))
d4
```

```
fig <- ggplot(d4, aes(Measurement_cat, freq, fill=Covid_PCR_result, label=paste0(n,"\n(",round(freq, digits = 1),"%)")))+
  geom_bar(stat = "identity", colour="black", width = 0.5)+
  labs(y="Proportion", x="CoVSense", fill = "PCR")+
  facet_wrap(~Panbio, scales = "free")+
  theme_bw()+
  theme(panel.grid.major = element_blank(), panel.grid.minor = element_blank())+
  guides(fill = guide_legend(reverse=F))+
  scale_y_continuous(expand = c(0, 0))+
  theme(plot.title = element_text(size=9, hjust = 0.5, face = "bold.italic"))+
  scale_fill_manual(values=c("goldenrod3",  "midnightblue"))+
  theme(axis.title = element_text(face = "bold", size = 12),
        legend.title = element_text(face = "bold", size = 14), 
        legend.text.align = 0, 
        strip.text.x = element_text(face = "bold", size = 14))+
  geom_text(size = 3, position = position_stack(vjust = 0.5), color = "white")
 
  
fig
```

## 8.2 Figure 5B

Diagnostic performance of CoVSense and Panbio stratified by virus variant.

### 8.2.1 CoVSense

```
for (i in c("Wildtype", "Variant B.1.1.7")){
  print(paste0("is not ", i))

  m <-  d3%>% filter(Variant != i)
  


  mylogit1 <- glm(Covid_PCR_result ~ Measurement_cat,
                  data = m, family = "binomial")
prob <- as.data.frame(predict(mylogit1,type=c("response")))


  y <- ifelse(m$Covid_PCR_result == "Positive", 1, 0)

  pred <- ifelse(mylogit1$fitted.values < 0.5, 0, 1)


  print(paste0("Accuracy = ", round(Accuracy(pred, y), digits = 2)))
  print(paste0("AUC = ", round(AUC(mylogit1$fitted.values, y), digits = 2)))


  ConfusionMatrix(y_pred = pred, y_true = y)


  print(paste0("F1 for positive state = ", 
               round(F1_Score(y_pred = pred,
                              y_true = y, 
                              positive = "1"), 
                     digits = 2)))
  print(paste0("F1 for negative state = ", 
               round(F1_Score(y_pred = pred, 
                              y_true = y, 
                              positive = "0"), 
                     digits = 2)))


  print(paste0("Recall for positive state = ", 
               round(Recall(y_pred = pred, 
                            y_true = y, 
                            positive = "1"), 
                     digits = 2))) 
  print(paste0("Recall for negative state = ", 
               round(Recall(y_pred = pred, 
                            y_true = y, 
                            positive = "0"), 
                     digits = 2)))
}
```

```
## [1] "is not Wildtype"
## [1] "Accuracy = 0.95"
## [1] "AUC = 0.94"
## [1] "F1 for positive state = 0.94"
## [1] "F1 for negative state = 0.95"
## [1] "Recall for positive state = 0.89"
## [1] "Recall for negative state = 1"
## [1] "is not Variant B.1.1.7"
## [1] "Accuracy = 0.97"
## [1] "AUC = 0.97"
## [1] "F1 for positive state = 0.97"
## [1] "F1 for negative state = 0.98"
## [1] "Recall for positive state = 0.93"
## [1] "Recall for negative state = 1"
```

### 8.2.2 Panbio

```
for (i in c("Wildtype", "Variant B.1.1.7")){
  print(paste0("is not ", i))

  m <-  d3%>% filter(Variant != i)
  


  mylogit1 <- glm(Covid_PCR_result ~ Panbio,
                  data = m, family = "binomial")
prob <- as.data.frame(predict(mylogit1,type=c("response")))


  y <- ifelse(m$Covid_PCR_result == "Positive", 1, 0)

  pred <- ifelse(mylogit1$fitted.values < 0.5, 0, 1)


  print(paste0("Accuracy = ", round(Accuracy(pred, y), digits = 2)))
  print(paste0("AUC = ", round(AUC(mylogit1$fitted.values, y), digits = 2)))


  ConfusionMatrix(y_pred = pred, y_true = y)


  print(paste0("F1 for positive state = ", 
               round(F1_Score(y_pred = pred, 
                              y_true = y, 
                              positive = "1"), 
                     digits = 2)))
  print(paste0("F1 for negative state = ", 
               round(F1_Score(y_pred = pred, 
                              y_true = y, 
                              positive = "0"), 
                     digits = 2)))


  print(paste0("Recall for positive state = ", 
               round(Recall(y_pred = pred, 
                            y_true = y, 
                            positive = "1"), 
                     digits = 2))) 
  print(paste0("Recall for negative state = ", 
               round(Recall(y_pred = pred, 
                            y_true = y, 
                            positive = "0"), 
                     digits = 2)))
}
```

```
## [1] "is not Wildtype"
## [1] "Accuracy = 0.91"
## [1] "AUC = 0.9"
## [1] "F1 for positive state = 0.89"
## [1] "F1 for negative state = 0.92"
## [1] "Recall for positive state = 0.8"
## [1] "Recall for negative state = 1"
## [1] "is not Variant B.1.1.7"
## [1] "Accuracy = 0.79"
## [1] "AUC = 0.75"
## [1] "F1 for positive state = 0.67"
## [1] "F1 for negative state = 0.84"
## [1] "Recall for positive state = 0.5"
## [1] "Recall for negative state = 1"
```

```
df <- data.frame (Test  = c("Biosensor_primary", "Biosensor_variant", "Panbio_primary", "Panbio_variant"),
                  Accuracy = c(0.97,0.95,0.79,0.91),
                  AUC = c(0.97,0.94,0.75,0.90),
                  F1_for_positive = c(0.97,0.94,0.67,0.89),
                  F1_for_negative = c(0.98,0.95,0.84,0.92), 
                  Recall_for_positive = c(0.93,0.89,0.50,0.80),
                  Recall_for_negative = c(1.0,1.0,1.0,1.0))%>%
  gather(Accuracy:Recall_for_negative, key= Parameter, value = Value)%>%
  mutate(Parameter = recode(Parameter,
                           "F1_for_positive" = "F1 (positive)",
                           "F1_for_negative" = "F1 (negative)",
                            "Recall_for_positive" = "Recall (positive)",
                            "Recall_for_negative" = "Recall (negative)"))
df
```

```
df$Test <- factor(df$Test, levels = c("Biosensor_primary", "Biosensor_variant", "Panbio_primary", "Panbio_variant"))
```

```
fig <- ggplot(df, aes(Test, fct_rev(Parameter), fill = Value))+
  geom_tile(color = "white")+
  geom_text(aes(label=paste0(round(Value, digits = 2))))+
  theme_minimal()+
  scale_fill_gradient(low = "cornsilk1", 
                      high = "mediumpurple3", 
                      n.breaks = 4, 
                      limits = c(0.50,1.0))+
  theme(plot.title = element_text(size=12, hjust = 0.5, face = "bold"),
        axis.title = element_blank(),
        legend.title = element_text(face = "bold"), 
        legend.text.align = 0,
        axis.text.x = element_text(face = "bold", 
                                   size = 12, 
                                   angle = 30, 
                                   hjust = 1),
        axis.text.y = element_text(face = "bold", size = 12),
        panel.border = element_blank(), 
        panel.grid.major = element_blank(),
        panel.grid.minor = element_blank())+
        scale_x_discrete(labels=c("Biosensor_primary" = "Wildtype",
                                  "Biosensor_variant" = "Variant B.1.1.7",
                                  "Panbio_primary" = "Wildtype",
                                  "Panbio_variant" = "Variant B.1.1.7"))
  
fig
```

## 8.3 Figure 5C

Concordance of accurate diagnosis of SARS-CoV-2 infection by CoVSense and Panbio in relation to RT-PCR Ct values

```
m <- d3 %>%
  select(CT_cat2, Measurement_cat, Panbio, CT_cat)%>%
  mutate(CoVSense = ifelse(Measurement_cat == "Negative" 
                           & CT_cat2 == "Negative", 
                           "Concordance",
                                 ifelse(Measurement_cat == "Positive" &
                                          CT_cat2 == "Positive",
                                        "Concordance", "Discordance")))%>%
  mutate(Panbio = ifelse(Panbio == "Neg" & CT_cat2 == "Negative",
                         "Concordance",
                                 ifelse(Panbio == "Pos" & 
                                          CT_cat2 == "Positive",
                                        "Concordance", "Discordance")))%>%
  gather(CoVSense,Panbio, key = Test, value = Concordance)%>%
  group_by(CT_cat2, Test, Concordance)%>%
  summarise(n = n()) %>%
  mutate(freq = n*100 / sum(n))%>%
  mutate(CT_cat2 = recode(CT_cat2,
                          "Negative" = "Negative PCR",
                           "Positive" = "Positive PCR"))
  
m
```

```
fig <- ggplot(m, aes(Test, freq, fill=Concordance))+
  geom_bar(stat = "identity", position = position_dodge(), color="black")+
  theme_bw()+
  theme(panel.grid.major = element_blank(), panel.grid.minor = element_blank())+
  facet_wrap(~CT_cat2)+
  scale_fill_brewer(direction=-1)+
  ylab("Concordance with PCR (%)")+
  theme(plot.title = element_text(size=9, hjust = 0.5, face = "bold.italic"))+
  scale_color_manual(
    values=c("cornflowerblue","lightpink3", "lightsteelblue4"))+
  scale_y_continuous(expand = c(0, 0))+
  theme(axis.title = element_text(face = "bold", size = 12),
        legend.title = element_text(face = "bold"), 
        legend.text.align = 0, 
        strip.text.x = element_text(face = "bold", size = 12))
fig
```

```
d2 <- d3 %>%
  select(CT_cat2, Measurement_cat, Panbio, CT_cat)%>%
  mutate(CoVSense = ifelse(Measurement_cat == "Negative" & 
                             CT_cat2 == "Negative", "Concordance",
                                 ifelse(Measurement_cat == "Positive" 
                                        & CT_cat2 == "Positive",
                                        "Concordance", "Discordance")))%>%
  mutate(Panbio = ifelse(Panbio == "Neg" & CT_cat2 == "Negative",
                         "Concordance",
                                 ifelse(Panbio == "Pos" & 
                                          CT_cat2 == "Positive",
                                        "Concordance", "Discordance")))%>%
  gather(CoVSense,Panbio, key = Test, value = Concordance)

d2
```

```
for (i in unique(d2$CT_cat)){
  print(paste0("Ct value = ", i))
  m <- d2 %>%
  filter(CT_cat == i)

 print(dim(m))
 
tbl = table(m$Test, m$Concordance) 
print(chisq.test(tbl))
print(tbl)
prop.table(tbl, 2)
}
```

```
## [1] "Ct value = <25"
## [1] 76  5
## 
##  Pearson's Chi-squared test with Yates' continuity correction
## 
## data:  tbl
## X-squared = 0, df = 1, p-value = 1
## 
##           
##            Concordance Discordance
##   CoVSense          36           2
##   Panbio            35           3
## [1] "Ct value = 25-30"
## [1] 26  5
## 
##  Pearson's Chi-squared test with Yates' continuity correction
## 
## data:  tbl
## X-squared = 4.5139, df = 1, p-value = 0.03362
## 
##           
##            Concordance Discordance
##   CoVSense          12           1
##   Panbio             6           7
## [1] "Ct value = 30-37"
## [1] 28  5
## 
##  Pearson's Chi-squared test with Yates' continuity correction
## 
## data:  tbl
## X-squared = 9.1897, df = 1, p-value = 0.002434
## 
##           
##            Concordance Discordance
##   CoVSense          11           3
##   Panbio             2          12
## [1] "Ct value = >37"
## [1] 80  5
## 
##  Chi-squared test for given probabilities
## 
## data:  tbl
## X-squared = 0, df = 1, p-value = 1
## 
##           
##            Concordance
##   CoVSense          40
##   Panbio            40
```

## 8.4 Figure 5D

Concordance of CoVSense and Panbio in relation to RT-PCR results

```
d2 <- d3 %>%
  select(CT_cat2, Measurement_cat, Panbio, CT_cat)%>%
  mutate(CoVSense = ifelse(Measurement_cat == "Negative" & 
                             CT_cat2 == "Negative", "Concordance",
                                 ifelse(Measurement_cat == "Positive" &
                                          CT_cat2 == "Positive",
                                        "Concordance", "Discordance")))%>%
  mutate(Panbio = ifelse(Panbio == "Neg" & CT_cat2 == "Negative",
                         "Concordance",
                                 ifelse(Panbio == "Pos" & 
                                          CT_cat2 == "Positive",
                                        "Concordance", "Discordance")))%>%
  gather(CoVSense,Panbio, key = Test, value = Concordance)
d2
```

```
for (i in unique(d2$CT_cat2)){
  print(paste0(i))
  m <- d2 %>%
  filter(CT_cat2 == i)

 print(dim(m))
 
tbl = table(m$Test, m$Concordance) 
print(chisq.test(tbl))
tbl
prop.table(tbl, 2)
}
```

```
## [1] "Positive"
## [1] 130   5
## 
##  Pearson's Chi-squared test with Yates' continuity correction
## 
## data:  tbl
## X-squared = 10.242, df = 1, p-value = 0.001373
## 
## [1] "Negative"
## [1] 80  5
## 
##  Chi-squared test for given probabilities
## 
## data:  tbl
## X-squared = 0, df = 1, p-value = 1
```

```
m <- d3 %>%
  select(CT_cat2, Measurement_cat, Panbio, CT_cat)%>%
  mutate(CoVSense = ifelse(Measurement_cat == "Negative" & 
                             CT_cat2 == "Negative", "Concordance",
                                 ifelse(Measurement_cat == "Positive" &
                                          CT_cat2 == "Positive",
                                        "Concordance", "Discordance")))%>%
  mutate(Panbio = ifelse(Panbio == "Neg" & CT_cat2 == "Negative",
                         "Concordance",
                                 ifelse(Panbio == "Pos" & 
                                          CT_cat2 == "Positive",
                                        "Concordance", "Discordance")))%>%
  gather(CoVSense,Panbio, key = Test, value = Concordance)%>%
  group_by(CT_cat, Test, Concordance)%>%
  summarise(n = n()) %>%
  mutate(freq = n*100 / sum(n))%>%
  filter(Concordance == "Concordance")
 
m
```

```
fig <- ggplot(m, aes(CT_cat, freq, fill=Test))+
  geom_bar(stat = "identity", position = position_dodge(), color="black")+
  theme_bw()+
  theme(panel.grid.major = element_blank(), panel.grid.minor = element_blank())+
  scale_fill_brewer(direction=-1, palette = "green")+
  scale_color_brewer(direction=-1)+
  ylab("Concordance with positive PCR (%)")+
  xlab("CT value")+
  theme(plot.title = element_text(size=9, 
                                  hjust = 0.5, 
                                  face = "bold.italic"))+
  scale_y_continuous(expand = c(0, 0))+
  theme(axis.title = element_text(face = "bold", size = 12),
        legend.title = element_text(face = "bold"), 
        legend.text.align = 0, 
        strip.text.x = element_text(face = "bold"))
fig
```

# 9 **Session info**

```
sessionInfo()
```

```
## R version 4.0.3 (2020-10-10)
## Platform: x86_64-apple-darwin17.0 (64-bit)
## Running under: macOS Catalina 10.15.7
## 
## Matrix products: default
## BLAS:   /Library/Frameworks/R.framework/Versions/4.0/Resources/lib/libRblas.dylib
## LAPACK: /Library/Frameworks/R.framework/Versions/4.0/Resources/lib/libRlapack.dylib
## 
## locale:
## [1] en_CA.UTF-8/en_CA.UTF-8/en_CA.UTF-8/C/en_CA.UTF-8/en_CA.UTF-8
## 
## attached base packages:
## [1] stats     graphics  grDevices utils     datasets  methods   base     
## 
## other attached packages:
##  [1] pROC_1.16.2            MLmetrics_1.1.1        OptimalCutpoints_1.1-4
##  [4] ggrepel_0.9.1          epiR_2.0.26            survival_3.2-7        
##  [7] yardstick_0.0.8        workflowsets_0.0.2     workflows_0.2.2       
## [10] tune_0.1.5             rsample_0.1.0          recipes_0.1.16        
## [13] parsnip_0.1.6          modeldata_0.1.0        infer_0.5.4           
## [16] dials_0.0.9            scales_1.1.1           broom_0.7.6           
## [19] tidymodels_0.1.3       forcats_0.5.0          stringr_1.4.0         
## [22] dplyr_1.0.7            purrr_0.3.4            readr_1.3.1           
## [25] tidyr_1.1.3            tibble_3.1.2           ggplot2_3.3.3         
## [28] tidyverse_1.3.0       
## 
## loaded via a namespace (and not attached):
##  [1] colorspace_2.0-0   ellipsis_0.3.2     class_7.3-17       fs_1.5.0          
##  [5] rstudioapi_0.13    listenv_0.8.0      furrr_0.1.0        farver_2.0.3      
##  [9] prodlim_2019.11.13 fansi_0.5.0        lubridate_1.7.9    xml2_1.3.2        
## [13] codetools_0.2-16   splines_4.0.3      knitr_1.29         jsonlite_1.7.2    
## [17] dbplyr_1.4.4       compiler_4.0.3     httr_1.4.2         backports_1.1.8   
## [21] assertthat_0.2.1   Matrix_1.2-18      fastmap_1.1.0      cli_3.0.1         
## [25] htmltools_0.5.2    tools_4.0.3        gtable_0.3.0       glue_1.4.2        
## [29] Rcpp_1.0.7         cellranger_1.1.0   DiceDesign_1.9     vctrs_0.3.8       
## [33] nlme_3.1-149       iterators_1.0.12   timeDate_3043.102  gower_0.2.2       
## [37] xfun_0.16          globals_0.14.0     rvest_0.3.6        lifecycle_1.0.0   
## [41] future_1.24.0      MASS_7.3-53        ipred_0.9-9        hms_0.5.3         
## [45] parallel_4.0.3     RColorBrewer_1.1-2 yaml_2.2.1         pander_0.6.3      
## [49] rpart_4.1-15       stringi_1.5.3      highr_0.8          foreach_1.5.0     
## [53] lhs_1.1.1          lava_1.6.7         rlang_0.4.11       pkgconfig_2.0.3   
## [57] evaluate_0.14      lattice_0.20-41    labeling_0.4.2     tidyselect_1.1.1  
## [61] parallelly_1.30.0  plyr_1.8.6         magrittr_2.0.1     R6_2.5.0          
## [65] generics_0.1.0     DBI_1.1.0          mgcv_1.8-33        pillar_1.6.1      
## [69] haven_2.3.1        withr_2.4.2        nnet_7.3-14        modelr_0.1.8      
## [73] crayon_1.4.1       utf8_1.2.1         rmarkdown_2.3      grid_4.0.3        
## [77] readxl_1.3.1       blob_1.2.1         reprex_0.3.0       digest_0.6.27     
## [81] munsell_0.5.0      GPfit_1.0-8        BiasedUrn_1.07
```
